# Synthesis of phenanthridines via a novel photochemically-mediated cyclization and application to the synthesis of triphaeridine

**DOI:** 10.3762/bjoc.17.152

**Published:** 2021-09-08

**Authors:** Songeziwe Ntsimango, Kennedy J Ngwira, Moira L Bode, Charles B de Koning

**Affiliations:** 1 Molecular Sciences Institute, School of Chemistry, University of the Witwatersrand, PO Wits 2050, Johannesburg, South Africa

**Keywords:** aromatic compounds, cyclization, iminyl radical, phenanthridines, radical cation, synthesis, UV irradiation

## Abstract

Readily synthesized biphenyl-2-carbaldehyde *O*-acetyl oximes were exposed to UV radiation affording phenanthridines. The scope and limitations of this novel reaction were explored. For example, exposure of 2',3'-dimethoxy-[1,1'-biphenyl]-2-carbaldehyde *O*-acetyl oxime to UV radiation afforded 4-methoxyphenanthridine in 54% yield. This methodology was applied to the synthesis of trisphaeridine to afford the product in four linear steps in an overall yield of 6.5% from 1-bromo-2,4,5-trimethoxybenzene.

## Introduction

**Figure 1:**
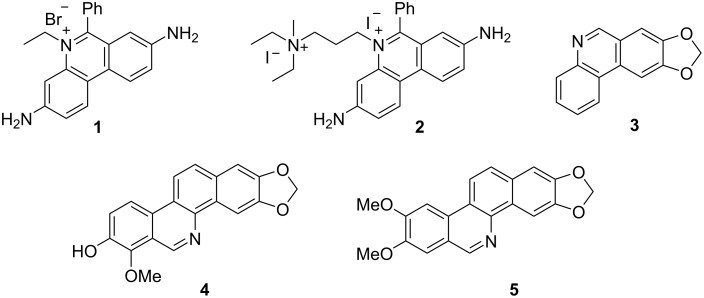
Biologically active phenanthridines.

Phenanthridine derivatives have captivated synthetic chemists and biologists alike since the 1960s due to their efficient DNA binding, antitumour and antiparasitic activities [1]. Such compounds include the DNA and RNA-fluorescent marker ethidium bromide (**1**), the cell viability probe propidium iodide (**2**) and the naturally occurring antiparasitic compounds, trisphaeridine (**3**), decarine (**4**) and nortidiene (**5**) (Figure 1).

The importance of the phenanthridine framework has inspired the development of numerous synthetic strategies to access these compounds. These include the classical Pictet and Ankersmit pyrolysis of *N*-benzylideneaniline [2], rhodium-mediated alkyne [2 + 2 + 2] cycloaddition reactions [3], and the palladium-catalysed aerobic domino Suzuki coupling/Michael addition reaction [4]. The most attractive and common strategies to phenanthridines rely on intramolecular cyclizations of various *ortho*-functionalized biaryl precursors furnishing the central ring through C–C bond formation. Indeed, this strategy has been employed for decades in reactions such as dehydrative cyclization of acyl-*O*-aminobiphenyls at very high temperatures (the Pictet–Hubert reaction and Morgan–Walls reaction) [5–6], which is also reflected in modern methods, such as ionic liquid- and transition metal-catalyzed and other metal-free transformations [1].

**Figure 2:**
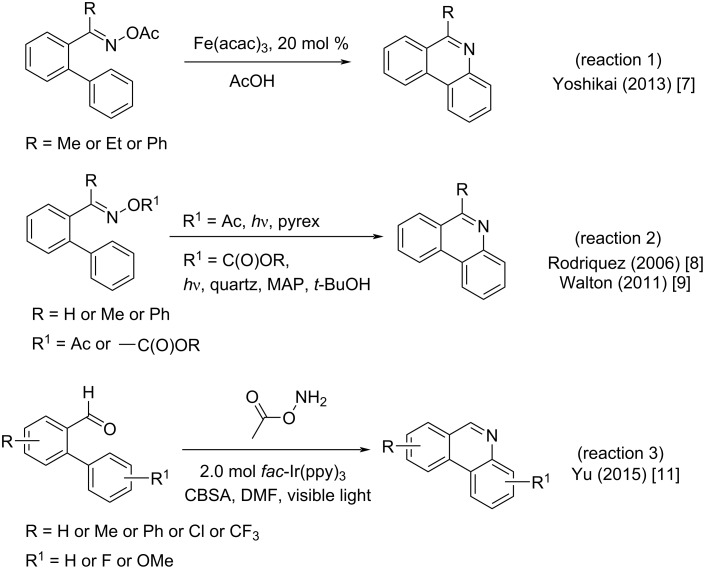
Synthetic routes to phenanthridines via iminyl radicals.

A strategically diverse route to phenanthridines involves intramolecular cyclization of biaryl oximes, allowing for the formation of a new C–N bond. Such a strategy was explored by Deb and Yoshikai in the Fe(III)-catalyzed intramolecular cyclization of *O*-acetyloximes (Figure 2, reaction 1). However, this transformation was limited to ketoximes and yielded only 6-substituted phenanthridines [7]. Applying photochemical conditions, Rodrıguez and Walton were able to use benzaldehydes utilizing a similar intramolecular cyclization of *O*-acetyloximes to afford phenanthridines. (Figure 2, reaction 2) [8–9]. For these transformations, the reaction is thought to go via the transient iminyl radical [10]. More recently, Yu has reported the use of in situ-derived *O*-acyl oximes from benzaldehydes that when subjected to visible light photoredox-catalyzed cyclizations also afforded phenanthridines (Figure 2, reaction 3) [11].

**Scheme 1:**
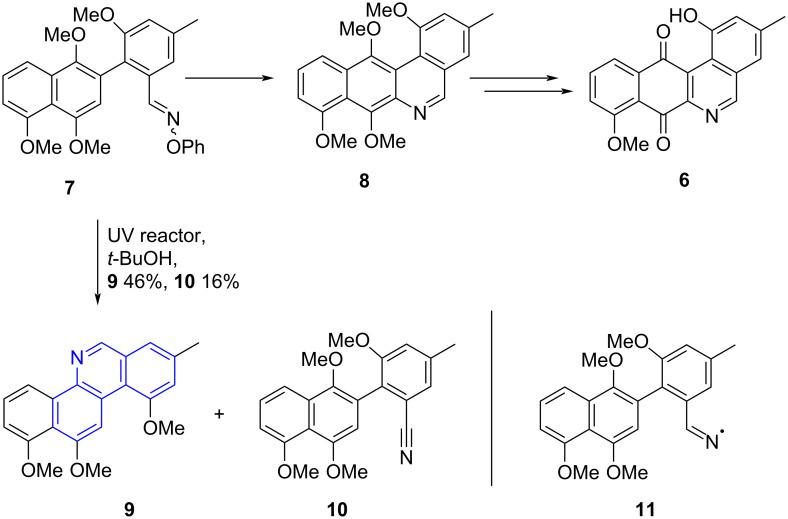
Previous unexpected synthesis of the phenanthridine framework.

Inspired by the Rodrıguez and Walton approach, we sought to synthesize the nitrogen analogue of an angucycline known as phenanthroviridone **6**, [12] from oxime **7** (Scheme 1). Based on the results reported by Rodrıguez and Walton we envisaged that under these photochemcial conditions, intermediate **8** would be formed. Contrary to the expected results, in our labs exposure of oxime **7** to UV irradiation yielded phenanthridine **9** as the main product alongside the nitrile **10** in lower yields [13]. We hypothesized that oxime **7** undergoes a homolytic cleavage of the N–O bond giving the iminyl radical **11** [14] followed by an intramolecular cyclization with concomitant expulsion of the *ortho*-methoxy group, liberating phenanthridine **9**.

To the best of our knowledge, such a reaction in which the aromatic methoxy group is a leaving group resulting in the formation of phenanthridines is unprecedented. As is outlined in this paper we set out to investigate the scope and limitations of this reaction resulting in the formation of the phenanthridine skeleton. We also report on the application of this methodology to the synthesis of the simple phenanthridine natural product trisphaeridine (**3**).

## Results and Discussion

**Scheme 2:**
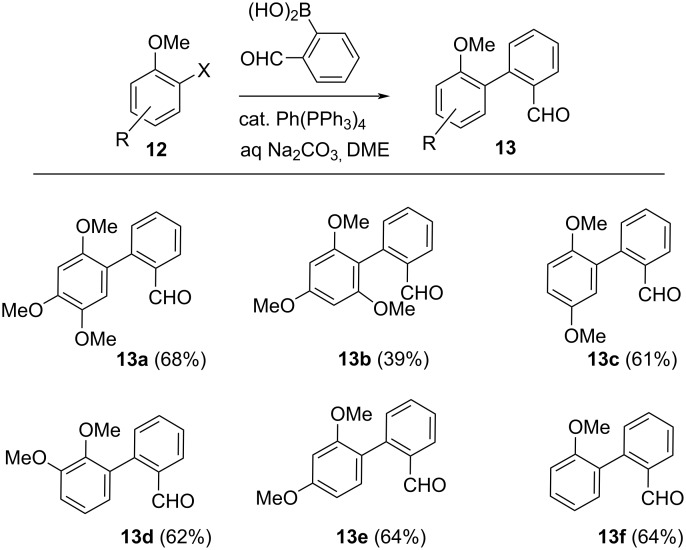
Synthesis of biaryl benzaldehydes.

To investigate the scope and limitations of the reaction we initially opted to prepare a number of biaryl substrates possessing one aromatic electron accepting ring possessing a variety of methoxy substituents in a range of positions. In all cases at least one methoxy substituent was positioned *ortho-* to the biaryl axis. The other aromatic ring had to contain an oxime ester, which would form an iminyl electron donor, as shown in Scheme 2. As illustrated substrates **13a**–**f** were accessed using the Suzuki–Miyaura cross-coupling reaction of a variety of halogenated methoxybenzene-containing compounds with 2-(formylphenyl)boronic acids in generally good yields [15].

**Scheme 3:**
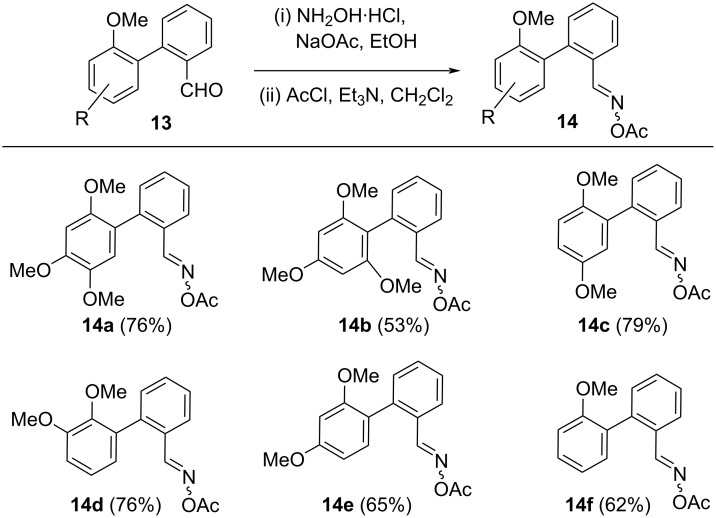
Synthesis of biaryl oximes.

As a next step, the biaryl aldehydes **13a**–**f** were converted into their corresponding oximes **14a**–**f** in good yields as a mixture of (*E*) and (*Z*)-isomers by reaction initally with hydroxylamine followed by acetyl chloride (Scheme 3).

**Scheme 4:**
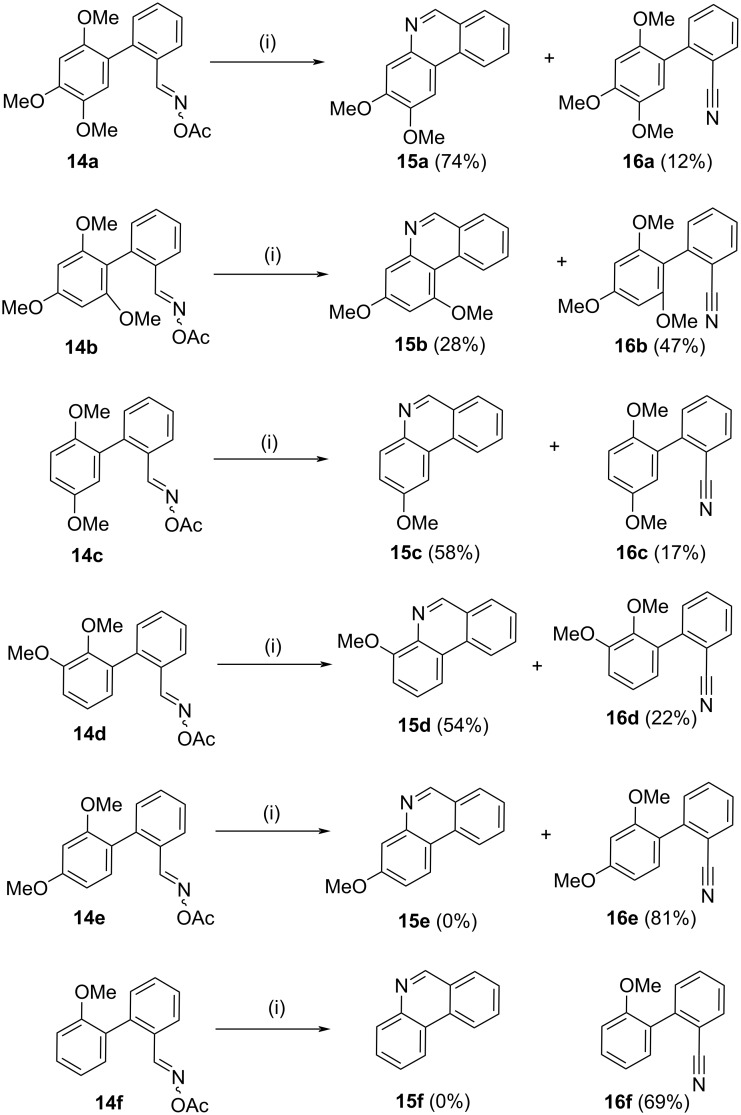
Synthesis of phenanthridines**.** Reagents and conditions (i) UV irradiation (450 W medium pressure Hg lamp), *t*-BuOH, 3 h.

The next step was the key photochemical ring closure to determine the scope and limitations of the novel phenanthridine-forming reaction from biaryl oximes (i.e., **14** → **15**). Exposure of the substrates **14a–f** to UV irradiation utilizing a 450 W Hg medium pressure lamp and through a quartz filter, afforded in most cases the phenanthridines. The first four cases, as shown in Scheme 4, resulted in the desired phenanthridines (**15a–d**) in varying yields, although in all cases the related benzonitriles (**16a–d**) were obtained.

To be effective, the desired cyclization needs a second methoxy substituent *ortho-* or *para*- to the leaving group, which in this case is an aromatic methoxy substituent.

**Figure 3:**
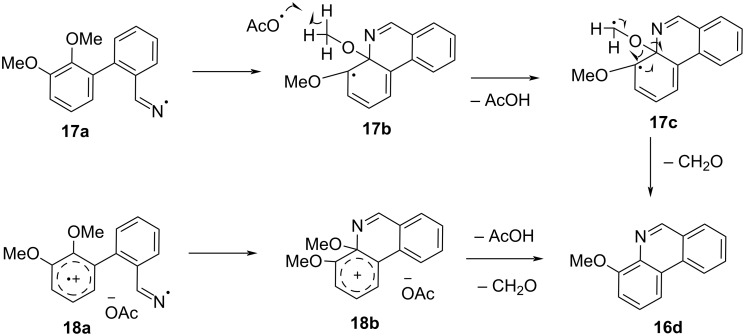
Two possible mechanistic routes and intermediates in the synthesis of phenanthridines.

Based on these results we suggest two possible mechanisms for these transformations. Both would proceed through an intermediary electron donor iminyl radical. In fact, iminyl radicals have been identified and studied by EPR spectroscopy in a number of related processes involving oxime derivatives [14]. As an example utilizing substrate **14d**, intermediates such as the iminyl radical **17a** and the ring closed intermediate **17b** (Figure 3) would allow for the stabilization of the radical by the methoxy substituent in the *ortho* position to the departing substituent. The methoxy substituent then presumably loses a hydrogen radical to form acetic acid and intermediate **17c**. Further loss of formaldehyde would restore aromaticity and furnish the desired phenanthridine **16d**.

As the aromatic substrates **14a–d** contain an electron-rich aromatic ring, the second possibility is the reaction proceeds via the oxidation of the methoxy-containing aromatic ring to afford intermediate **18a**. The reaction could then proceed to afford intermediate **18b**. Once intermediate **18b** is formed an H^+^ is abstracted by an acetate ion from the methoxy substituent again forming formaldehyde and acetic acid, along with subsequent quenching of the cation to restore aromaticity. Examples of photoredox-catalyzed cation radical accelerated reactions with the aromatic methoxy group as a leaving group have been documented [16].

Our previous research, as shown in Scheme 1, indicated that exposure of the oxime ether **7** to UV radiation resulted in the formation of the phenanthridine **8**. Attempts to synthesize the related oxime ethers from biaryl compounds **13a**, **13c** and **13e** with *O*-phenylhydroxylamine as reagent were not successful and only resulted in the formation of the benzonitriles **16a**, **16c** and **16e**. Presumably, the oximes were formed but were unstable and the facile elimination of phenol took place to liberate the benzonitriles.

**Scheme 5:**
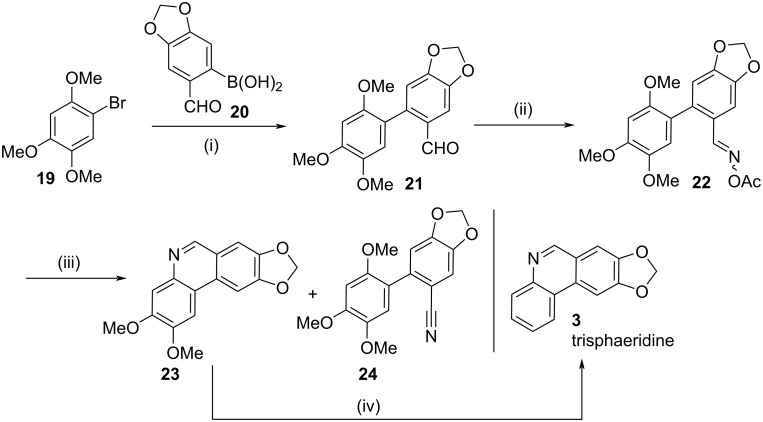
Synthesis of trisphaeridine**.** Reagents and conditions (i) cat. Pd(PPh_3_)_4_, aq Na_2_CO_3_, DME, reflux, Ar, overnight, 58%; (ii) (a) NH_2_OH·HCl, NaOAc, EtOH/H_2_O (1:1), 35 °C, 30 min, (b) AcCl, pyr, rt, 3 h, 74% (for the two steps); (iii) UV irradiation, (450 W medium pressure Hg lamp), *t*-BuOH, rt, 1 h, **23** 41% and **24** 53%; (iv) (a) CAN, MeCN/H_2_O (2:1), 0 °C, 30 min, (b) Zn, NaCl, AcOH, 150, 3 h, 37% (overall for the two steps).

Finally, the use of this methodology was demonstrated to be useful for the synthesis of the natural product trisphaeridine (**3**) [17]. Exposure of 1-bromo-2,4,5-trimethoxybenzene (**19**) to Suzuki–Miyaura coupling reaction conditions with boronic acid **20** resulted in the formation of aldehyde **21** (Scheme 5). Treatment of **21** with hydroxylamine hydrochloride and then acetyl chloride afforded oxime **22**. Next was the key photochemical step. Exposure of **22** to UV irradiation resulted in the formation of the desired phenanthridine **23** in a disappointing yield of 41%, together with the benzonitrile **24**. Phenanthridine **23** was then treated with CAN followed by zinc in acetic acid which resulted in the synthesis of trisphaeridine (**3**) in five steps in a moderate overall yield of 6.5% commencing from **19**.

## Conclusion

In summary, a novel UV light-mediated method for the synthesis of phenanthridines from biaryl oximes has been discovered. A key highlight includes having a methoxy substituent as the leaving group for the formation of phenanthridines. Structure**–re**activity relationship studies also indicated that an *ortho-* or *para-*methoxy group must be present to stabilize the incipient radical or radical cation intermediate. We propose that the reaction is proceeding by means of the initial generation of an iminyl radical that cyclizes onto the electron-rich aromatic ring or through the formation of a radical cation on the electron-rich benzene ring. Finally, the methodology has successfully been applied to synthesizing the natural product trisphaeridine.

## Experimental

For general experimental procedures, please consult Supporting Information File 1.

### Suzuki coupling: general procedure

To a deoxygenated 0.25 M solution of arylboronic acid (1.2 equiv) in DME stirring under argon was added aryl bromide (1.0 equiv), Pd(PPh_3_)_4_ (10 mol %) and deoxygenated 2 M aqueous solution of Na_2_CO_3_ (4.0 equiv) in water. The resulting suspension was heated to reflux for 18 h. The resulting solution was allowed to cool to room temperature and was quenched with water (30 mL). The solution was extracted with EtOAc (3 × 50 mL). The combined organic layers were dried over MgSO_4_. The solvent was removed under reduced pressure and the resulting crude biaryl carbonyls were purified using silica gel column chromatography (using EtOAc/hexane mixtures as eluent).

Experimental details for the preparation of compounds **13a–f** can be found in Supporting Information File 1.

### Oxime ester formation: general procedure

To a solution of biaryl carbonyl (1.0 equiv) in EtOH (0.1 M) was added hydroxylamine hydrochloride (2.0 equiv) and sodium acetate (2.0 equiv). The resulting suspension was stirred under reflux for 18 h. The contents of the flask were allowed to cool to room temperature upon which a precipitate formed. The solvent was filtered and the resulting white solid was dissolved in EtOAc and washed successively with water and brine. The organic layer was dried over MgSO_4_ and the volatiles were removed under reduced pressure. The resulting biaryl oximes were used without further purification.

A two-necked flask equipped with a magnetic bar and a dropping funnel was charged with a biaryl oxime (1.0 equiv), dry CH_2_Cl_2_ and trimethylamine (2.0 equiv). The solution was cooled to 0 °C in an ice bath and a solution of acetyl chloride (2.0 equiv) in dry CH_2_Cl_2_ (2.75 M) was added dropwise for 15 min. The ice bath was removed and the resulting solution was allowed to slowly warm to room temperature. The reaction mixture was stirred overnight. Water (30 mL) was added and the layers were separated. The aqueous layer was extracted with CH_2_Cl_2_ (3 × 30 mL). The combined organic layer was washed with saturated NaHCO_3_ solution (10 mL) and brine (10 mL). The solution was dried over MgSO_4_ and the solvent was evaporated under reduced pressure. The resulting crude oxime esters were purified by silica gel column chromatography (using EtOAc/hexane mixtures as eluent).

Experimental details for the preparation of compounds **14a–f** can be found in Supporting Information File 1.

### UV cyclization of oxime ester derivatives: general procedure

A quartz UV reactor was charged with oxime ester and *tert*-butyl alcohol (5 mL) and the solution was degassed for 30 min. The solution was then subjected to UV irradiation (450 W medium pressure Hg lamp, through a quartz filter) for 3 h. The crude residue was purified by silica gel column chromatography (using EtOAc/hexane mixtures as eluent).

Experimental details for the preparation of compounds **15a–d** and **16a–f** as well as compounds **23** and **24** can be found in Supporting Information File 1.

### Trisphaeridine (**3**)

To a solution of 2,3-dimethoxy-[1,3]dioxolo[4,5-*j*]phenanthridine (**23**, 63 mg, 0.22 mmol) in acetonitrile (3 mL) was slowly added a solution of ceric ammonium nitrate (356 mg, 0.65 mmol) in water (1.5 mL) at 0 °C. The resulting solution was stirred at the same temperature for 30 min and then was quenched with ice-water (1.5 mL). A precipitate formed and was filtered, and washed with water. The solid was then taken up with ethyl acetate, dried over MgSO_4_ and the volatiles were removed under reduced pressure. [1,3]Dioxolo[4,5-*j*]phenanthridine-2,3-dione was obtained as a black solid (35 mg, yield 63%), which was used in the next step without further purification. In a high-pressure tube equipped with a stirrer bar and [1,3]dioxolo[4,5-*j]*phenanthridine-2,3-dione (35 mg, 0.14 mmol), zinc (37 mg, 0.56 mmol), NaCl (33 mg, 0.55 mmol) and acetic acid (5 ml) were added. The tube was sealed and the mixture was stirred at 150 °C for 3 h. The reaction was neutralized with a saturated aqueous solution of sodium bicarbonate before the mixture was extracted with ethyl acetate (3 × 5 mL). The combined organic layer was dried over MgSO_4_, and the solvent was removed under reduced pressure. The crude product was purified by column chromatography (eluent; 5–50% EtOAc/hexane). Trisphaeridine **3** was obtained as a cream solid (19 mg, 59%). ^1^H NMR (400 MHz, CDCl_3_) δ 9.08 (s, 1H, ArH), 8.36 (d, *J* = 8.2 Hz, 1H, ArH), 8.15 (d, *J* = 8.1 Hz, 1H, ArH), 7.89 (s, 1H, ArH), 7.69 (t, *J* = 7.5 Hz, 1H, ArH), 7.62 (t, *J* = 7.6 Hz, 1H, ArH), 7.32 (s, 1H, ArH), 6.16 (s, 2H, Ar-CH_2_); ^13^C NMR (101 MHz, CDCl_3_) δ 151.7, 151.5, 148.2, 144.0, 130.3, 129.9, 128.0, 126.7, 124.3, 123.0, 122.0, 105.5, 101.9, 99.9 ppm [18].

## Supporting Information

File 1 Experimental and analytical data.
