# Supplementary material for: Synthesis of phenanthridines via a novel photochemically-mediated cyclization and application to the synthesis of triphaeridine
Source: Beilstein J Org Chem. 2021 Sep 8;17:2340–7. doi: 10.3762/bjoc.17.152 (PMC8450941; doi:10.3762/bjoc.17.152)

BEILSTEIN JOURNAL OF ORGANIC CHEMISTRY

Supporting Information

for

Synthesis of phenanthridines via a novel photochemically-mediated cyclization and application to the synthesis of triphaeridine

Songeziwe Ntsimango, Kennedy J. Ngwira, Moira L. Bode and Charles B. de Koning

Beilstein J. Org. Chem. **2021,** *17,* 2340–2347. doi:10.3762/bjoc.17.152

Experimental and analytical data

License and Terms: This is a supporting information file under the terms of the Creative Commons Attribution License (https://creativecommons.org/licenses/by/4.0). Please note that the reuse, redistribution and reproduction in particular requires that the author(s) and source are credited and that individual graphics may be subject to special legal provisions.
The license is subject to the *Beilstein Journal of Organic Chemistry* terms and conditions: (https://www.beilstein-journals.org/bjoc/terms)

Table of Contents

General experimental procedures Page S2

Details of the preparation of compounds **13a**–**13f**, **21**, **14a**–**14f**, **22**, **15a**–**15d**, **16a**–**16f** and **23**, **24** Page S3–S15

References Page S16–S17

^{1}H NMR and ^{13}C NMR for compound **13a** Page S18

^{1}H NMR and ^{13}C NMR for compound **13b** Page S19

^{1}H NMR and ^{13}C NMR for compound **13c** Page S20

^{1}H NMR and ^{13}C NMR for compound **13d** Page S21

^{1}H NMR and ^{13}C NMR for compound **13e** Page S22

^{1}H NMR and ^{13}C NMR for compound **13f** Page S23

^{1}H NMR and ^{13}C NMR for compound **21** Page S24

^{1}H NMR and ^{13}C NMR for compound **14a** Page S25

^{1}H NMR and ^{13}C NMR for compound **14b** Page S26

^{1}H NMR and ^{13}C NMR for compound **14c** Page S27

^{1}H NMR and ^{13}C NMR for compound **14d** Page S28

^{1}H NMR and ^{13}C NMR for compound **14e** Page S29

^{1}H NMR and ^{13}C NMR for compound **14f** Page S30

^{1}H NMR and ^{13}C NMR for compound **22** Page S31

^{1}H NMR and ^{13}C NMR for compound **16a** Page S32

^{1}H NMR and ^{13}C NMR for compound **16b** Page S33

^{1}H NMR and ^{13}C NMR for compound **15c** Page S34

^{1}H NMR and ^{13}C NMR for compound **16c** Page S35

^{1}H NMR and ^{13}C NMR for compound **15d** Page S36

^{1}H NMR and ^{13}C NMR for compound **16d** Page S37

^{1}H NMR and ^{13}C NMR for compound **16e** Page S38

^{1}H NMR and ^{13}C NMR for compound **16f** Page S39

^{1}H NMR and ^{13}C NMR for compound **23** Page S40

^{1}H NMR and ^{13}C NMR for compound **24** Page S41

^{1}H NMR and ^{13}C NMR for compound **3** Page S42

General experimental procedures:

Solvents utilized for chromatography (ethyl acetate and *n*-hexane) were distilled prior to use by means of conventional distillation processes. The solvents employed in reactions were first dried over the suitable drying agent, followed by distillation under an inert atmosphere (argon or nitrogen gas). Acetonitrile and dichloromethane were distilled over calcium hydride, whereas tetrahydrofuran was distilled over sodium with benzophenone as an indicator. Toluene was distilled over sodium. All the required chemicals or reagents were obtained from FLUKA, SIGMA ALDRICH or MERCK and were used without further purification.

Normal chromatography was performed with silica gel 60 (Macherey-Nagel, particle size 0.063–0.200 mm) adsorbent, with both isocratic and gradient eluent systems being employed. Thin layer chromatography (TLC) of the compounds was executed on Macherey-Nagel Alugram Silica G/UV254 plates pre-coated with 0.25 mm silica gel 60. The TLC plates were viewed under UV light (254 nm and 366 nm).

Nuclear magnetic resonance (NMR) spectra were recorded on either a Bruker AVANCE 300 MHz or Bruker AVANCE 400 MHz, Bruker AVANCE III 500 MHz spectrometer. All chemical shift values are reported in parts per million referenced against tetramethylsilane which is given an assignment of zero parts per million. Coupling constants (*J*-values) are given in Hertz (Hz).

The infrared spectra were recorded on a Bruker Tensor 27 standard system spectrometer. Measurements were made by loading the sample directly onto a diamond cell. The measurements are reported on the wavenumber scale (cm^{-1}).

Melting points were determined on a Reichert hot-stage microscope, and remain uncorrected. All crystalline compounds were recrystallized in the appropriate solvents prior to melting point determination. Microwave reactions were conducted in a CEM Discover microwave.

High resolution mass spectra were obtained with a Waters-LCT-Premier mass spectrometer. The sample was dissolved in methanol to a concentration of 2 ng/µL and introduced by direct infusion. The ionization mode was electrospray positive with a capillary voltage of 2500 V and a desolvation temperature of 250 °C using nitrogen gas at 250 L/h.

Experimental details for the preparation of compounds 13a–13f and compound 21

2',4',5'-Trimethoxy-[1,1'-biphenyl]-2-carbaldehyde (13a)

The title compound **13a** was prepared from 1-bromo-2,4,5-trimethoxybenzene (0.44 g, 1.62 mmol, 1.0 equiv), (2-formylphenyl)boronic acid (0.32 g, 2.02 mmol, 1.2 equiv), tetrakis(triphenylphosphine)palladium(0) (0.20 g, 0.17 mmol, 0.1 equiv) and a 2M aqueous solution of Na_2CO_3 (0.71 g, 3.3 mL, 6.72 mmol, 4.0 equiv). The product was obtained as yellow solid (0.31 g, yield = 68%). **M.p.** 140–143 °C. **FTIR:** $\tilde{\nu}$ = 2997.5 (C–H stretch), 1691.1 (C=O), 1596.5 (C=C), 1149.7 (C–O) cm^{-1}. **^{1}H NMR** (300 MHz, $CDCl_3$): δ = 9.80 (s, 1H, ArCO), 7.98 (dd, *J* = 7.8, 1.5, 1H, ArH), 7.63 (td, *J* = 7.5, 1.5, 1H, ArH), 7.45 (tt, *J* = 7.5, 1.1, 1H, ArH), 7.36 (dd, *J* = 7.7, 1.3, 1H, ArH), 6.84 (s, 1H, ArH), 6.61 (s, 1H, ArH), 3.96 (s, 3H, OMe), 3.87 (s, 3H, OMe), 3.69 (s, 3H, OMe). **^{13}C NMR** (75 MHz, $CDCl_3$): δ = 192.8, 150.9, 150.2, 143.5, 141.6, 134.2, 133.6, 131.2, 127.5, 126.7, 118.0, 115.0, 97.4, 56.7, 56.2 (× 2) ppm. **HRMS** (ESI^+): calcd. for $C_{16}H_{17}O_4$ $[M + H]^+$ 273.1127; found $[M + H]^+$ 273.1123; *m/z* (%) = 273.1127 (100) $[M + H]^+$, 257.1174 (20), 249.0865 (50) [1].

2',4',6'-Trimethoxy-[1,1'-biphenyl]-2-carbaldehyde (13b)

The title compound **13b** was prepared from 1-bromo-2,4,6-trimethoxybenzene (0.41 g, 1.51 mmol, 1.0 equiv), (2-formylphenyl)boronic acid (0.27 g, 1.81 mmol, 1.2 equiv), tetrakis(triphenylphosphine)palladium(0) (0.20 g, 0.17 mmol, 0.1 equiv) and an aqueous 2M solution of Na_2CO_3 (0.71 g, 3.3 mL, 6.72 mmol, 4.0 equiv). The product was obtained as a yellow solid (0.17 g, yield = 39%). **M.p.** 132–134 °C. FTIR: $\tilde{\nu}$ = 2935.0 (C–H stretch), 1681.1 (C=O), 1508.4 (C=C), 1026.3 (C–O) cm^{-1}. **^{1}H NMR** (300 MHz, $CDCl_3$): δ = 9.74 (d, *J* = 0.9, 1H, ArCO), 7.98 (ddd, *J* = 7.8, 1.6, 0.5, 1H, ArH), 7.60 (td, *J* = 7.5, 1.5, 1H, ArH), 7.42 (tt, *J* = 7.5, 1.1, 1H, ArH), 7.32 (ddd, *J* = 7.7, 1.3,

0.6, 1H, ArH), 6.23 (s, 2H, ArH), 3.88 (s, 3H, OMe), 3.69 (s, 6H, OMe). **^{13}C NMR** (75 MHz, $CDCl_3$) δ = 193.1, 161.7, 158.5, 138.1, 134.6, 133.2, 132.8, 127.3, 126.5, 107.5, 90.7, 55.7, 55.4 ppm. **HRMS** (ESI$^+$): calcd. for $C_{16}H_{17}O_4$ [M + H]$^+$ 273.1127; found [M + H]$^+$ 273.1123; *m/z* (%) = 295.0938 (30), 273.1127 (100) [M + H]$^+$ [2].

2',5'-Dimethoxy-[1,1'-biphenyl]-2-carbaldehyde (13c)

The title compound **13c** was prepared from 2-bromo-1,4-dimethoxybenzene (0.34 g, 1.51 mmol, 1.0 equiv), (2-formylphenyl)boronic acid (0.28 g, 1.82 mmol, 1.2 equiv), tetrakis(triphenylphosphine)palladium(0) (0.17 g, 0.15 mmol, 0.1 equiv) and a 2M aqueous solution of Na_2CO_3 (0.64 g, 2.8 mL, 6.04 mmol, 4.0 equiv). (0.22 g, yield = 61%). **^{1}H NMR** (500 MHz, $CDCl_3$): δ = 9.69 (d, *J* = 0.8, 1H, ArCO), 7.87 (dd, *J* = 7.8, 1.5, 1H, ArH), 7.51 (td, *J* = 7.5, 1.5, 1H, ArH), 7.35 (tt, *J* = 7.6, 1.1, 1H, ArH), 7.24 (dd, *J* = 7.7, 1.2, 1H, ArH), 6.82 (dd, *J* = 8.9, 3.0, 1H, ArH), 6.78 (s, 1H, ArH), 6.77–6.75 (m, 1H, ArH), 3.68 (s, 3H, OMe), 3.54 (s, 3H, OMe). **^{13}C NMR** (126 MHz, $CDCl_3$): δ = 192.5, 153.8, 150.7, 141.6, 134.0, 133.7, 131.1, 127.9, 127.7, 126.6, 117.3, 114.4, 111.8, 55.9, 55.8 [3].

2',3'-Dimethoxy-[1,1'-biphenyl]-2-carbaldehyde (13d)

The title compound **13d** was prepared from 1-bromo-2,3-dimethoxybenzene (0.31 g, 1.51 mmol, 1.0 equiv), (2-formylphenyl)boronic acid (0.26 g, 2.02 mmol, 1.2 equiv), tetrakis(triphenylphosphine)palladium(0) (0.20 g, 0.17 mmol, 0.1 equiv) and a 2M aqueous solution of Na_2CO_3 (0.71 g, 3.3 mL, 6.72 mmol, 4.0 equiv). The product was obtained as a yellow solid (0.22 g, yield = 62%). **^{1}H NMR** (500 MHz, $CDCl_3$): δ = 9.77 (s, 1H, ArCO), 7.94 (dd, *J* = 7.8, 1.5, 1H, ArH), 7.55 (td, *J* = 7.5, 1.5, 1H, ArH), 7.41 (tt, *J* = 7.6, 1.1, 1H, ArH), 7.32 (dd, *J* = 7.6, 1.3, 1H, ArH), 7.07 (t, *J* = 7.9, 1H, ArH), 6.93 (dd, *J* = 8.2, 1.5, 1H, ArH), 6.81 (dd, *J* = 7.7, 1.5, 1H, ArH), 3.83 (s, 3H, OMe),

3.39 (s, 3H, OMe). **^{13}C NMR** (126 MHz, $CDCl_3$): δ = 192.2, 152.7, 146.5, 141.4, 133.8, 133.4, 132.2, 131.1, 127.9, 126.9, 124.3, 123.1, 112.7, 60.4, 56.0 ppm [4].

2',4'-Dimethoxy-[1,1'-biphenyl]-2-carbaldehyde (13e)

The title compound **13e** was prepared from 1-bromo-2,4-dimethoxybenzene (0.83 g, 3.84 mmol, 1.0 equiv), (2-formylphenyl)boronic acid (0.69 g, 4.61 mmol, 1.2 equiv), tetrakis(triphenylphosphine)palladium(0) (0.44 g, 0.38 mmol, 0.1 equiv) and a 2M aqueous solution of Na_2CO_3 (1.63 g, 4.62 mL, 15.4 mmol, 4.0 equiv). Yellow oil (0.59 g, yield = 64%). **^{1}H NMR** (500 MHz, $CDCl_3$) δ 9.69 (s, 1H, ArCO), 7.87 (dd, *J* = 7.8, 1.5, 1H, ArH), 7.51 (td, *J* = 7.5, 1.5, 1H, ArH), 7.35 (tt, *J* = 7.6, 1.1, 1H, ArH), 7.24 (dd, *J* = 7.7, 1.2, 1H, ArH), 6.82 (dd, *J* = 8.9, 3.0, 1H, ArH), 6.78 (s, 1H, ArH), 6.76 (d, *J* = 3.1, 1H, ArH), 3.68 (s, 3H, OMe), 3.54 (s, 3H, OMe). **^{13}C NMR** (126 MHz, $CDCl_3$): δ = 192.5, 153.8, 150.7, 141.6, 134.0, 133.7, 131.1, 127.9, 127.7, 126.6, 117.3, 114.4, 111.8, 55.9, 55.8 ppm [5].

2'-Methoxy-[1,1'-biphenyl]-2-carbaldehyde (13f)

The title compound **13f** was prepared from 1-bromo-2-methoxybenzene (0.28 g, 1.51 mmol, 1.0 equiv), (2-formylphenyl)boronic acid (0.25 g, 1.82 mmol, 1.2 equiv), tetrakis(triphenylphosphine)palladium(0) (0.17 g, 0.15 mmol, 0.1 equiv) and a 2M aqueous solution of Na_2CO_3 (0.64 g, 2.8 mL, 6.04 mmol, 4.0 equiv). (0.24 g, yield = 64%). **^{1}H NMR** (400 MHz, $CDCl_3$) δ 9.79 (s, 1H, ArCO), 7.98 (dd, *J* = 7.8, 1.5, 1H, ArH), 7.61 (td, *J* = 7.5, 1.4, 1H, ArH), 7.49 – 7.43 (m, 1H, ArH), 7.40 (td, *J* = 7.9, 1.7, 1H, ArH), 7.36 – 7.31 (m, 1H, ArH), 7.27 (dd, *J* = 7.5, 1.8, 1H, ArH), 7.10 – 7.03 (m, 1H, ArH), 6.96 (d, *J* = 8.3, 1H, ArH), 3.71 (s, 3H, OMe). **^{13}C NMR** (101 MHz, $CDCl_3$) δ 192.6, 156.5, 141.8, 134.1, 133.7, 131.4, 131.2, 130.0, 127.7, 126.9, 126.6, 121.0, 110.7, 55.4 ppm [6,7].

6-(2,4,5-Trimethoxyphenyl)benzo[*d*][1,3]dioxole-5-carbaldehyde (21)

The title compound **21** was prepared from 1-bromo-2,4,5-trimethoxybenzene (1.14 g, 4.66 mmol, 1.0 equiv), (6-formylbenzo[*d*][1,3]dioxol-5-yl)boronic acid (2.51 g, 5.59 mmol, 1.2 equiv), tetrakis(triphenylphosphine)palladium(0) (0.54 g, 0.47 mmol, 0.1 equiv) and an aqueous 2M solution of Na_2CO_3 (1.97 g, 9.2 mL, 18.6 mmol, 4.0 equiv). The product was obtained as a cream solid (1.1 g, yield = 58%). **M.p.** 89-90 °C. **FTIR:** **ṽ** = 2843.5 (C–H stretch), 1691.7 (C=O), 1604.9 (C=C), 1050.7 (C–O) cm^{-1}. **^{1}H NMR** (300 MHz, $CDCl_3$): δ = 9.61 (s, 1H, ArCO), 7.44 (s, 1H, ArH), 6.79 (d, *J* = 2.8, 2H, ArH), 6.61 (s, 1H, ArH), 6.08 (d, *J* = 3.4, 2H, Ar-CH_2), 3.97 (s, 3H, OMe), 3.87 (s, 3H, OMe), 3.73 (s, 3H, OMe). **^{13}C NMR** (101 MHz, $CDCl_3$) δ 191.1, 152.2, 151.0, 150.0, 147.5, 143.1, 139.0, 129.1, 117.5, 115.0, 110.9, 105.8, 102.0, 97.2, 56.6, 56.3, 56.2. **HRMS** (ESI+): calcd. for $C_{17}H_{17}O_6$ [M + H]+ 317.1025; found [M + H]+ 317.1013; m/z (%) = 339.0830 (23), 317.1013 (100) $[M + H]^+$, 289.1062 (18).

<u>Experimental details for the preparation of compounds 14a–14f and compound (22)</u>

2',4',5'-Trimethoxy-[1,1'-biphenyl]-2-carbaldehyde *O*-acetyl oxime (14a)

The title compound **14a** was prepared from 2',4',5'-trimethoxy-[1,1'-biphenyl]-2-carbaldehyde **13a** (0.25 g, 0.98 mmol, 1.0 equiv), hydroxylamine hydrochloride (0.13 g, 1.84 mmol, 2.0 equiv), sodium acetate (0.17 g, 1.84 mmol, 2.0 equiv), trimethylamine (0.27 g, 0.37 mL, 1.84 mmol, 2.0 equiv) and acetyl chloride (0.21 g, 1.4 mL, 1.84 mmol, 2.0 equiv). The title compound **14a** was obtained as a white solid (0.32 g, yield = 76%). **M.p.** 68-71 °C. **FTIR:** **ṽ** = 2990.5 (C–H stretch), 1610.6 (C=O), 1510.1 (C=C), 1439.8, 1348.0 (N–O stretch), 1203.8 (C–O), 755.7 cm^{-1}. **^{1}H NMR** (500 MHz,

$CDCl_3$) δ 8.15 (s, 1H, -N=CH-), 8.12 (dt, *J* = 7.9, 0.9, 1H, ArH), 7.50 (td, *J* = 7.5, 1.4, 1H, ArH), 7.4–7.36 (m, 1H, ArH), 7.32 (dd, *J* = 7.7, 1.2, 1H, ArH), 6.72 (s, 1H, ArH), 6.62 (s, 1H, ArH), 3.97 (s, 3H, OMe), 3.84 (s, 3H, OMe), 3.69 (s, 3H, OMe), 2.18 (s, 3H, CO_2Me). **^{13}C NMR** (126 MHz, $CDCl_3$) δ 169.0, 155.7, 150.7, 149.9, 143.3, 139.8, 131.2, 130.9, 128.9, 127.5, 126.4, 119.0, 115.0, 97.8, 56.6, 56.5, 56.2, 19.7. **HRMS** (ESI+): calcd. for $C_{18}H_{19}NO_5$ $[M + H]^+$ 329.1263; found $[M + H]^+$ 270.1119 (corresponding to nitrile **16a**); m/z (%) = 309.2025 (5), 288.1223 (100), 279.0925 (15), 270.1119 (25), 255.0983 (20).

2',4',6'-Trimethoxy-[1,1'-biphenyl]-2-carbaldehyde *O*-acetyl oxime (14b)

The title compound **14b** was prepared from 2',4',6'-trimethoxy-[1,1'-biphenyl]-2-carbaldehyde **13b** (0.13 g, 0.48 mmol, 1.0 equiv), hydroxylamine hydrochloride (0.19 mg, 0.96 mmol, 2.0 equiv), sodium acetate (58 mg, 0.96 mmol, 2.0 equiv), trimethylamine (97 mg, 133 μL, 0.96 mmol, 2.0 equiv) and acetyl chloride (75 mg, 69 μL, 0.96 mmol, 2.0 equiv). The product **14b** was obtained as a white solid (83 mg, yield = 53%). **M.p.** 70-72 °C. **FTIR:** **ṽ** = 2935.6 (C–H stretch), 1604.7 (C=O), 1497.6 (C=C), 1458.2, 1340.5 (N–O stretch), 1255.3 (C–O), 755.7 cm^{-1}. **^{1}H NMR** (500 MHz, $CDCl_3$) δ 8.12 (d, *J* = 7.9, 1H, ArH), 8.06 (s, 1H, -N=CH-), 7.47 (t, *J* = 7.5, 1H, ArH), 7.36 (t, *J* = 7.6, 1H, ArH), 7.27–7.23 (m, 1H, ArH), 3.88 (s, 3H, OMe), 3.68 (s, 6H, OMe), 2.17 (s, 3H, CO_2Me). **^{13}C NMR** (126 MHz, $CDCl_3$) δ 169.3, 161.6, 158.4, 155.6, 136.1, 132.3, 130.9, 129.5, 127.3, 126.1, 108.5, 90.7, 55.8, 55.4, 19.7 ppm. **HRMS** (ESI+): calcd. for $C_{18}H_{19}NO_5$ $[M + H]^+$ 329.1263; found $[M + H]^+$ 270.1120 (corresponding to nitrile **16b**); m/z (%) = 355.1501 (80), 270.1120 (100), 225.1957 (25).

2',5'-Dimethoxy-[1,1'-biphenyl]-2-carbaldehyde *O*-acetyl oxime (14c)

The title compound **14c** was prepared from 2',5'-dimethoxy-[1,1'-biphenyl]-2-carbaldehyde **13c** (0.15 g, 0.62 mmol, 1.0 equiv), hydroxylamine hydrochloride (86 mg, 1.24, 2.0 equiv mmol), sodium acetate (0.14 g, 1.23 mmol, 2.0 equiv), trimethylamine (0.17 g, 0.23 mL, 1.24 mmol, 2.0 equiv) and acetyl chloride (0.15 g, 0.12 mL, 1.234mmol, 2.0 equiv). The product **14c** was obtained as a white solid (0.14 g, yield = 79%). **M.p.** 67-70 °C. **FTIR:** $\tilde{\nu}$ = 2997.5, 2937.7 (C–H stretch), 1604.1 (C=O), 1563.9 (C=C), 1438.1, 1312.6 (N–O stretch), 1203.4 (C–O), 772.5 cm^{-1}. **^{1}H NMR** (400 MHz, $CDCl_3$) δ 8.13 (d, *J* = 5.7, 2H, -N=CH-), 7.51 (t, *J* = 7.5, 1H, ArH), 7.41 (t, *J* = 7.6, 1H, ArH), 7.31 (d, *J* = 7.6, 1H, ArH), 6.93 (d, *J* = 3.1, 2H, ArH), 6.76 (d, *J* = 2.5, 1H, ArH), 3.80 (s, 3H, OMe), 3.68 (s, 3H, OMe), 2.18 (s, 3H, CO_2Me). **^{13}C NMR** (101 MHz, $CDCl_3$) δ 169.1, 155.4, 153.7, 150.6, 139.8, 131.2, 130.7, 128.7, 127.8, 126.4, 117.2, 114.4, 112.3, 56.1, 55.8, 19.7 ppm. **HRMS** (ESI^+): calcd. for $C_{17}H_{17}NO_4Na$ $[M + Na]^+$ 322.1055; found $[M + Na]^+$ 322.1048; *m/z* (%) = 323.1076 (20), 322.1048 (100) $[M + Na]^+$, 317.1499 (18) 300.1233 (20).

2',3'-Dimethoxy-[1,1'-biphenyl]-2-carbaldehyde *O*-acetyl oxime (14d)

The title compound **14d** was prepared from 2',3'-dimethoxy-[1,1'-biphenyl]-2-carbaldehyde **13d** (0.17 g, 0.63 mmol, 2.0 equiv), hydroxylamine hydrochloride (0.88 mg, 1.27 mmol, 2.0 equiv), sodium acetate (0.12 g, 1.27 mmol, 2.0 equiv), trimethylamine (0.13 g, 0.18 mL, 1.27 mmol, 2.0 equiv) and acetyl chloride (0.20 g, 1.3 mL, 2.54 mmol, 2.0 equiv). The title compound **14d** was obtained as white solid (0.16 g, yield = 76%). **M.p.** 64-66 °C. **FTIR:** $\tilde{\nu}$ = 2943.7 (C–H stretch), 1742.0 (C=O), 1531.7 (C=C), 1438.1, 1329.2 (N–O stretch), 1281.6 (C–O) cm^{-1}. **^{1}H NMR** (400 MHz, $CDCl_3$) δ 8.21 (s, 1H, -N=CH-), 8.17 (d, *J* = 7.9, 1H, ArH), 7.55–7.47 (m, 1H, ArH), 7.45–7.38

(m, 1H, ArH), 7.34 (dd, *J* = 7.7, 1.3, 1H, ArH), 7.14 (t, *J* = 7.9, 1H, ArH), 7.00 (dd, *J* = 8.3, 1.5, 1H, ArH), 6.79 (dd, *J* = 7.6, 1.5, 1H, ArH), 3.92 (s, 3H, OMe), 3.48 (s, 3H, OMe), 2.15 (s, 3H, CO_2Me). **^{13}C NMR** (101 MHz, $CDCl_3$) δ 168.8, 155.2, 152.9, 146.3, 139.6, 133.2, 131.0, 130.7, 128.5, 127.8, 126.5, 124.2, 123.2, 112.4, 60.5, 55.9, 19.6 ppm. **HRMS** (ESI$^+$): calcd. for $C_{17}H_{17}NO_4Na$ [M + Na]$^+$ 322.1055; found [M + Na]$^+$ 322.1161; *m/z* (%) = 322.1161 (20) [M + Na]$^+$, 300.1338 (5), 272.1378 (25), 258.1216 (100), 240.1106 (95), 225.0989 (10).

(*E*/*Z*)-2',4'-Dimethoxy-[1,1'-biphenyl]-2-carbaldehyde O-acetyl oxime (14e)

The title compound **14e** was prepared from 2',4'-dimethoxy-[1,1'-biphenyl]-2-carbaldehyde **13e** (0.40 g, 1.72 mmol, 1.0 equiv), hydroxylamine hydrochloride (0.24 g, 3.44 mmol, 2.0 equiv), sodium acetate (0.28 g, 3.41 mmol, 2.0 equiv), trimethylamine (0.34 g, 0.47 mL, 3.41 mmol, 2.0 equiv) and acetyl chloride (0.27 g, 0.24 mL, 3.41 mmol, 2.0 equiv). The product **14e** was furnished as a white solid (0.33 g, yield = 65%). **M.p.** 74-76 °C. **FTIR:** **ṽ** = 2951.1 (C–H stretch), 1778.8 (C=O), 1581.3 (C=C), 1466.5, 1365.0 (N–O stretch), 1270.1 (C–O), 774.1 cm^{-1}. **^{1}H NMR** (500 MHz, $CDCl_3$) δ 8.14 (s, 1H, -N=C-), 8.11 (dd, *J* = 7.9, 1.3, 1H, ArH), 7.45 (td, *J* = 7.6, 1.4, 1H, ArH), 7.34 (td, *J* = 7.6, 1.1, 1H, ArH), 7.27 (dd, *J* = 7.6, 1.3, 1H,ArH), 7.06 (d, *J* = 8.1, 1H, ArH), 6.57 (d, *J* = 2.4, 1H, ArH), 6.55 (d, *J* = 2.4, 1H, ArH), 3.83 (s, 3H, OMe), 3.69 (s, 3H, OMe), 2.14 (s, 3H, CO_2Me) (major isomer), 7.94 (s, 1H, -N=C-), 7.89 (dd, *J* = 7.9, 1H, ArH), 7.30 (dd, *J* = 7.9, 1H, ArH), 7.23 (dd, *J* = 7.6, 1.4, 1H, ArH), 7.04 (d, *J* = 1.4, 1H, ArH), 3.82 (s, 3H, OMe) (minor isomer).**^{13}C NMR** (126 MHz, $CDCl_3$) δ 169.1, 161.2, 157.4, 155.8, 140.0, 132.1, 131.2, 131.2, 128.9, 126.4, 120.4, 104.8, 98.7, 55.5, 19.7 ppm (major isomer), 160.9, 157.5, 149.6, 138.5, 132.0, 131.0, 129.4, 127.3, 125.2, 121.1, 104.7, 98.7, 55.4 (minor isomer). **HRMS** (ESI$^+$): calcd. for

$C_{17}H_{17}NO_4Na$ [M + Na]$^+$ 322.1055; found [M + Na]$^+$ 322.1041; *m/z* (%) = 291.2824 (7), 338.0771 (7), 322.1041 (100) [M + Na]$^+$, 309.2041 (5).

(*E*/*Z*)-2'-Methoxy-[1,1'-biphenyl]-2-carbaldehyde O-acetyl oxime (14f)

The title compound **14f** was prepared from 2'-methoxy-[1,1'-biphenyl]-2-carbaldehyde **13f** (0.19 g, 0.85 mmol, 1.0 equiv), hydroxylamine hydrochloride (0.12 g, 1.72 mmol, 2.0 equiv), sodium acetate (0.14 g, 1.72 mmol, 2.0 equiv), trimethylamine (0.17 g, 0.25 mL, 1.72 mmol, 2.0 equiv) and acetyl chloride (0.15 g, 0.18 mL, 1.72 mmol, 2.0 equiv). The product **14f** was obtained as a tan solid (0.14 g, yield = 62%). **M.p.** 92-93 °C. **FTIR:** $\tilde{\nu}$ = 2939.3 (C–H stretch), 1756.5 (C=O), 1595.6 (C=C), 1430.1, 1364.3 (N–O stretch), 1256.5 (C–O), 746.2 cm^{-1}. **^{1}H NMR** (500 MHz, $CDCl_3$) δ 8.12 (s, 1H, ArH), 7.49 (td, *J* = 7.6, 1.4, 1H, ArH), 7.42–7.36 (m, 3H, ArH), 7.30 (dd, *J* = 7.6, 1.3, 1H, ArH), 7.17 (dd, *J* = 7.5, 1.8, 1H, ArH), 7.04 (td, *J* = 7.5, 1.0, 1H, ArH), 6.99–6.96 (m, 1H, ArH), 3.73 (s, 3H, OMe), 2.16 (s, 3H, CO_2Me) (major isomer), 7.91 (s, 1H, -N=C-), 7.90 (d, *J* = 1.4, 1H, ArH), 7.71 (dd, *J* = 7.7, 1.3, 1H, ArH), 7.60 (td, *J* = 7.7, 1.4, 1H, ArH), 7.44 (dd, *J* = 7.7, 1.1, 1H, ArH), 7.36 – 7.33 (m, 1H, ArH), 7.26 (dd, *J* = 7.8, 1.6, 1H, ArH), 7.01 (dd, *J* = 3.8, 0.9, 1H, ArH), 6.95 (d, *J* = 1.0, 1H, ArH), 3.82 (s, 3H, OMe). **^{13}C NMR** (126 MHz, $CDCl_3$) δ 169.1, 156.3, 155.5, 140.0, 131.6, 131.2, 130.9, 129.8, 128.7, 127.8, 127.7, 126.4, 120.9, 111.0, 55.5, 19.7 ppm (major isomer), 156.5, 156.5, 149.7, 142.6, 138.7, 132.8, 132.4, 131.5, 130.8, 130.7, 130.4, 129.5, 129.4, 128.4, 127.4, 127.3, 125.1, 120.8, 120.7, 111.3, 111.0, 55.5 (minor isomer). **HRMS** (ESI$^+$): calcd. for $C_{16}H_{15}NNaO_3$ [M + Na]$^+$ 292.0950; found [M + Na]$^+$ 292.0955; *m/z* (%) = 302.0176 (20), 292.0955 (100) [M + Na]$^+$, 209.0842 (80).

6-(2,4,5-trimethoxyphenyl)benzo[*d*][1,3]dioxole-5-carbaldehyde *O*-acetyl oxime (22)

The title compound **22** was prepared from 6-(2,4,5-trimethoxyphenyl)benzo[*d*][1,3]dioxole-5-carbaldehyde **21** (0.97 g, 3.07 mmol, 1.0 equiv), hydroxylamine hydrochloride (0.43 g, 6.14 mmol, 2.0 equiv), sodium acetate (0.52 g, 2.57 mmol), trimethylamine (0.62 g, 0.85 mL, 6.13 mmol, 2.0 equiv) and acetyl chloride (0.48 g, 0.44 mL, 6.13 mmol, 2.0 equiv). The product **22** was furnished as a white solid (0.84 g, yield = 74%). **M.p.** 68-71 °C. **FTIR:** ṽ = 2913.7 (C–H stretch), 1681.3 (C=O), 1519.6 (C=C), 1398.0 (N–O stretch), 1254.6 (C–O), 719.0 cm^{-1}. **^{1}H NMR** (400 MHz, $CDCl_3$) δ 8.02 (s, -N=CH-), 7.59 (s, 1H, ArH), 6.76 (s, 1H, ArH), 6.66 (s, 1H, ArH), 6.61 (s, 1H, ArH), 6.04 (d, *J* = 2.3, 2H, ArH), 3.97 (s, 3H, OMe), 3.84 (s, 3H, OMe), 3.72 (s, 3H, OMe), 2.17 (s, 3H, CO_2Me). **^{13}C NMR** (101 MHz, $CDCl_3$) δ 169.1, 155.3, 150.7, 150.3, 149.8, 147.4, 143.1, 135.5, 122.5, 118.6, 115.0, 110.7, 105.6, 101.8, 97.7, 56.6, 56.5, 56.2, 19.7. **HRMS** (ESI+): calcd. for $C_{19}H_{19}NO_7$ $[M + H]^+$ 374.1162; found $[M + H]^+$ 374.1236; m/z (%) = 396.1045 (30) $[M + Na]^+$, 374.1236 (15) $[M + H]^+$, 314.1018 (100).

Experimental details for the preparation of compounds 15a–15d, 16a–16f and compounds 23 and 24

2,3-Dimethoxyphenanthridine 15a and **2',4',5'-Trimethoxy-[1,1'-biphenyl]-2-carbonitrile (16a)**

UV irradiation of **14a** (93 mg, 0.28 mmol, 1.0 equiv) afforded the desired product **15a** was obtained as an oil (50.1 mg, yield = 74%) after column chromatography (10 to 30% EtOAc/hexane). ^{1}H NMR (300 MHz, $CDCl_3$) δ = 9.46–9.31 (m, 1H, ArH), 9.22 (s, 1H, ArH), 8.08–7.97 (m, 1H, ArH), 7.81 (ddd, *J* = 8.6, 7.0, 1.6, 1H, ArH), 7.62 (ddd, *J*

= 8.0, 7.0, 1.1, 1H, ArH), 7.29 (d, *J* = 2.5, 1H, ArH), 6.81 (d, *J* = 2.5, 1H, ArH), 4.12 (s, 3H, OMe), 3.99 (s, 3H) [8]. In addition, the nitrile **16a** (11.2 mg, yield = 12%) was obtained. **M.p.** 143-144 °C. **FTIR:** **ṽ** = 2936.0 (C–H stretch), 2225.4 (C≡N), 1499.1 (C=C), 1458.2, 1051.0 (C–O), 831.6 cm^{-1}. **^{1}H NMR** (300 MHz, $CDCl_3$) δ 7.88 (d, *J* = 0.6, 1H, ArH), 7.82 (d, *J* = 8.4, 1H, ArH), 7.32 (ddd, *J* = 8.4, 2.3, 0.6, 1H, ArH), 7.29–7.27 (m, 1H, ArH), 6.69 (s, 1H, ArH), 6.60 (s, 1H, ArH), 3.96 (s, 3H, OMe), 3.85 (s, 3H, OMe), 3.73 (s, 3H, OMe). **^{13}C NMR** (75 MHz, $CDCl_3$) δ 151.0, 150.3, 149.6, 143.5, 140.4, 135.7, 131.1, 129.7, 128.0, 126.9, 118.4, 115.0, 97.9, 57.0, 56.8, 56.6. **HRMS** (ESI+): calcd. for $C_{16}H_{16}NO_3$ $[M + H]^+$ 270.1130; found $[M + H]^+$ 270.1225; m/z (%) = 288.1337 (47), 279.1037 (100), 270.1225 (52) $[M + H]^+$, 257.1174 (20), 258.1225 (27), 256.1062 (25).

1,3-Dimethoxyphenanthridine 15b and **2',4',6'-trimethoxy-[1,1'-biphenyl]-2-carbonitrile (16b)**

UV irradiation of **14b** (77 mg, 0.23 mmol, 1.0 equiv) afforded the desired product **15b** as an oil (16.1 mg, yield = 28%) after column chromatography (5% EtOAc/hexane). **^{1}H NMR** (300 MHz, $CDCl_3$) δ = 9.46–9.31 (m, 1H, ArH), 9.22 (s, 1H, ArH), 8.08–7.97 (m, 1H, ArH), 7.81 (ddd, *J* = 8.6, 7.0, 1.6, 1H, ArH), 7.62 (ddd, *J* = 8.0, 7.0, 1.1, 1H, ArH), 7.29 (d, *J* = 2.5, 1H, ArH), 6.81 (d, *J* = 2.5, 1H, ArH), 4.12 (s, 3H, OMe), 3.99 (s, 3H, OMe). In addition, the nitrile **16b** (33.8 mg, yield = 47%) was also formed as a liquid. **^{1}H NMR** (300 MHz, $CDCl_3$) δ 7.7 –7.71 (m, 1H, ArH), 7.62 (td, *J* = 7.7, 1.4, 1H, ArH), 7.44 (dt, *J* = 7.7, 1.1, 1H, ArH), 7.39 (dd, *J* = 7.6, 1.3, 1H, ArH), 6.28 (s, 2H, $ArOCH_2$-), 3.90 (s, 3H, OMe), 3.79 (s, 6H, OMe). **^{13}C NMR** (75 MHz, $CDCl_3$) δ 162.3, 158.7, 139.2, 132.9, 132.8, 132.2, 127.2, 119.3, 115.1, 109.0, 91.3, 56.1, 55.7 ppm [9,10].

2-Methoxyphenanthridine 15c and **2',5'-Dimethoxy-[1,1'-biphenyl]-2-carbonitrile (16c)**

UV irradiation of **14c** (95 mg, 0.32 mmol, 1.0 equiv) furnished the desired product as an oil **15c** (38.5 mg, yield = 58%) after column chromatography (gradient from 5% to 30% EtOAc/hexane).**^{1}H NMR** (300 MHz, $CDCl_3$) δ 9.17 (s, 1H, ArH), 8.55 (d, *J* = 8.3, 1H, ArH), 8.12 (d, *J* = 9.0, 1H, ArH), 8.08–7.98 (m, 1H, ArH), 7.91 (d, *J* = 2.8, 1H, ArH), 7.85 (ddd, *J* = 8.4, 7.0, 1.4, 1H, ArH), 7.71 (ddd, *J* = 8.0, 7.0, 1.1, 1H, ArH), 7.38 (dd, *J* = 9.0, 2.8, 1H, ArH), 4.03 (s, 3H, OMe). **^{13}C NMR** (75 MHz, $CDCl_3$) δ 158.5, 151.1, 132.1, 131.4, 130.6, 128.8, 127.6, 126.5, 125.2, 121.9, 118.6, 103.1, 55.7 ppm [11]. The nitrile **16c** was furnished as a white solid (13.0 mg, yield = 17%). **^{1}H NMR** (400 MHz, $CDCl_3$) δ 7.71 (dd, *J* = 7.7, 1.3, 1H, ArH), 7.61 (td, *J* = 7.7, 1.3, 1H, ArH), 7.45 (d, *J* = 7.8, 1H, ArH), 7.43–7.37 (m, 1H, ArH), 6.94 (d, *J* = 1.7, 2H, ArH), 6.83 (t, *J* = 1.6, 1H, ArH), 3.79 (s, 3H, OMe), 3.78 (s, 3H, OMe). **^{13}C NMR** (101 MHz, $CDCl_3$) δ 153.6, 150.7, 142.4, 132.8, 132.4, 130.8, 128.0, 127.5, 118.6, 116.7, 115.0, 113.4, 112.5, 56.0, 55.9 ppm [12].

4-Methoxyphenanthridine 15d and **2',3'-Dimethoxy-[1,1'-biphenyl]-2-carbonitrile (16d)**

UV irradiation of **14d** (97 mg, 0.32 mmol, 1.0 equiv) afforded the desired product **15d** (36.2 mg, yield = 54%) after column chromatography (10%% EtOAc/hexane). **^{1}H NMR** (400 MHz, $CDCl_3$) δ 9.17 (s, 1H, ArH), 8.55 (d, *J* = 8.3, 1H, ArH), 8.12 (d, *J* = 9.0, 1H, ArH), 8.08–7.98 (m, 1H, ArH), 7.91 (d, *J* = 2.8, 1H, ArH), 7.85 (ddd, *J* = 8.4, 7.0, 1.4, 1H, ArH), 7.71 (ddd, *J* = 8.0, 7.0, 1.1, 1H, ArH), 7.38 (dd, *J* = 9.0, 2.8, 1H, ArH), 4.03 (s, 3H, OMe). **^{13}C NMR** (101 MHz, $CDCl_3$) δ 156.4, 149.0, 137.6, 130.0, 129.4, 128.5, 126.7, 125.5, 124.4, 123.0, 119.8, 116.5, 101.0, 53.6 [13,14]. In addition, the nitrile

16d (16.8 mg, yield = 22%) was obtained. **M.p.** 154-155 °C. **FTIR:** $\tilde{\nu}$ = 2937.0 (C–H stretch), 2217.5 (C≡N), 1586.8 (C=C), 1472.0, 1025.9 (C–O), 846.8 cm^{-1}. **^{1}H NMR** (400 MHz, $CDCl_3$) δ 7.75 (d, *J* = 7.8, 1H, ArH), 7.62 (t, *J* = 7.7, 1H, ArH), 7.49 (d, *J* = 7.9, 1H, ArH), 7.45 (t, *J* = 7.7, 1H, ArH), 7.15 (t, *J* = 7.9, 1H, ArH), 7.01 (d, *J* = 8.2, 1H, ArH), 6.91 (dd, *J* = 7.6, 1.6, 1H, ArH), 3.92 (s, 3H, OMe), 3.65 (s, 3H, OMe). **^{13}C NMR** (101 MHz, $CDCl_3$) δ 152.3, 145.9, 141.7, 132.2, 131.9, 131.5, 130.4, 126.9, 123.4, 121.9, 117.8, 112.6, 112.2, 60.3, 55.3. **HRMS** (ESI+): calcd. for $C_{15}H_{14}NO_2$ $[M + H]^+$ 240.1025; found $[M + H]^+$ 240.1018; m/z (%) = 258.112 (10), 240.1018 (100) $[M + H]^+$, 210.0906 (7).

2',4'-Dimethoxy-[1,1'-biphenyl]-2-carbonitrile (16e)

UV irradiation of **14e** (90 mg, 0.33 mmol) afforded a crude product which was purified by column chromatography (20% EtOAc/hexane as eluent). The nitrile **16e** was obtained (67.1 mg, yield = 81%) as light yellow solid. **M.p.** 166-167 °C. **^{1}H NMR** (300 MHz, $CDCl_3$) δ 7.74 (ddd, *J* = 7.7, 1.4, 0.6, 1H, ArH), 7.63 (td, *J* = 7.7, 1.4, 1H, ArH), 7.50–7.45 (m, 1H, ArH), 7.42 (td, *J* = 7.6, 1.3, 1H, ArH), 6.66–6.59 (m, 2H, ArH), 3.90 (s, 3H, OMe), 3.86 (s, 3H, OMe). **^{13}C NMR** (75 MHz, $CDCl_3$) δ 161.9, 157.9, 142.8, 133.1, 132.7, 131.9, 131.4, 127.3, 120.4, 119.2, 113.8, 105.2, 99.3, 55.8 ppm. **HRMS** (ESI^+): calcd. for $C_{15}H_{14}NO_2$ $[M + H]^+$ 240.1025; found $[M + H]^+$ 240.1018; *m/z* (%) = 258.1126 (10), 240.1018 (15) $[M + H]^+$, 210.0917 (100).

2'-Methoxy-[1,1'-biphenyl]-2-carbonitrile (16f)

UV irradiation of **14f** (89 mg, 0.33 mmol) afforded the nitrile (62.8 mg, yield = 69%) as a clear oil after column chromatography using 20% EtOAc/hexane as eluent. **M.p.** 154-156 °C. **FTIR:** $\tilde{\nu}$ = 2924.9 (C–H stretch), 2221.4 (C≡N), 1502.0 (C=C), 1467.5, 1066.7 (C–O), 821.5 cm^{-1}. **^{1}H NMR** (400 MHz, $CDCl_3$) δ 7.70 (d, *J* = 7.7, 1H, ArH), 7.59 (td, *J*

= 7.7, 1.5, 1H, ArH), 7.44 (d, *J* = 7.8, 1H, ArH), 7.39 (td, *J* = 7.7, 2.5, 2H, ArH), 7.24 (dd, *J* = 7.5, 1.7, 1H, ArH), 7.05 (d, *J* = 7.5, 1H, ArH), 7.01 (d, *J* = 8.4, 1H, ArH), 3.82 (s, 3H, OMe). **^{13}C NMR** (101 MHz, $CDCl_3$) δ 156.5, 142.6, 132.8, 132.4, 130.9, 130.9, 130.4, 127.3, 120.8, 118.7, 113.5, 111.4, 55.5 ppm. **HRMS** (ESI+): calcd. for $C_{14}H_{12}NO$ $[M + H]^+$ 210.0919; found $[M + H]^+$ 210.0921; m/z (%) = 238.0982 (20), 264.0842 (50), 210.0921 (100) $[M + H]^+$ [15].

2,3-Dimethoxy-[1,3]dioxolo[4,5-*j*]phenanthridine (23) and 6-(2,4,5-Trimethoxyphenyl)benzo[*d*][1,3]dioxole-5-carbonitrile (24)

UV irradiation of **22** (95 mg, 0.32 mmol, 1.0 equiv) formed phenanthridine **23** as cream solid after column chromatography using 50% EtOAc/hexane as eluent (37.2 mg, yield = 41%). **^{1}H NMR** (400 MHz, Methanol-d_4) δ 8.88 (s, 1H, ArH), 7.97 (s, 1H, ArH), 7.74 (s, 1H, ArH), 7.36 (s, 1H, ArH), 7.28 (s, 1H, ArH) 6.14 (s, 2H, ArH), 3.97 (s, 3H, OMe), 3.93 (s, 3H, OMe). **^{13}C NMR** (101 MHz, Methanol-d_4) δ 153.6, 151.7, 150.4, 148.5, 146.9, 131.6, 127.9, 126.6, 124.2, 105.4, 105.1, 102.6, 99.5, 55.4, 55.2. In addition, nitrile **24** (53.3 mg, yield = 53%)-was also obtained as a white solid. **M.p.** 128-129 °C. FTIR: **ṽ** = 2840.7 (C–H stretch), 2211.8 (C≡N), 1483.2 (C=C), 1438.1, 1028.3 (C–O) cm^{-1}). **^{1}H NMR** (400 MHz, $CDCl_3$) δ 7.09 (s, 1H, ArH), 6.89 (s, 1H, ArH), 6.79 (s, 1H, ArH), 6.62 (s, 1H, ArH), 6.08 (s, 2H, ArH), 3.95 (s, 3H, OMe), 3.86 (s, 3H, OMe), 3.82 (s, 3H, OMe). **^{13}C NMR** (101 MHz, $CDCl_3$) δ 151.1, 150.9, 150.3, 146.7, 143.0, 138.8, 118.9, 118.1, 114.5, 111.6, 111.4, 105.6, 102.3, 97.6, 56.7, 56.3, 56.1. **HRMS** (ESI+): calcd. for $C_{17}H_{16}NO_5$ $[M + H]^+$ 314.1028; found [M + H]+ 314.1023; m/z (%) = 383.1098 (10), 336.0842 (30), 314.1023 (100) [M + H]+, 102.1279 (80).

References

1. Mori, I.; Nakachi, Y.; Ueda, K.; Uemura, D.; Hirata, Y. *Tetrahedron Lett.* **1978**, *26*, 2297-2298. DOI: 10.1016/S0040-4039(01)91518-7.

2. Irngartinger, H.; Escher, T. *Supramolecular Chemistry*, **2001**, *13*, 207-232. DOI: 10.1080/10610270108034895

3. Pradeep, P.; Ngwira, K. J.; Reynolds, C.; Rousseau, A. L.; Lemmerer, A.; Fernandes, M. A.; Johnson, M. M.; de Koning, C. B. *Tetrahedron*, **2016**, *72*, 8417-8427. DOI: 10.1016/j.tet.2016.10.071.

4. Tummatorn, J.; Krajangsri, S.; Norseeda, K.; Thongsornkleeba, C.; Ruchirawata, S. *Org. Biomol. Chem.* **2014**, *12*, 5077–5081. DOI: 10.1039/C4OB00797B

5. Schaarschmidt, M.; Höfner, G.; Wanner, K. T. *ChemMedChem* **2019**, *14*, 1135-1151. DOI: 10.1002/cmdc.201900170.

6. Sun, N.; Yang, H.; Zheng, K.; Jin, L.; Hu, B.; Shen, Z.; Hu, X. *Eur. J. Org. Chem.* **2020**, 6135-1488. DOI: 10.1002/ejoc.202001059.

7. Wang, Y.; Fang, Z.; Chen, X.; Wang, Y. *Chem. Eur. J*. **2020**, *26*, 6805-6811. DOI: 10.1002/chem.201905855.

8. Chen, W.-L.; Chen, C.-Y.; Chen, Y.-F.; Hsieh, J.-C. *Org. Lett*. **2015**, *17*, 1613−1616. DOI: 10.1021/acs.orglett.5b00544.

9. Zhou, Y.; Deng, S.; Mai, S.; Song, Q. *Org. Lett*. **2018**, *20*, 6161-6165. DOI: 10.1021/acs.orglett.8b02629.

10. Bardagi, J. I.; Ghosh, I.; Schmalzbauer, M.; Ghosh, T.; König, B. *Eur. J. Org. Chem*. **2018**, 34–40. DOI: 10.1002/ejoc.201701461.

11. Gao, Y.; Jing, Y.; Li, L.; Zhang, J.; Chen, X.; Ma, Y.-N. *J. Org. Chem.* **2020**, *85*, 12187−12198. DOI: 10.1021/acs.joc.0c01390.

12. Urawa, Y.; Naka, H.; Miyazawa, M.; Souda, S.; Ogura, K. *J. Organomet. Chem*. **2002**, *653*, 269–278.

13. Jiang, H.; An, X.; Tong, K.; Zheng, T.; Zhang, Y.; Yu, S. *Angew. Chem. Int. Ed.* **2015**, *54*, 4055 –4059. DOI: 10.1002/anie.201411342.

14. Linsenmeier, A. M.; Williams, C. M.; Bräse, S. *J. Org. Chem*. **2011**, *76*, 9127–9132. DOI: 10.1021/jo201542x.

15. Kang, K.; Huang, L.; Weix, D. J. *J. Am. Chem. Soc*. **2020**, *142*, 10634−10640. DOI: 10.1021/jacs.0c04670.

2',4',5'-Trimethoxy-[1,1'-biphenyl]-2-carbaldehyde (13a)

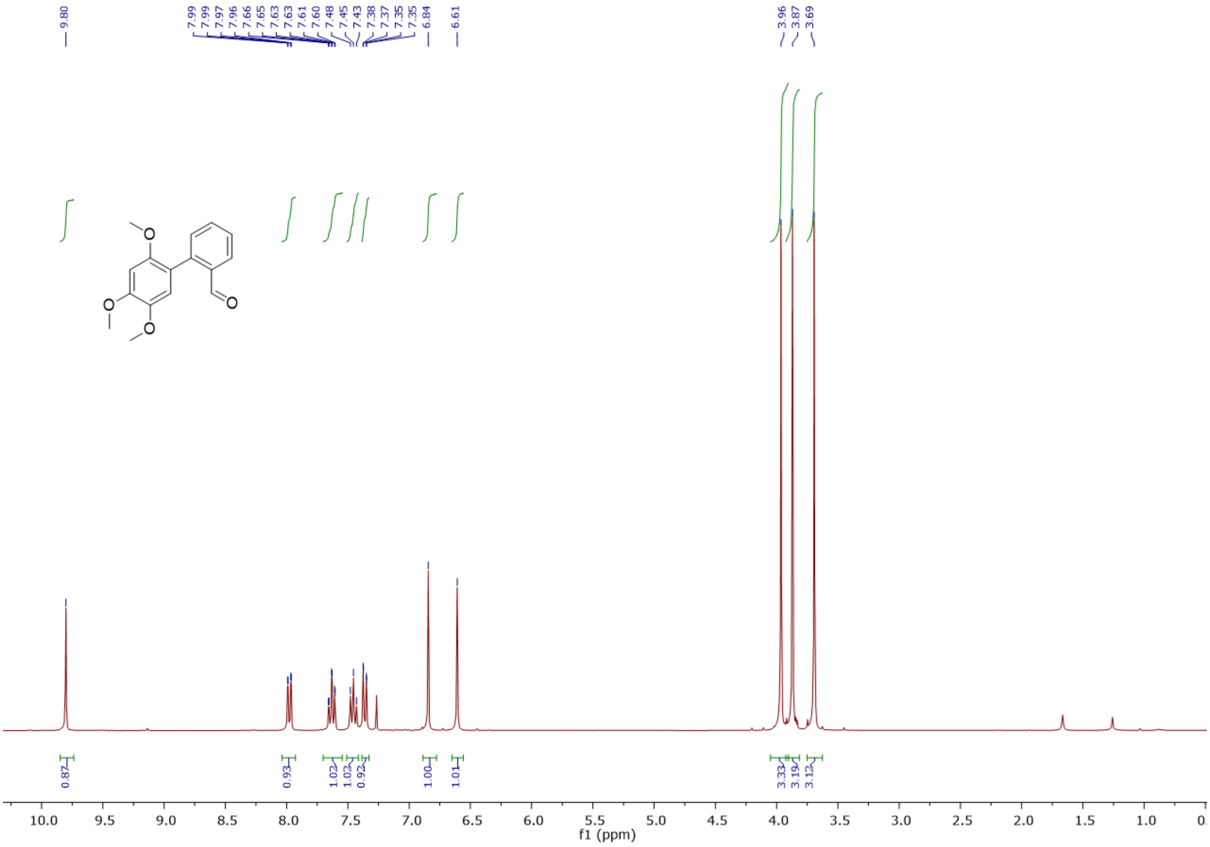

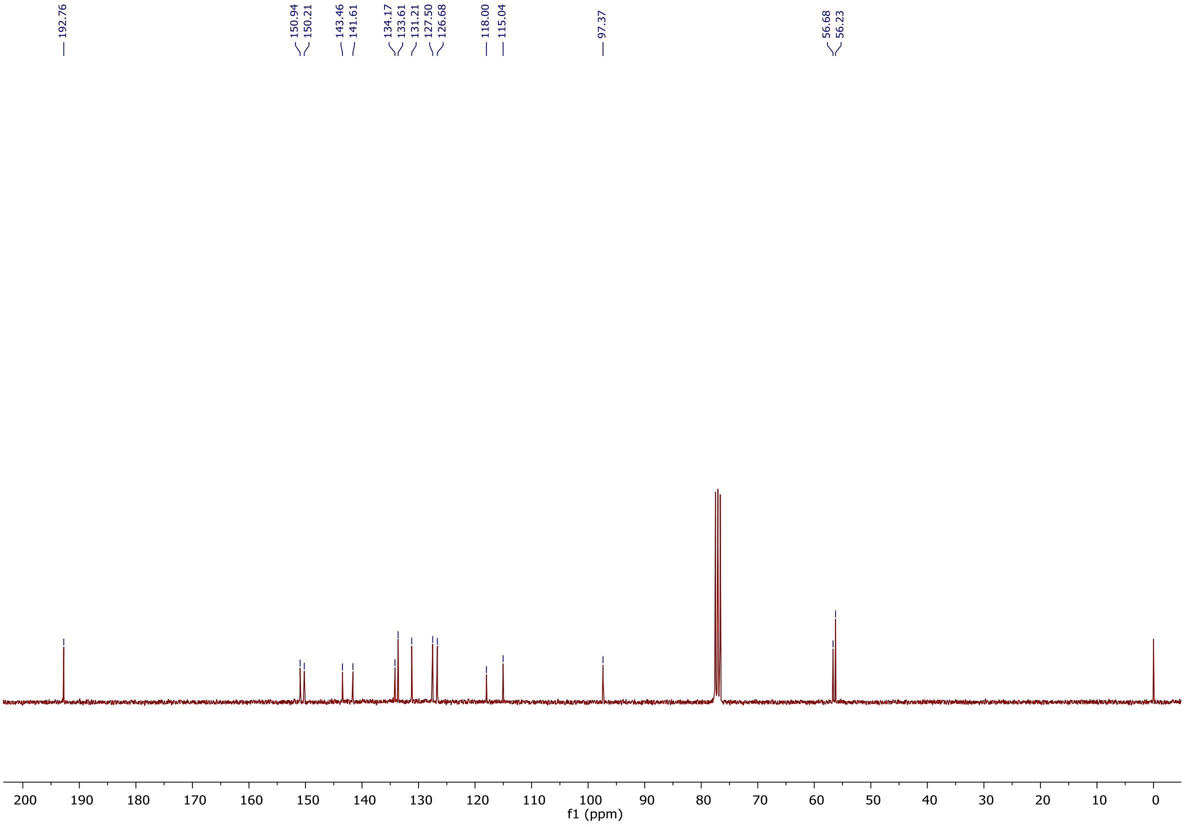

2',4',6'-Trimethoxy-[1,1'-biphenyl]-2-carbaldehyde (13b)

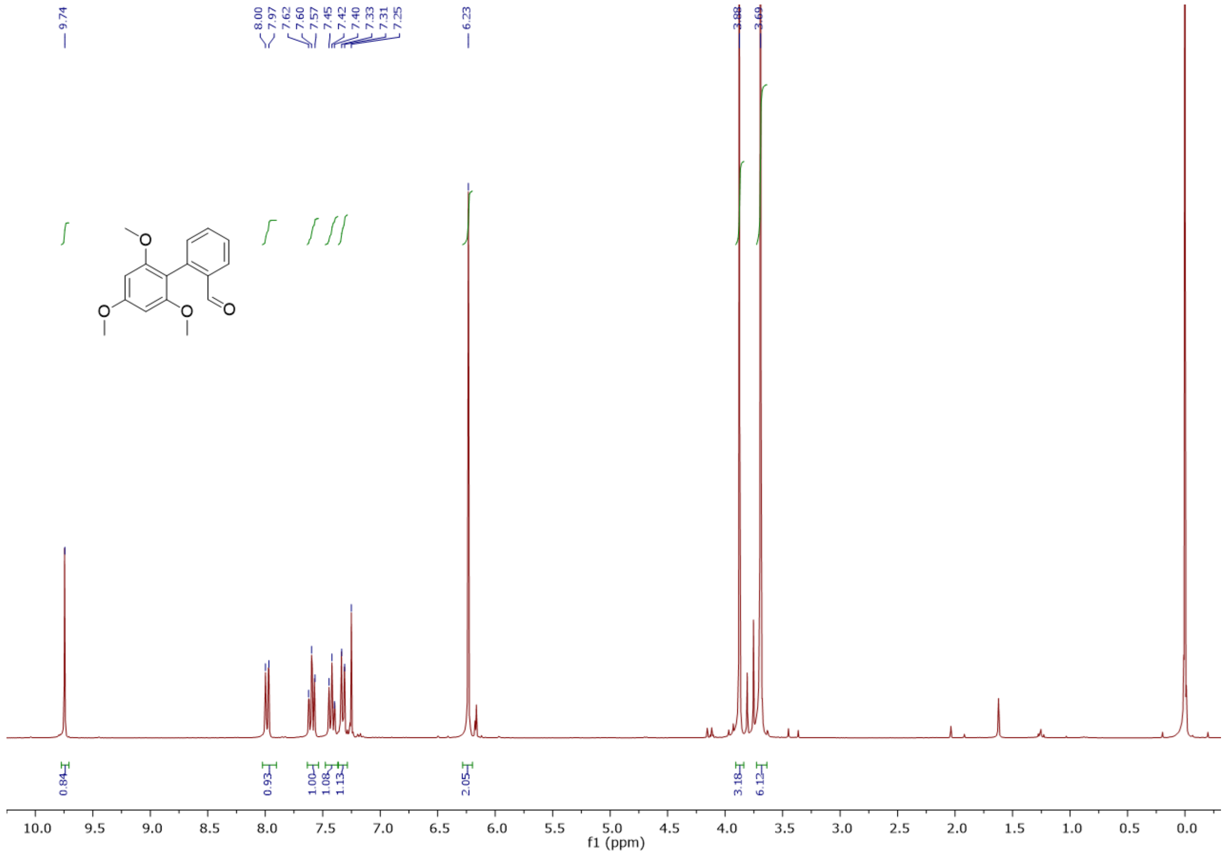

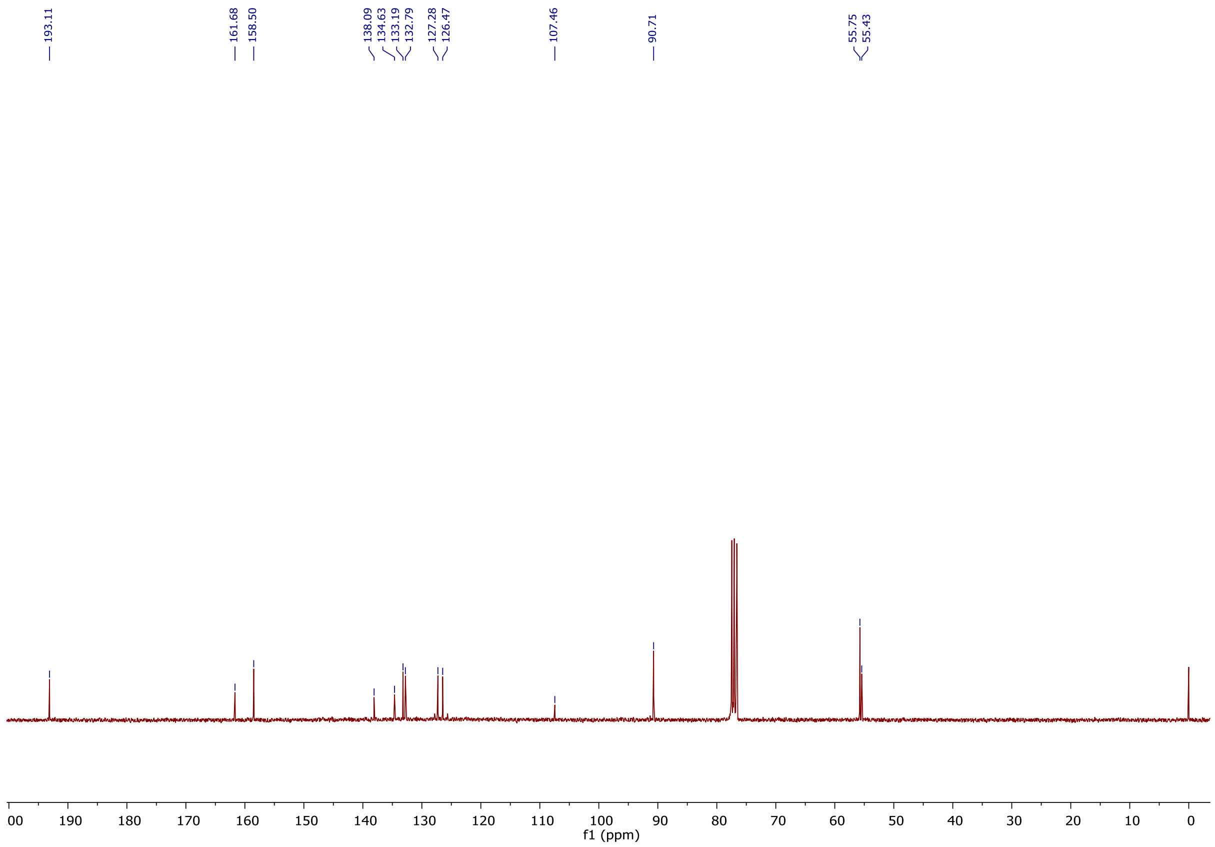

2',5'-Dimethoxy-[1,1'-biphenyl]-2-carbaldehyde (13c)

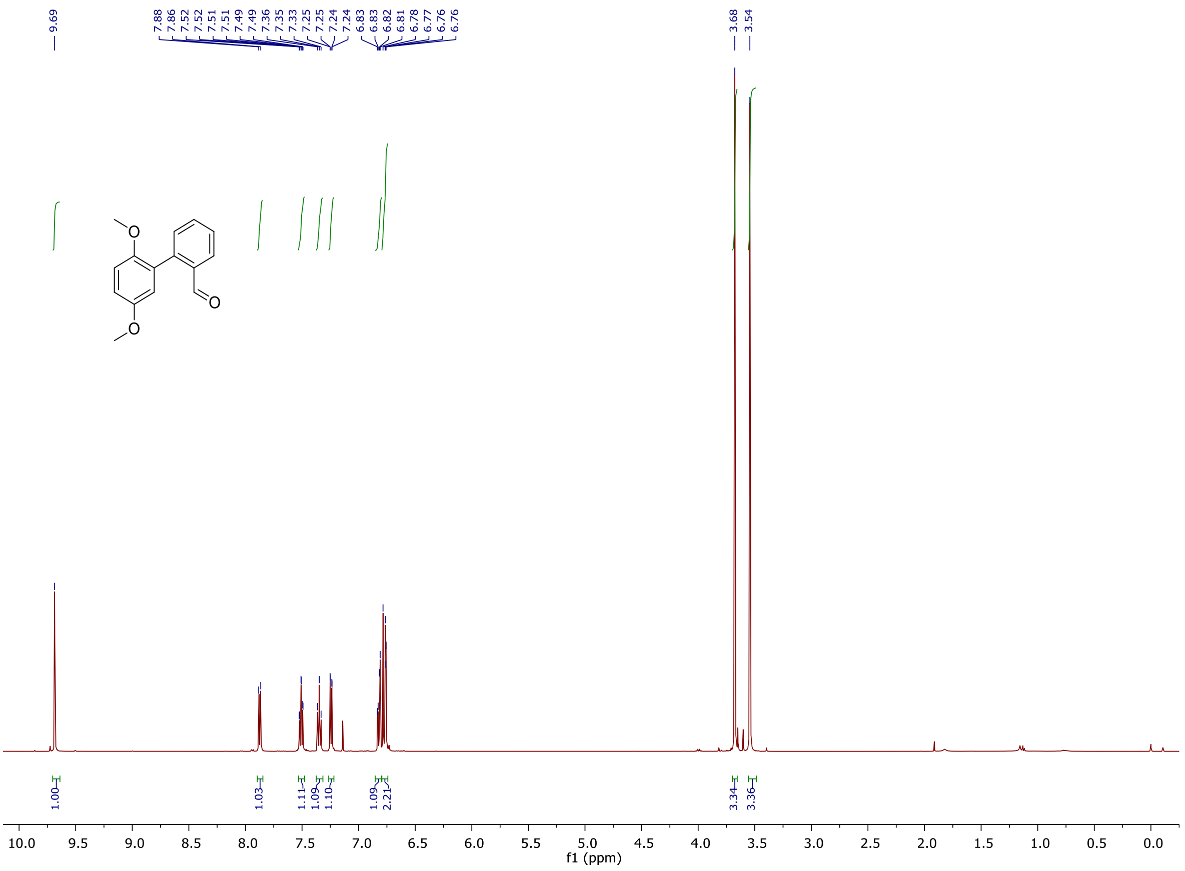

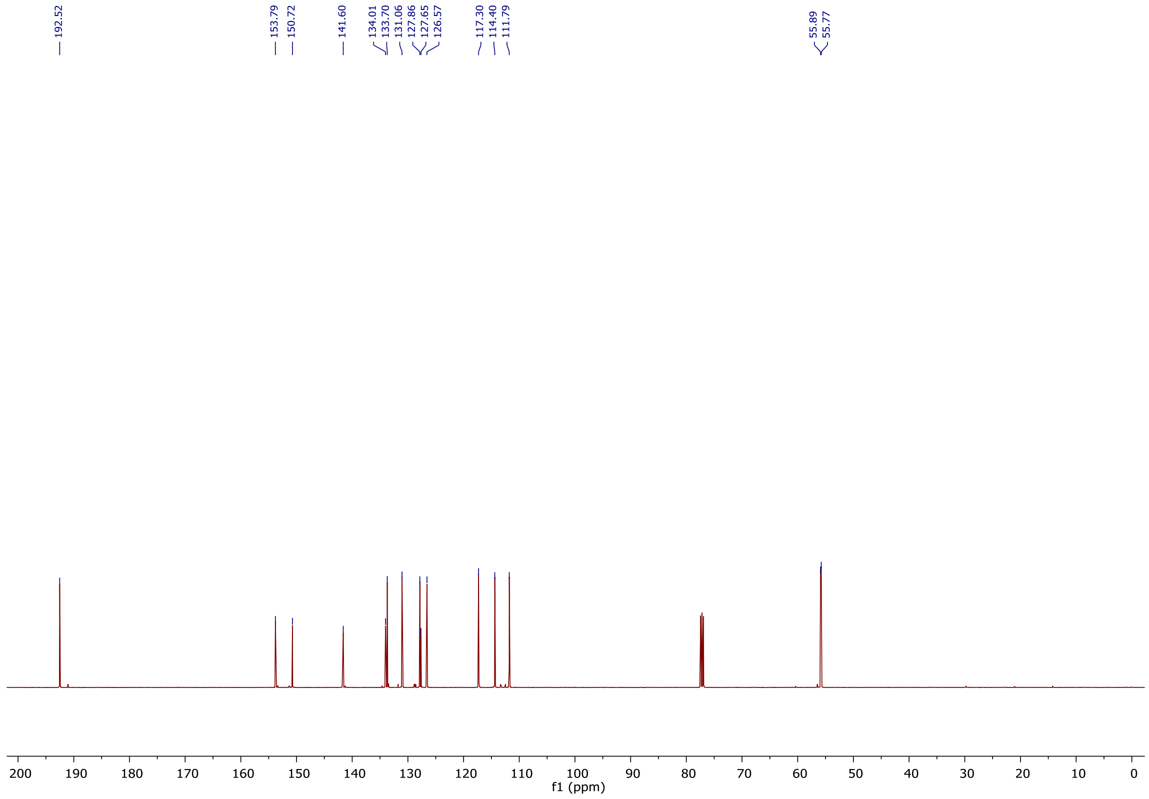

2',3'-Dimethoxy-[1,1'-biphenyl]-2-carbaldehyde (13d)

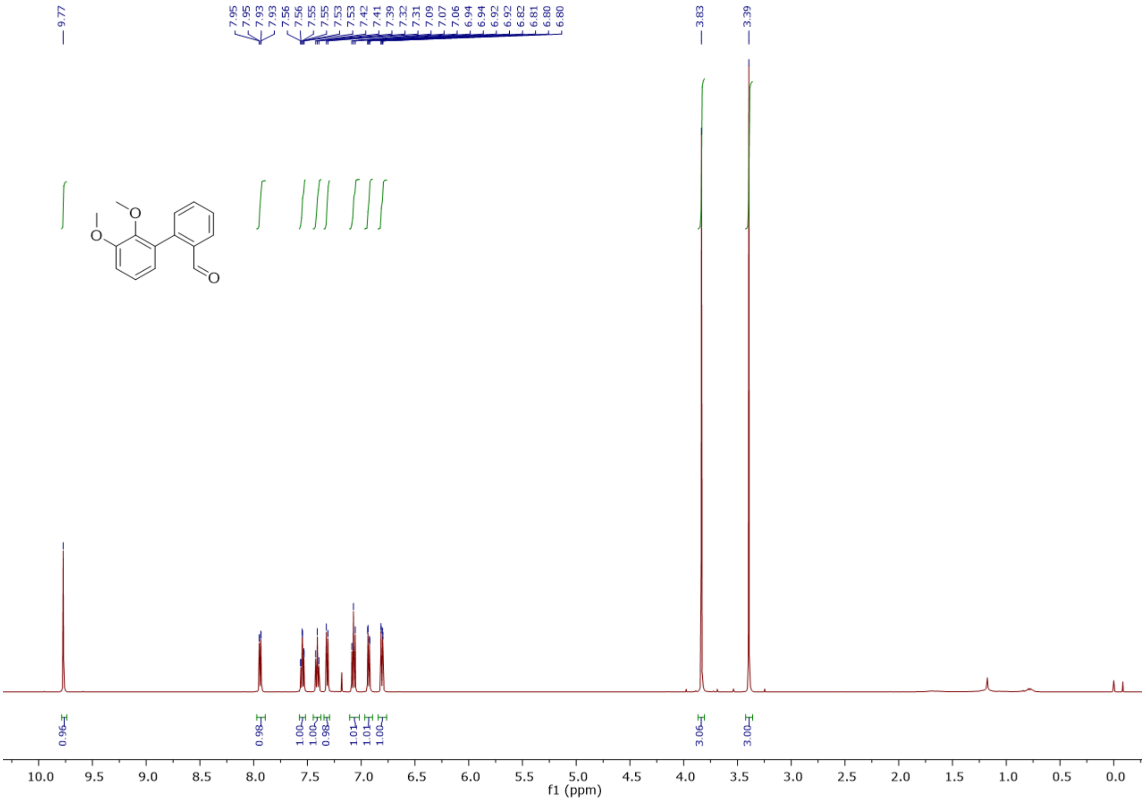

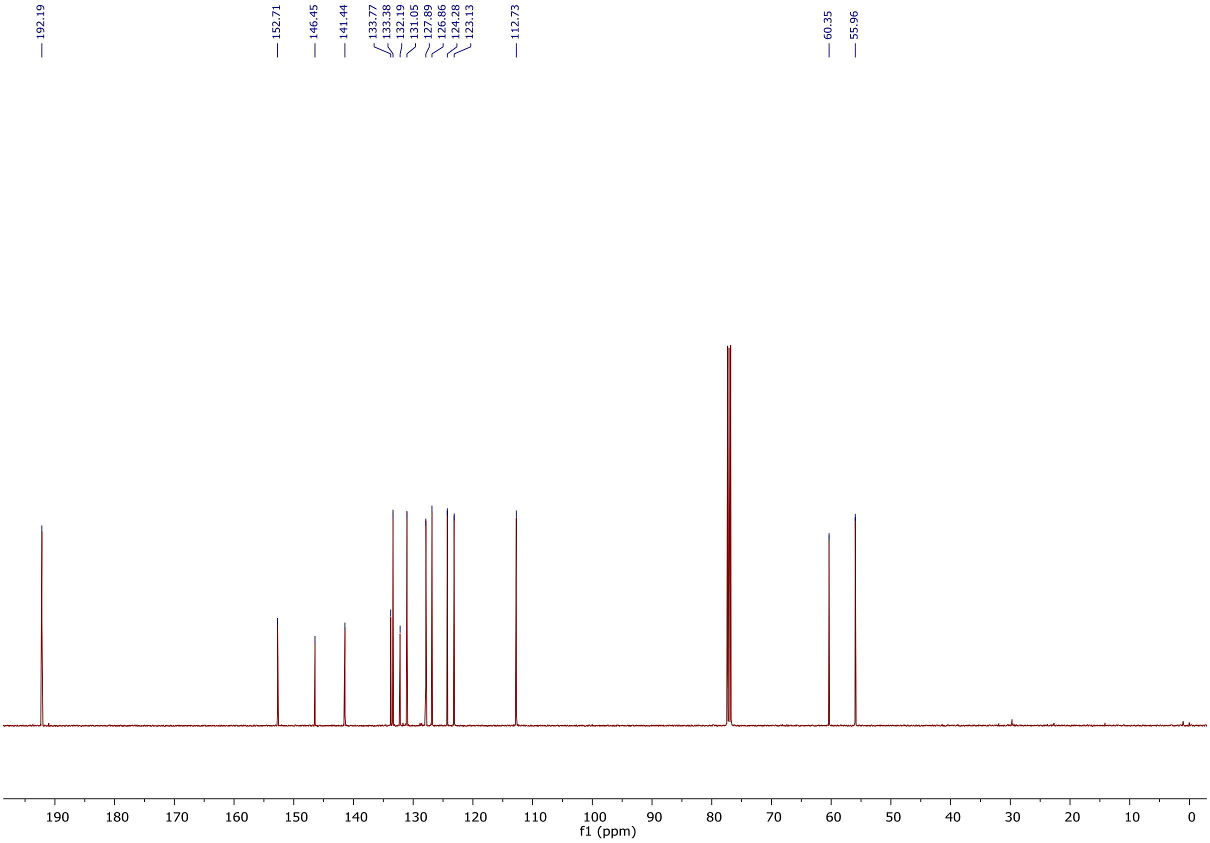

2',4'-Dimethoxy-[1,1'-biphenyl]-2-carbaldehyde (13e)

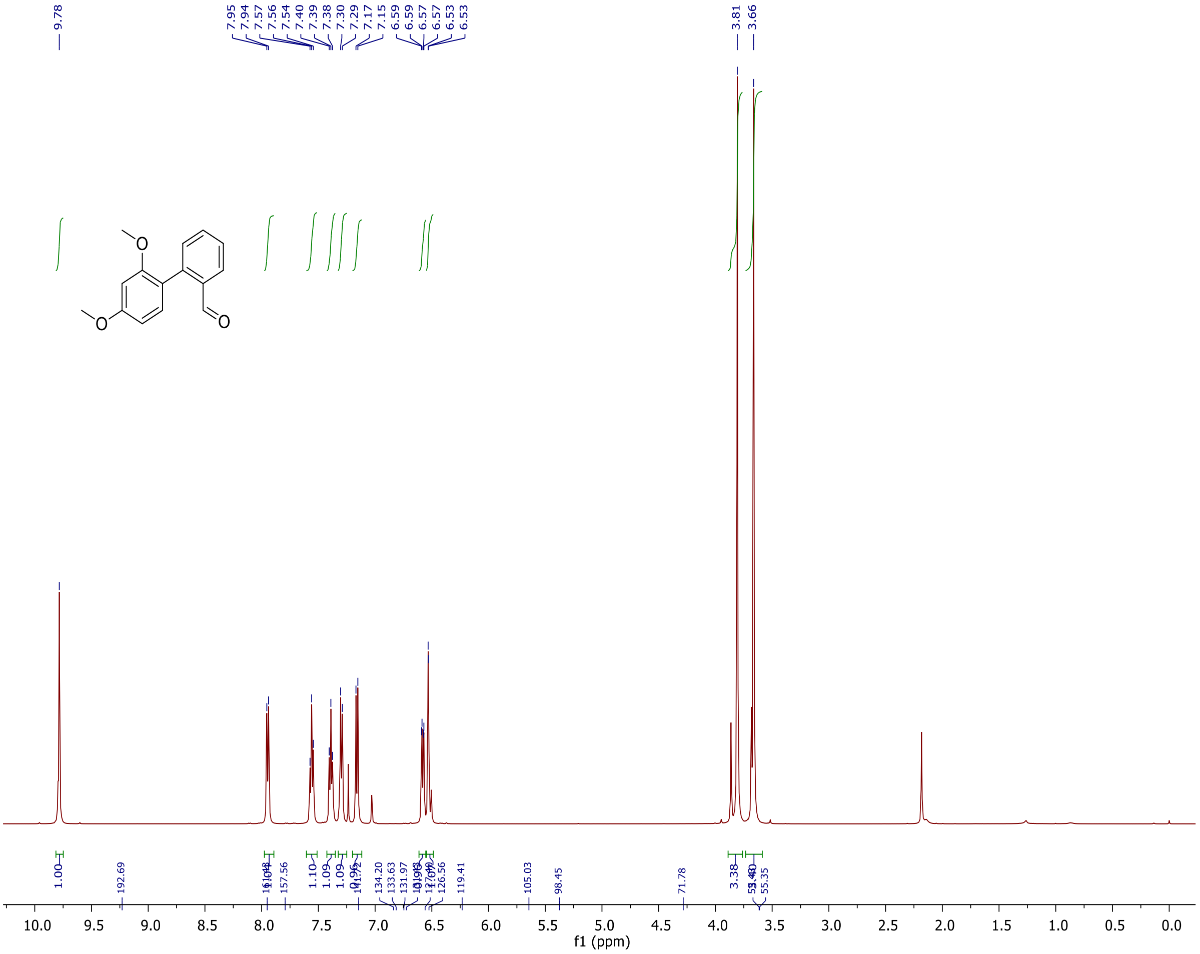

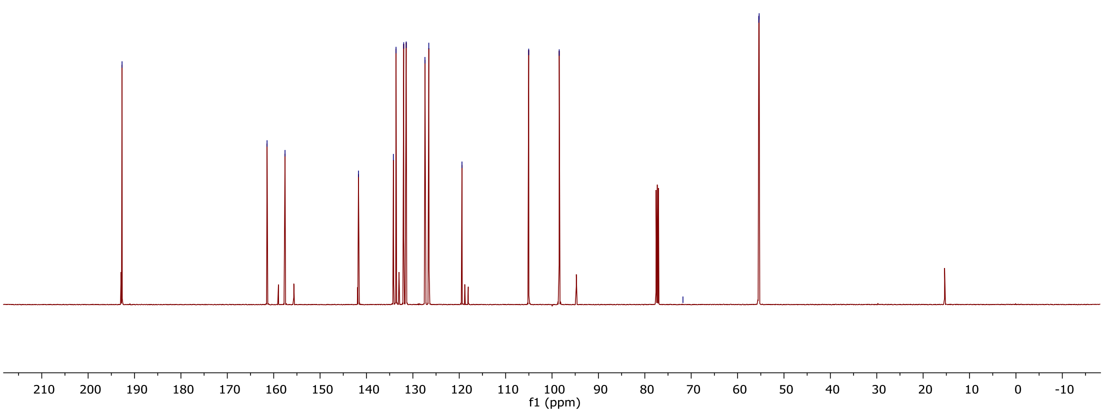

2'-Methoxy-[1,1'-biphenyl]-2-carbaldehyde (13f)

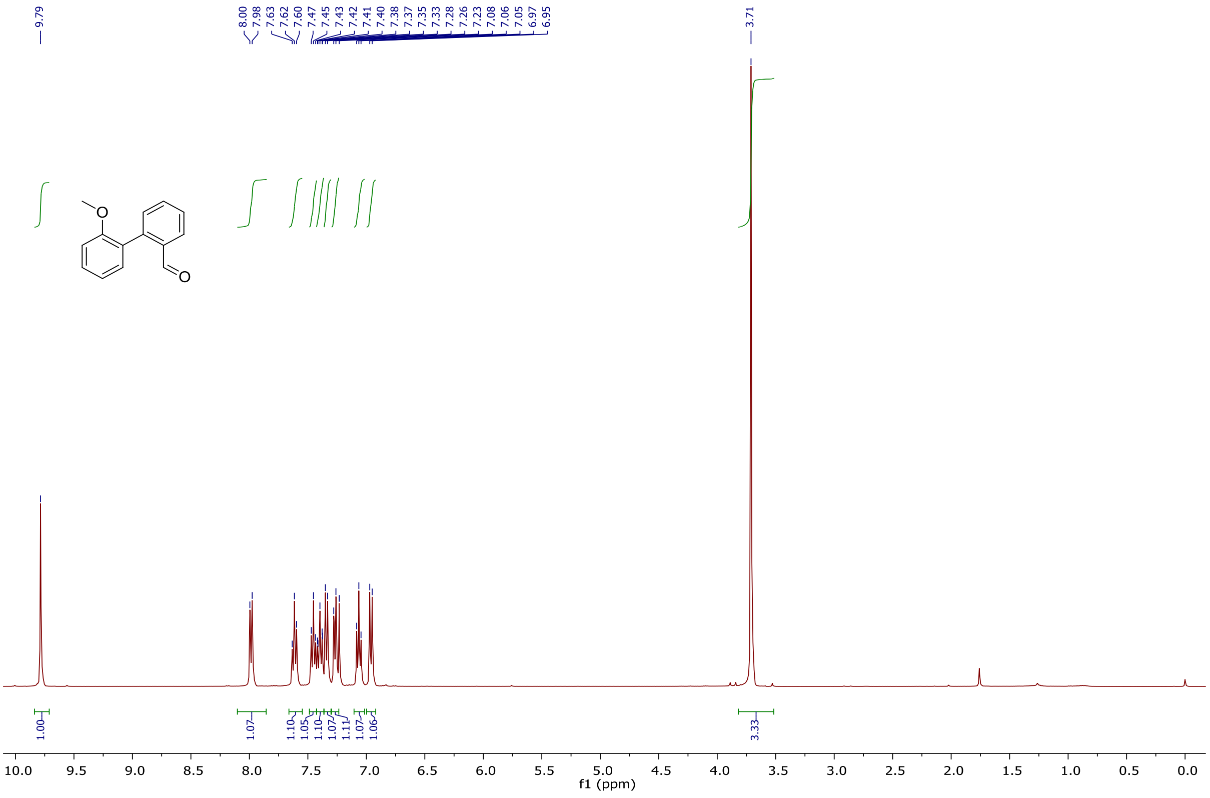

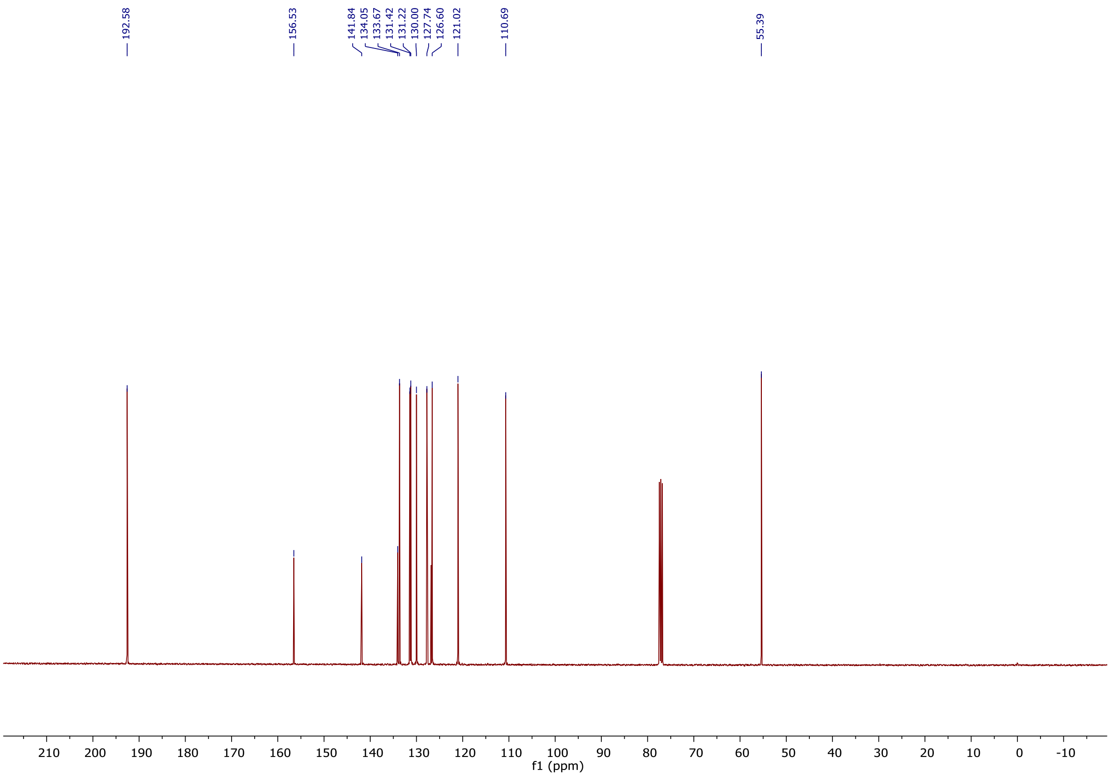

6-(2,4,5-Trimethoxyphenyl)benzo[*d*][1,3]dioxole-5-carbaldehyde (21)

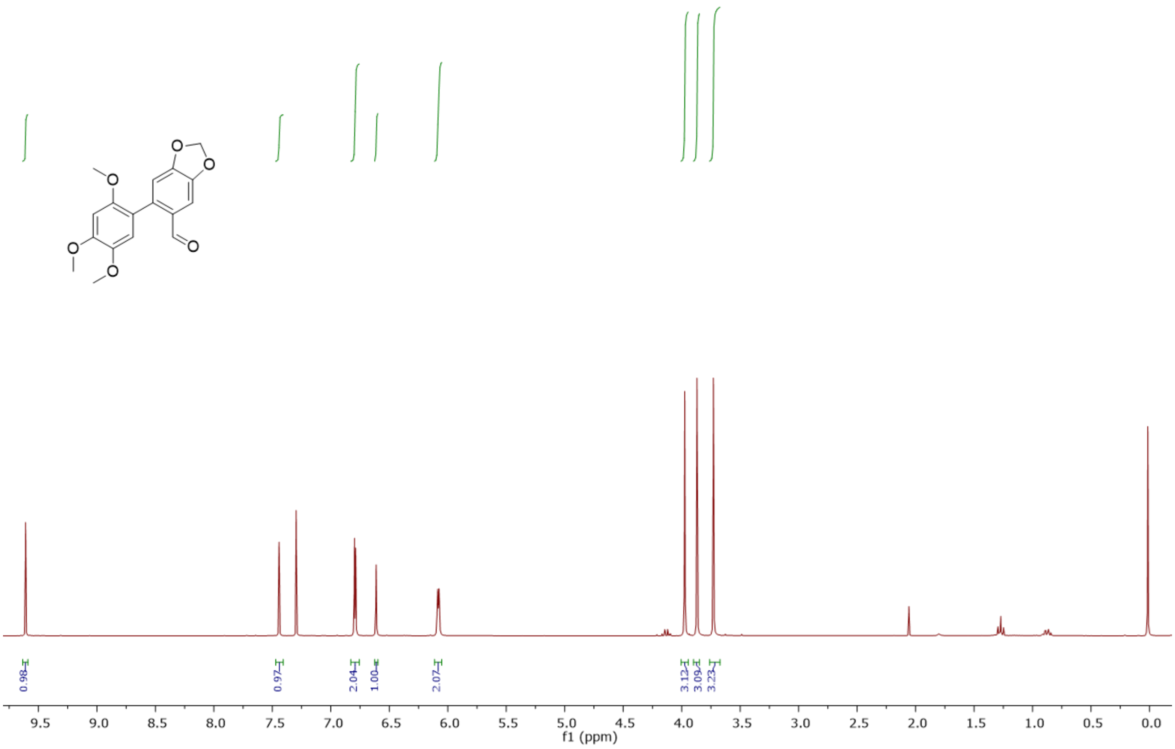

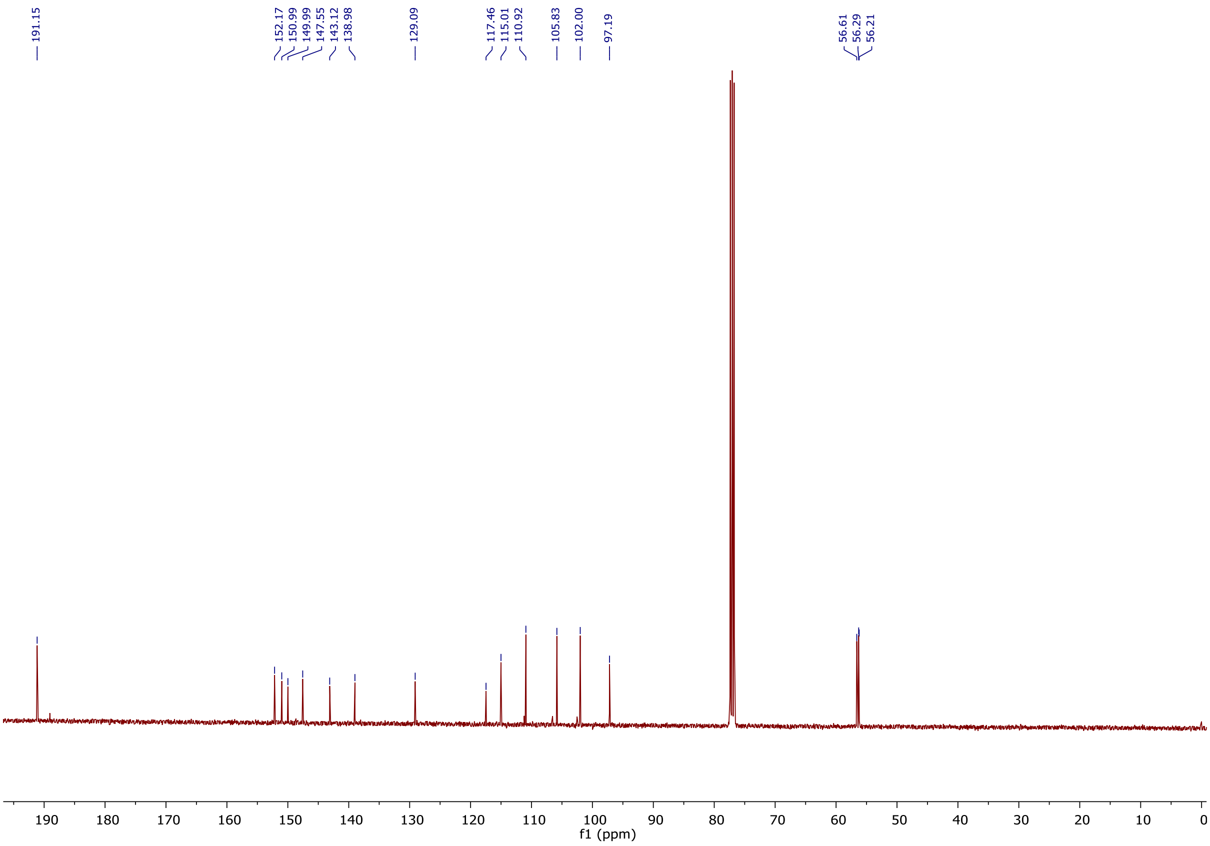

2',4',5'-Trimethoxy-[1,1'-biphenyl]-2-carbaldehyde O-acetyl oxime (14a)

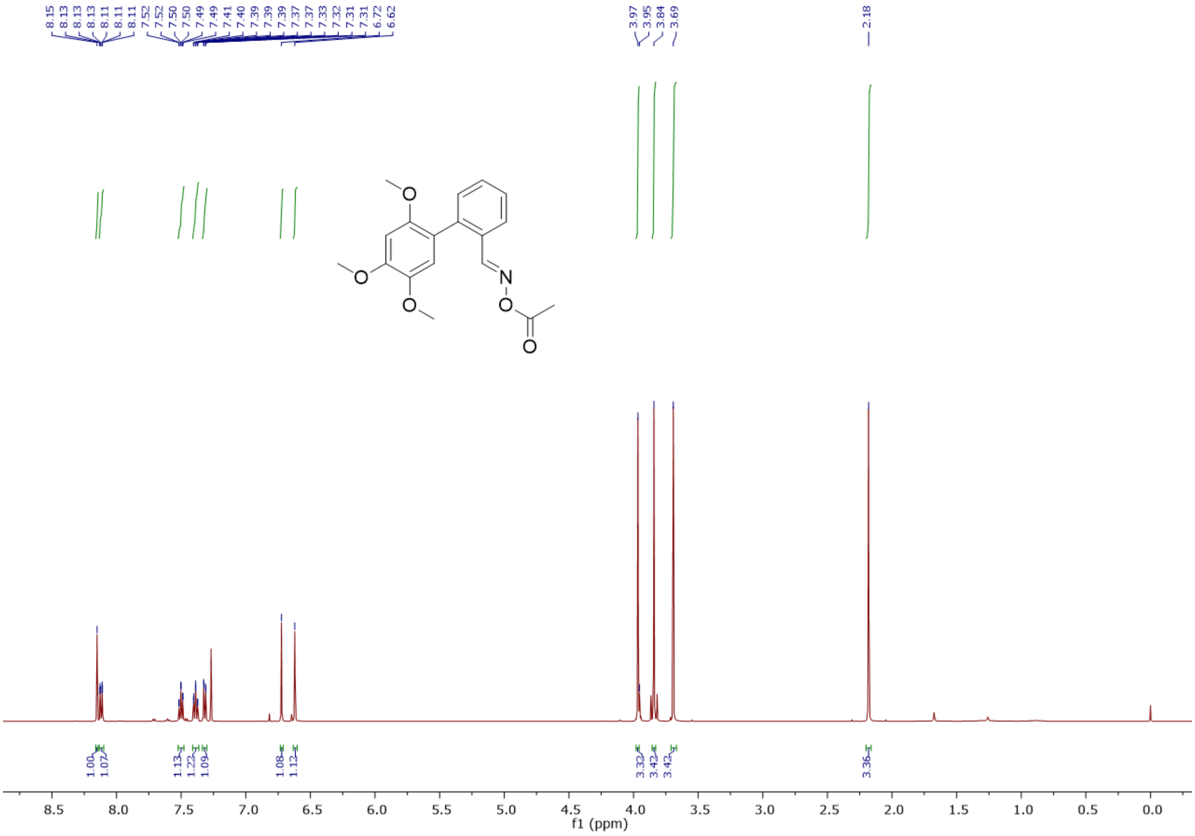

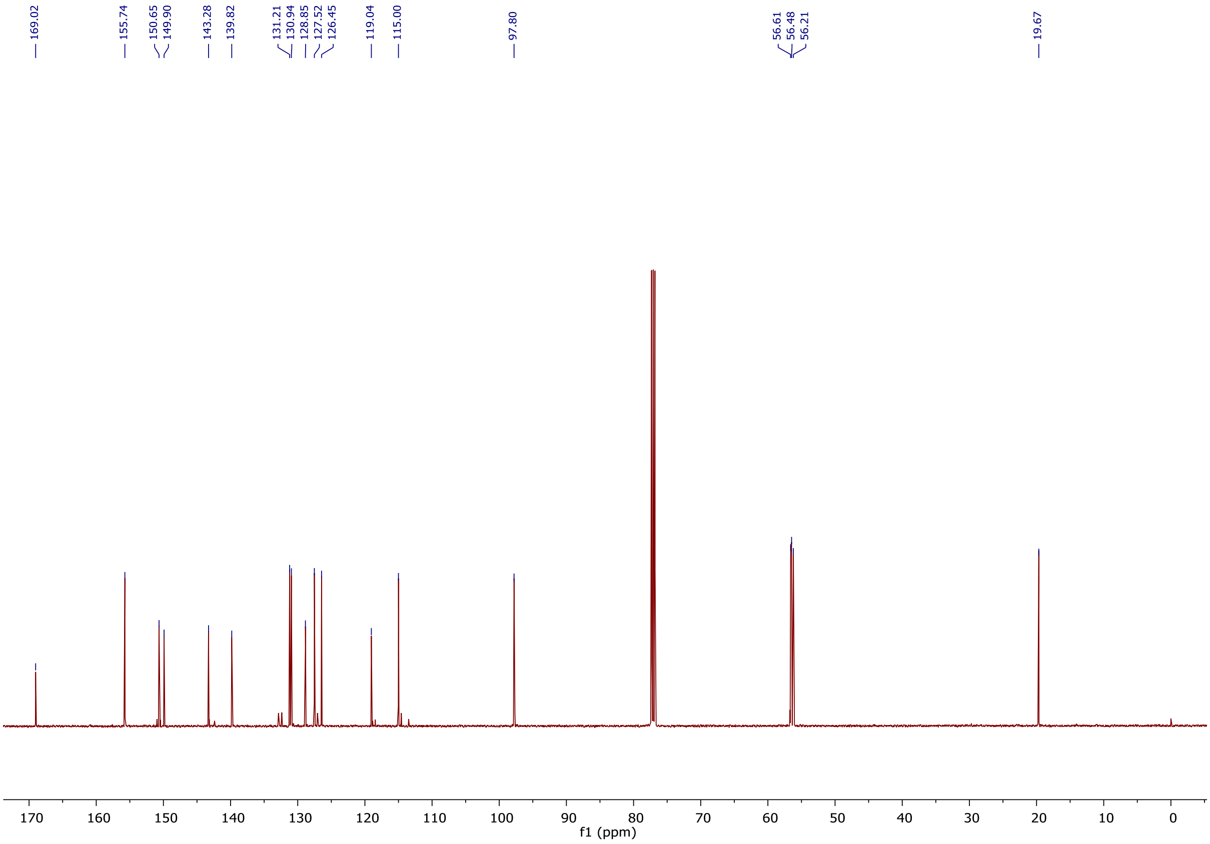

2',4',6'-Trimethoxy-[1,1'-biphenyl]-2-carbaldehyde *O*-acetyl oxime (14b)

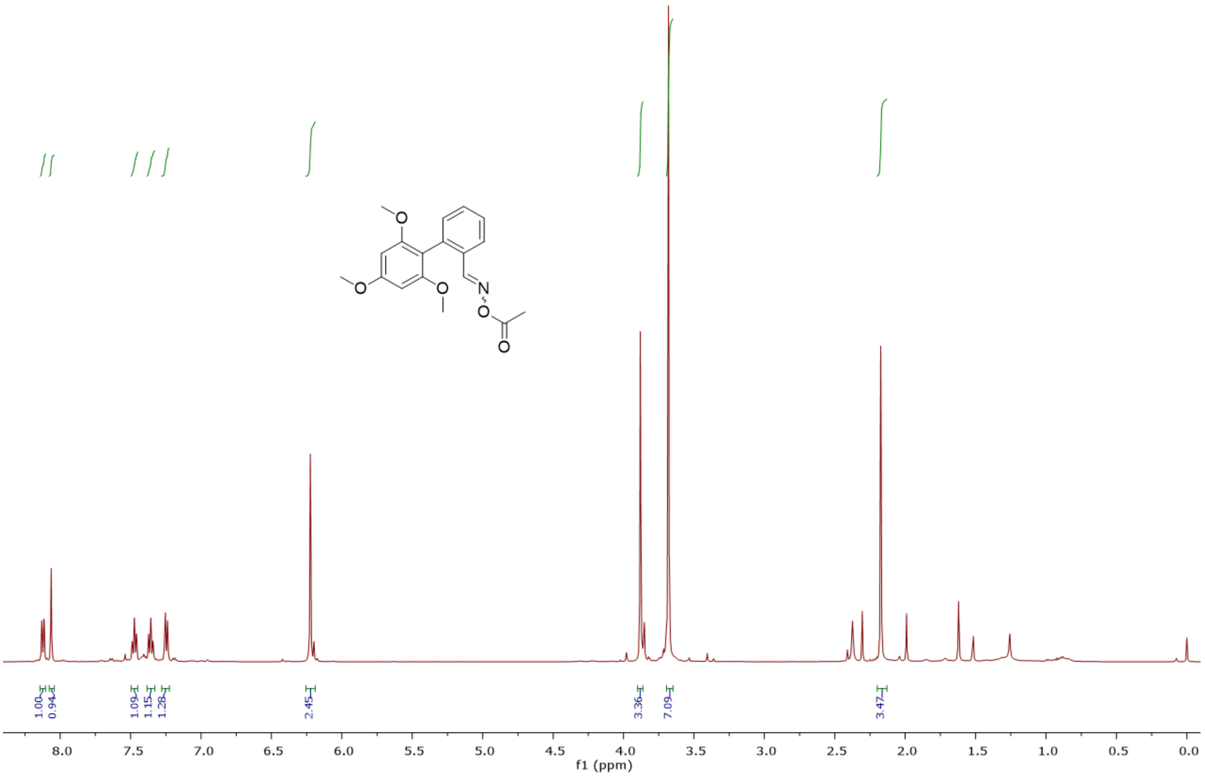

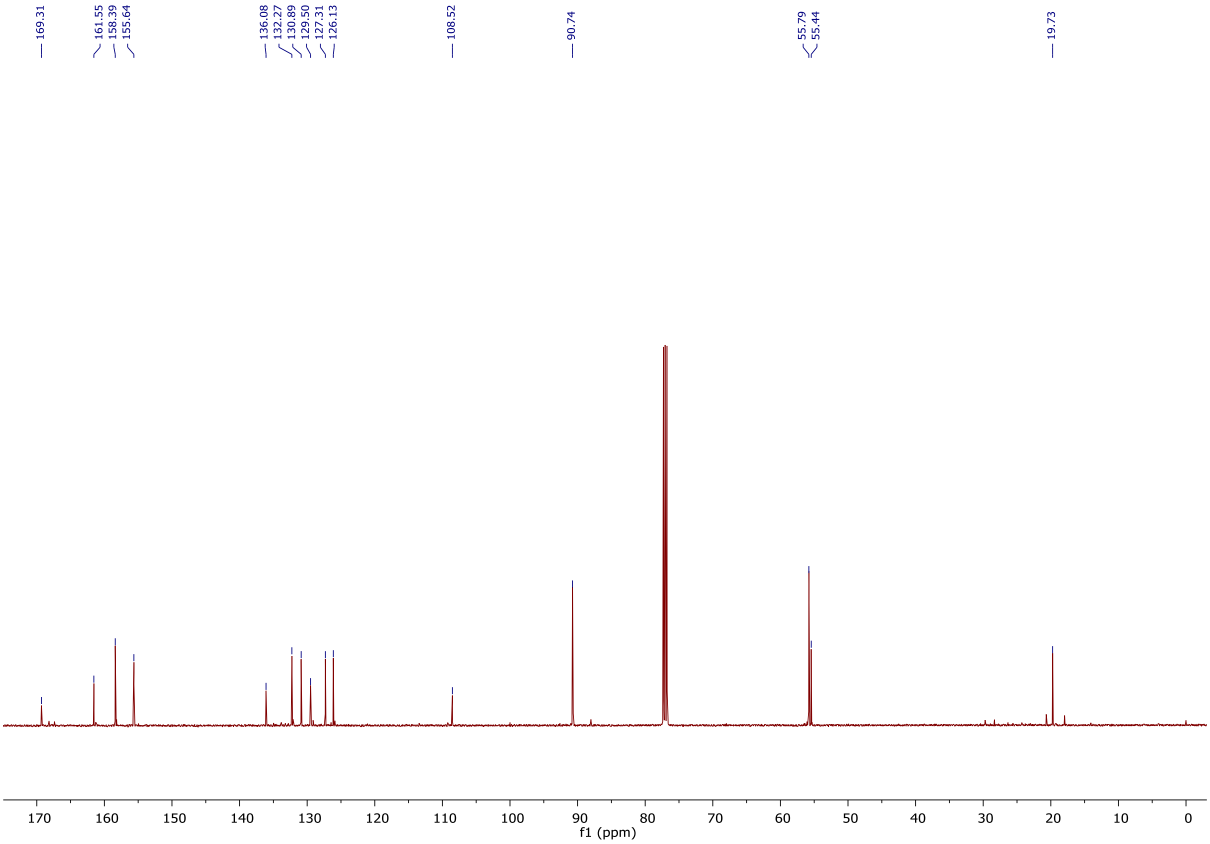

2',5'-Dimethoxy-[1,1'-biphenyl]-2-carbaldehyde O-acetyl oxime (14c)

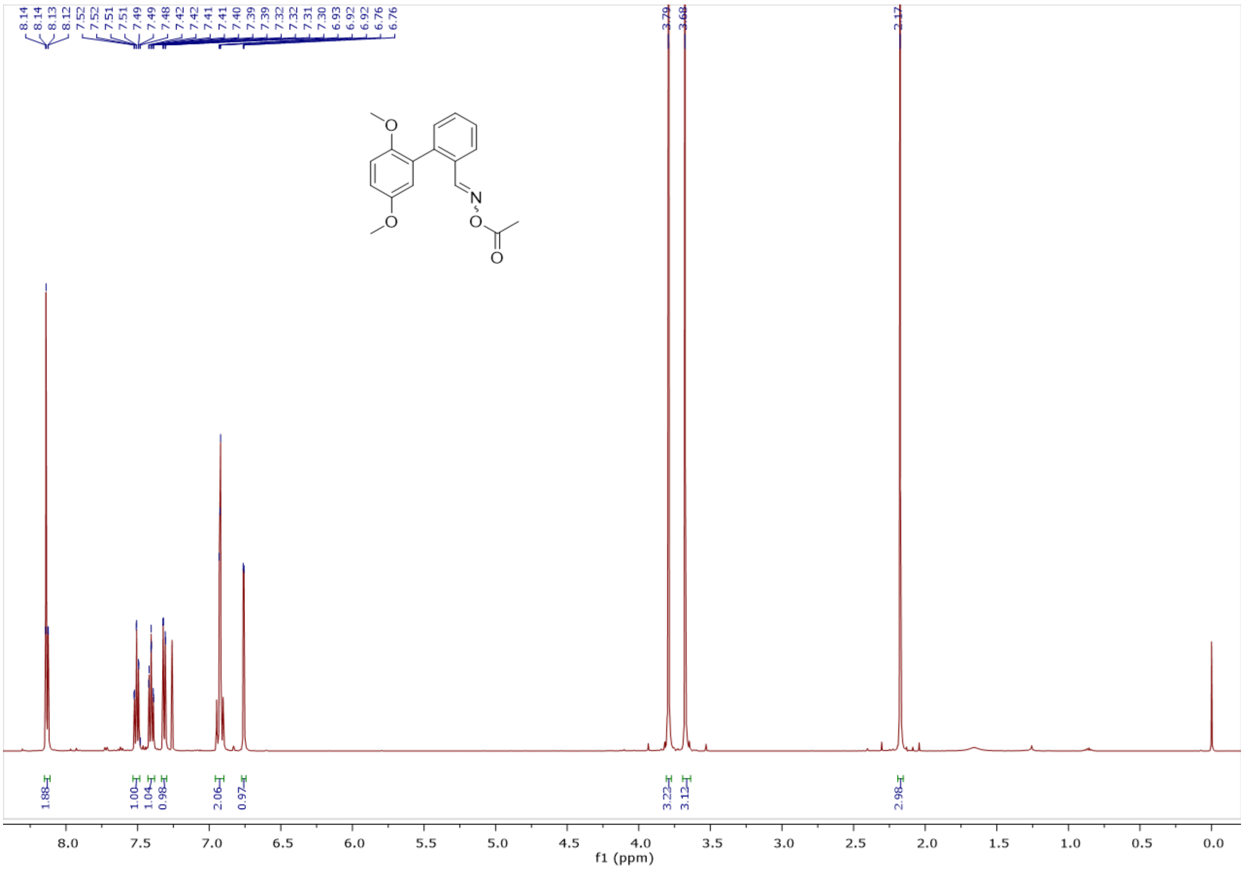

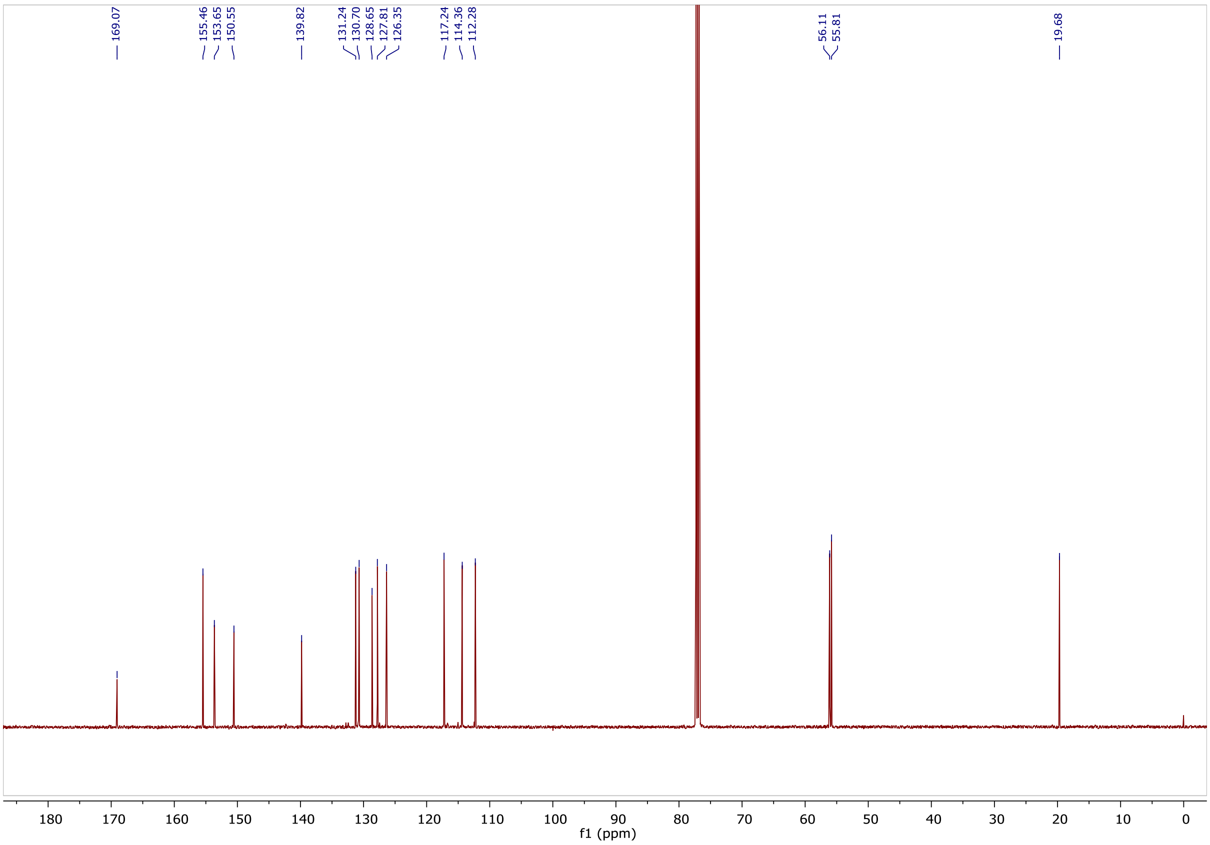

2',3'-Dimethoxy-[1,1'-biphenyl]-2-carbaldehyde *O*-acetyl oxime (14d)

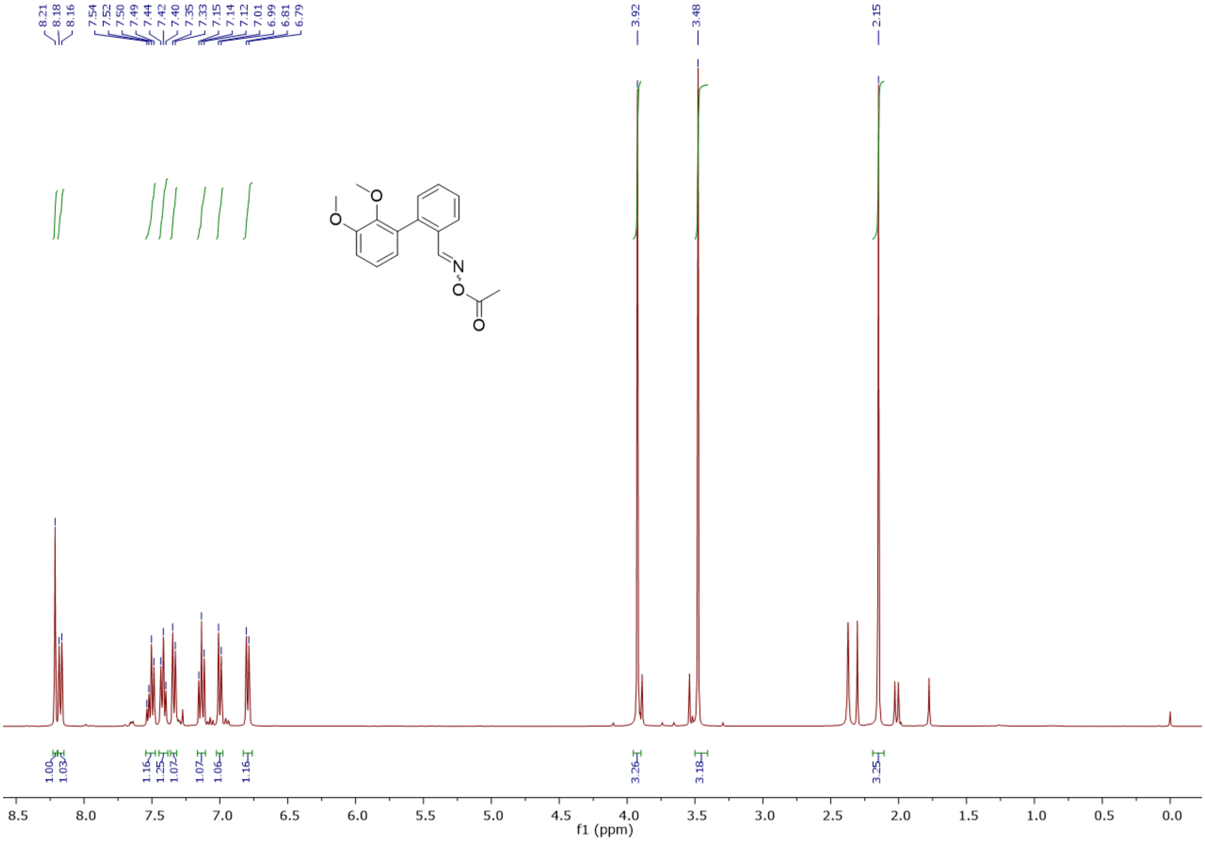

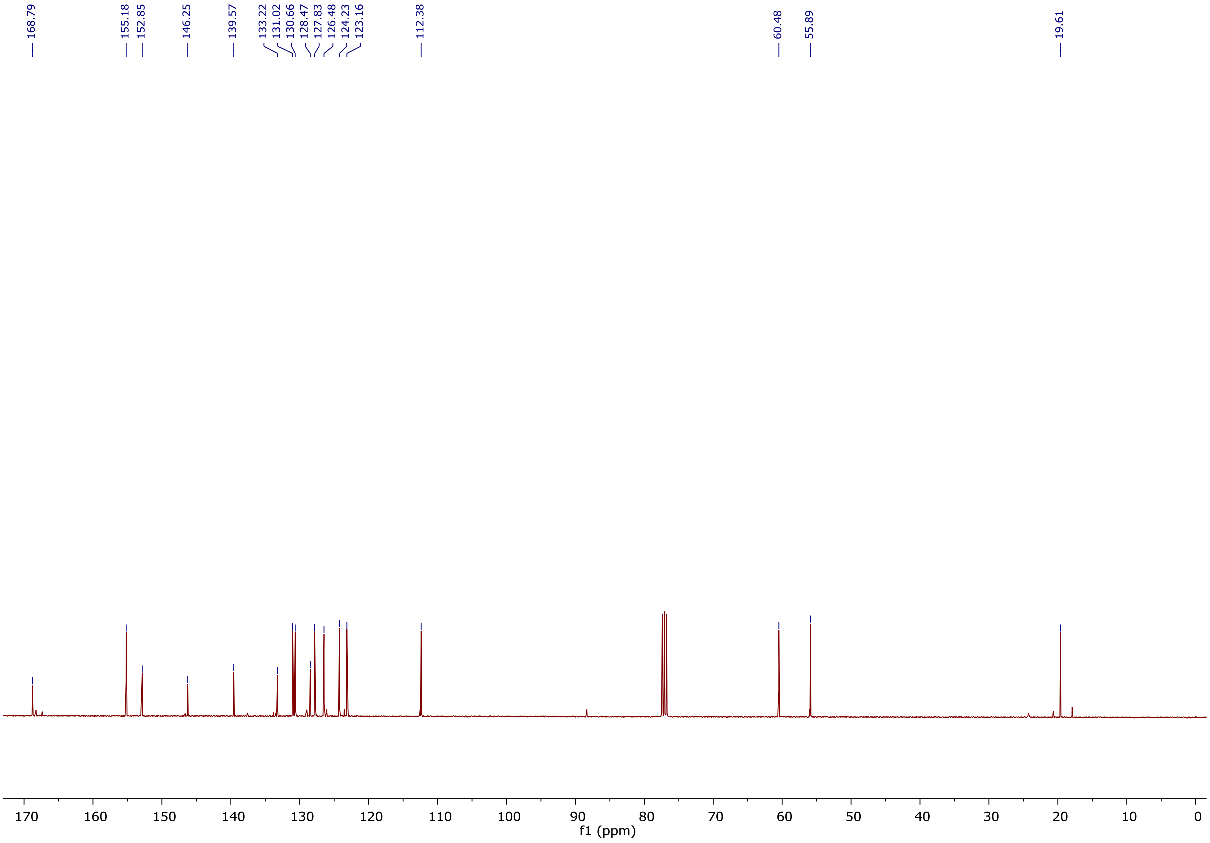

2',4'-Dimethoxy-[1,1'-biphenyl]-2-carbaldehyde O-acetyl oxime (14e)

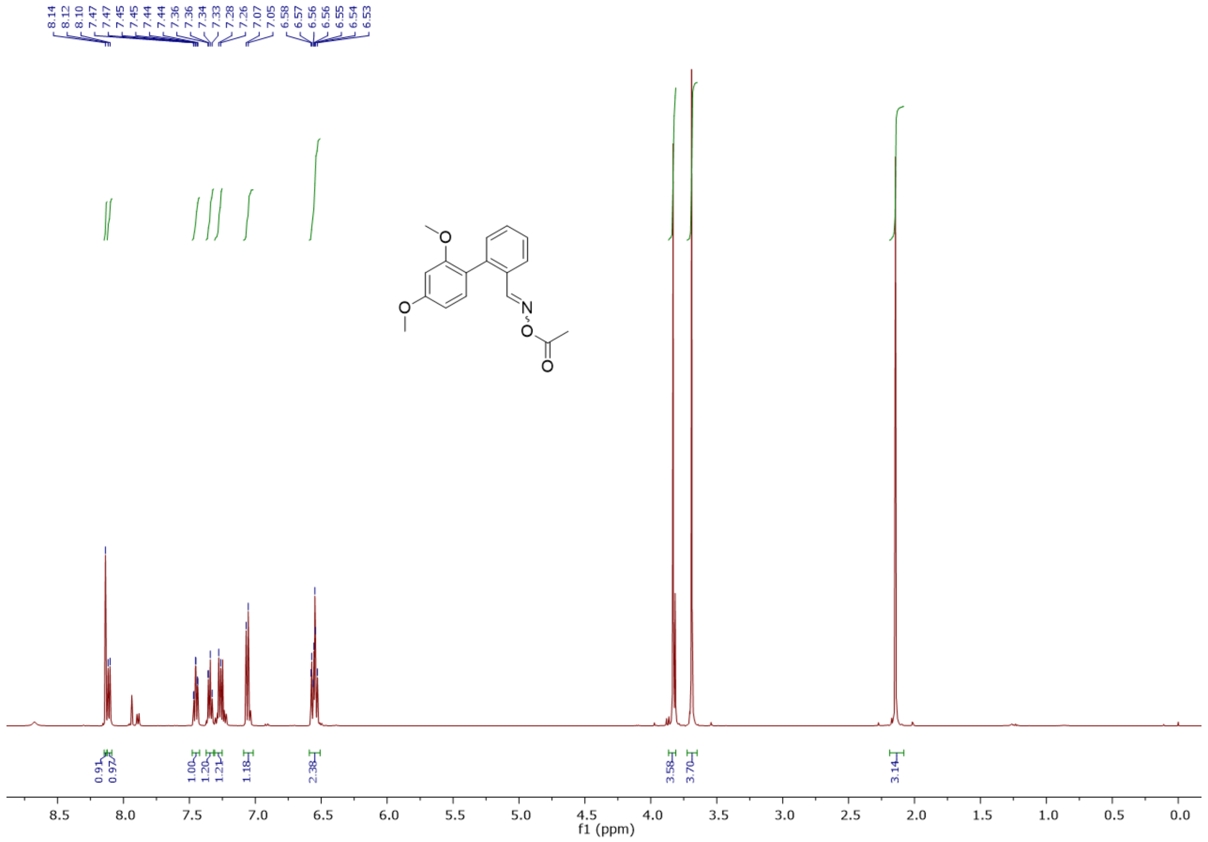

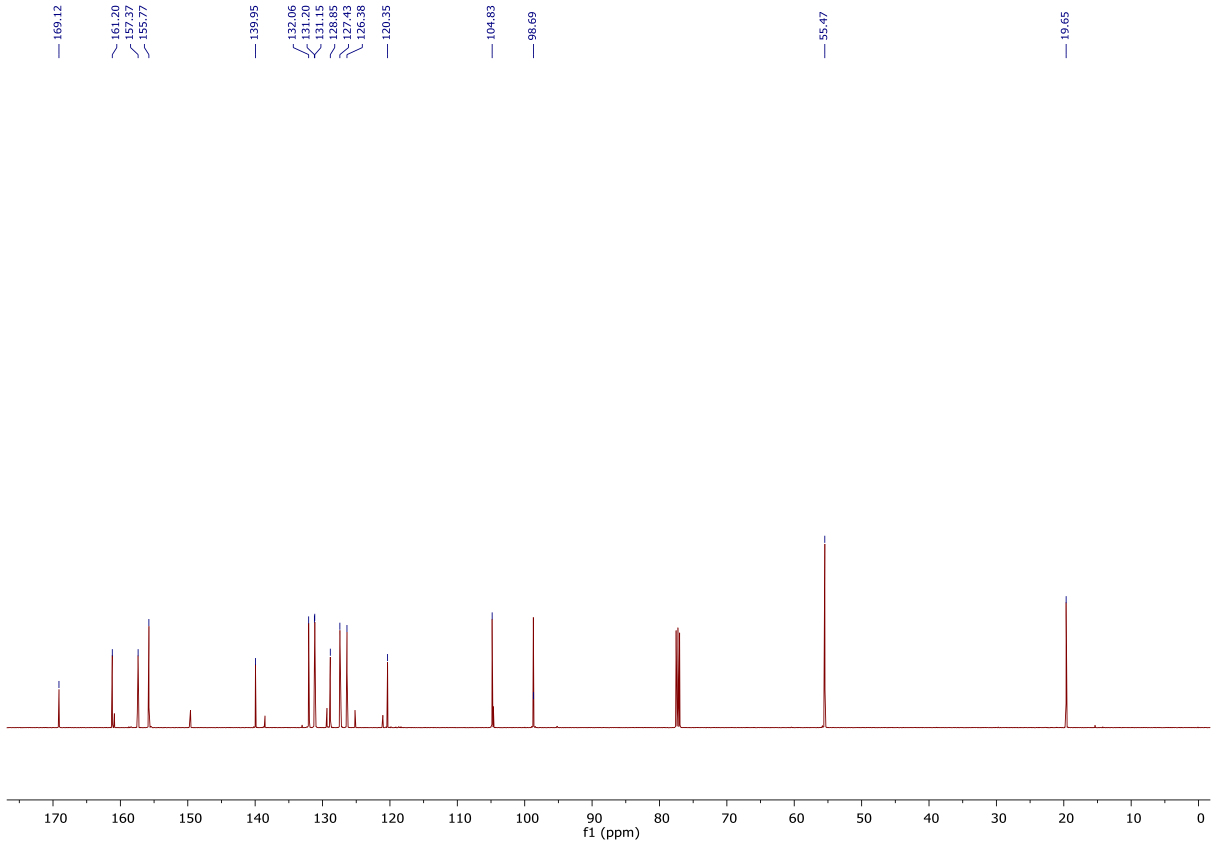

2'-Methoxy-[1,1'-biphenyl]-2-carbaldehyde O-acetyl oxime (14f)

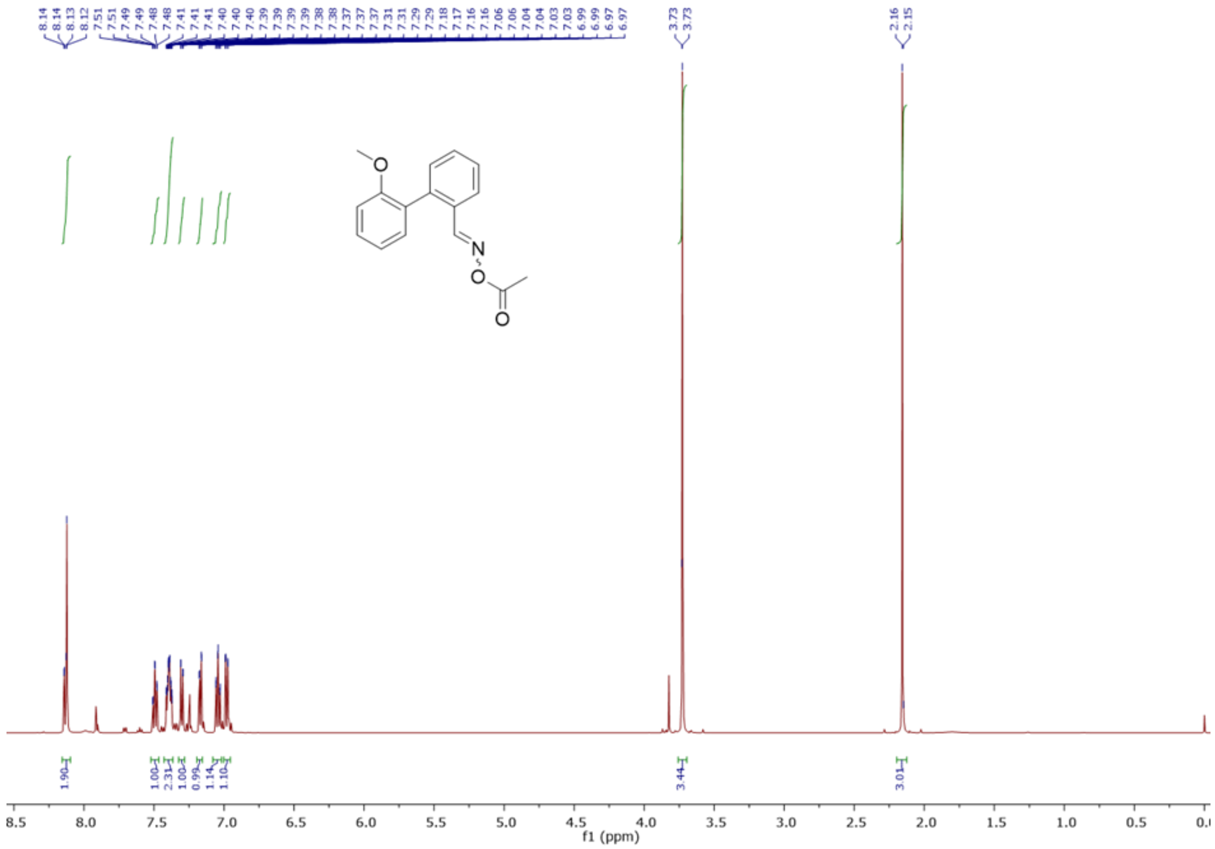

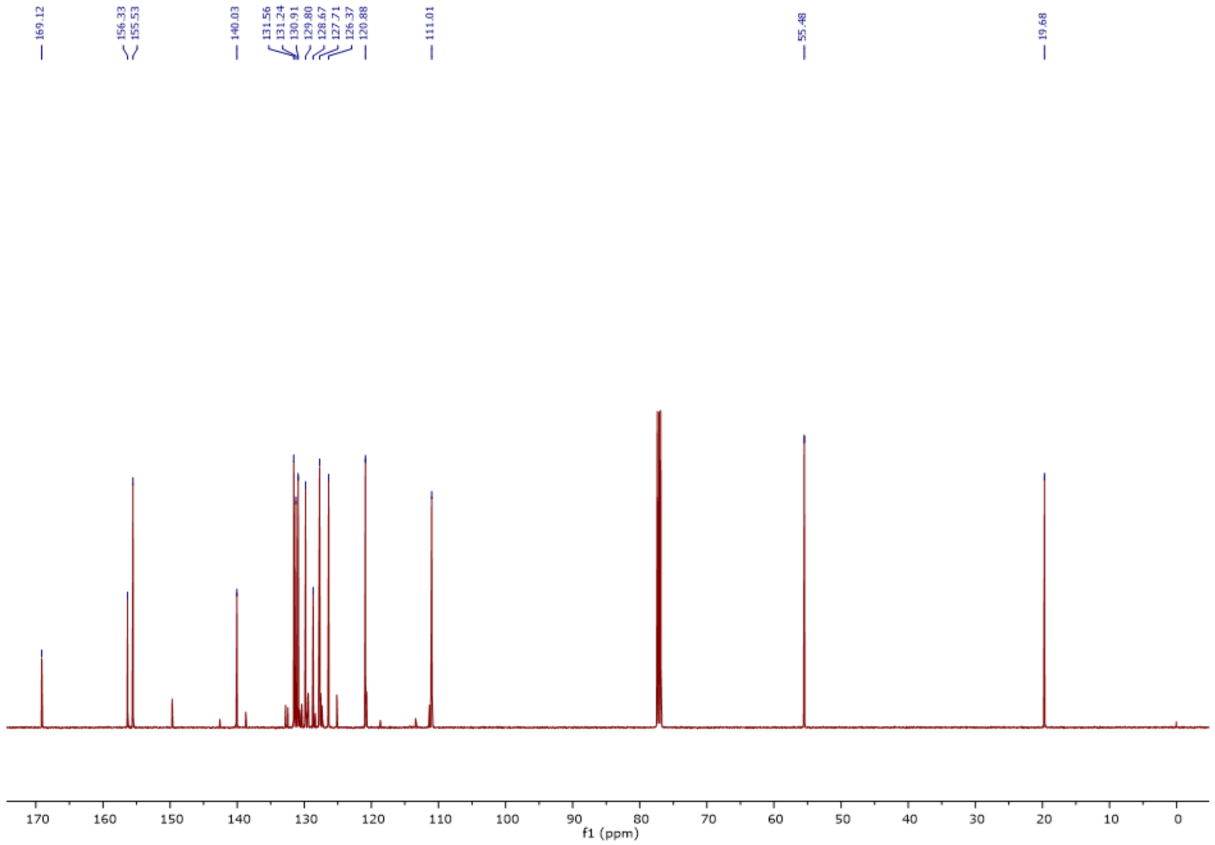

6-(2,4,5-Trimethoxyphenyl)benzo[d][1,3]dioxole-5-carbaldehyde *O*-acetyl oxime (22)

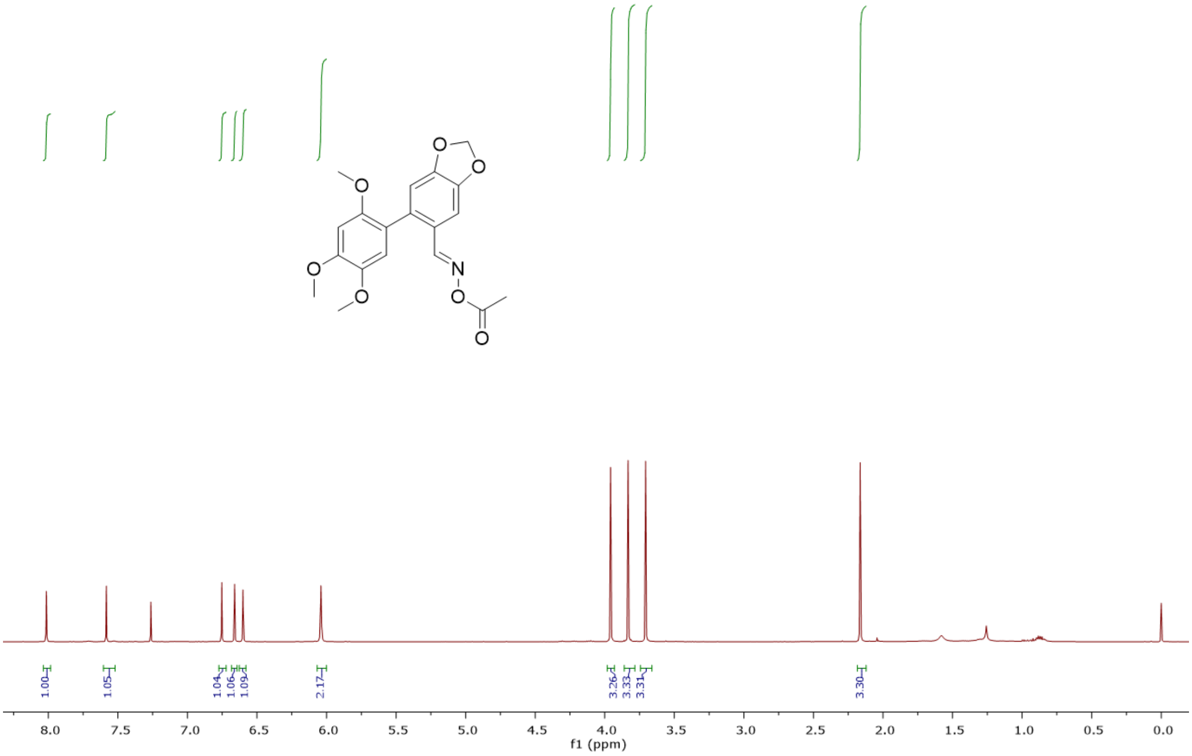

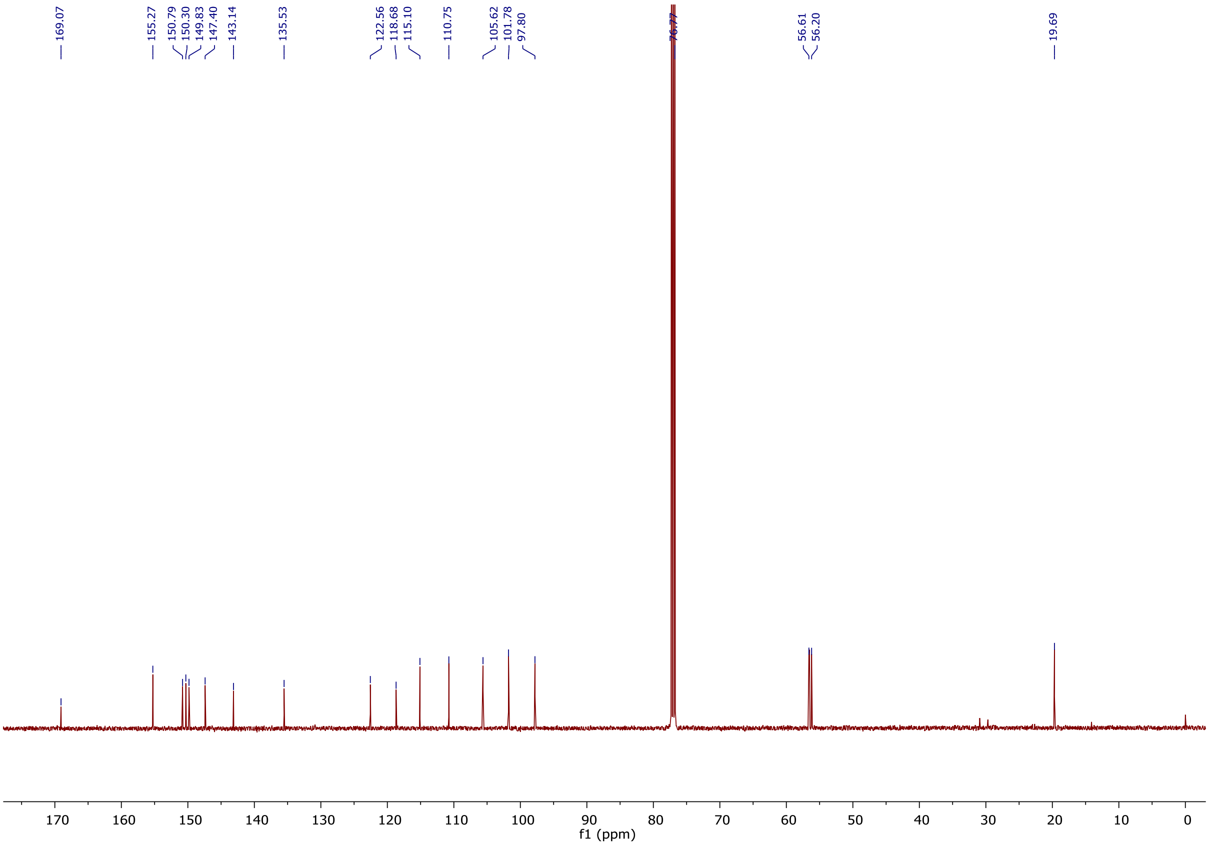

2',4',5'-Trimethoxy-[1,1'-biphenyl]-2-carbonitrile (16a)

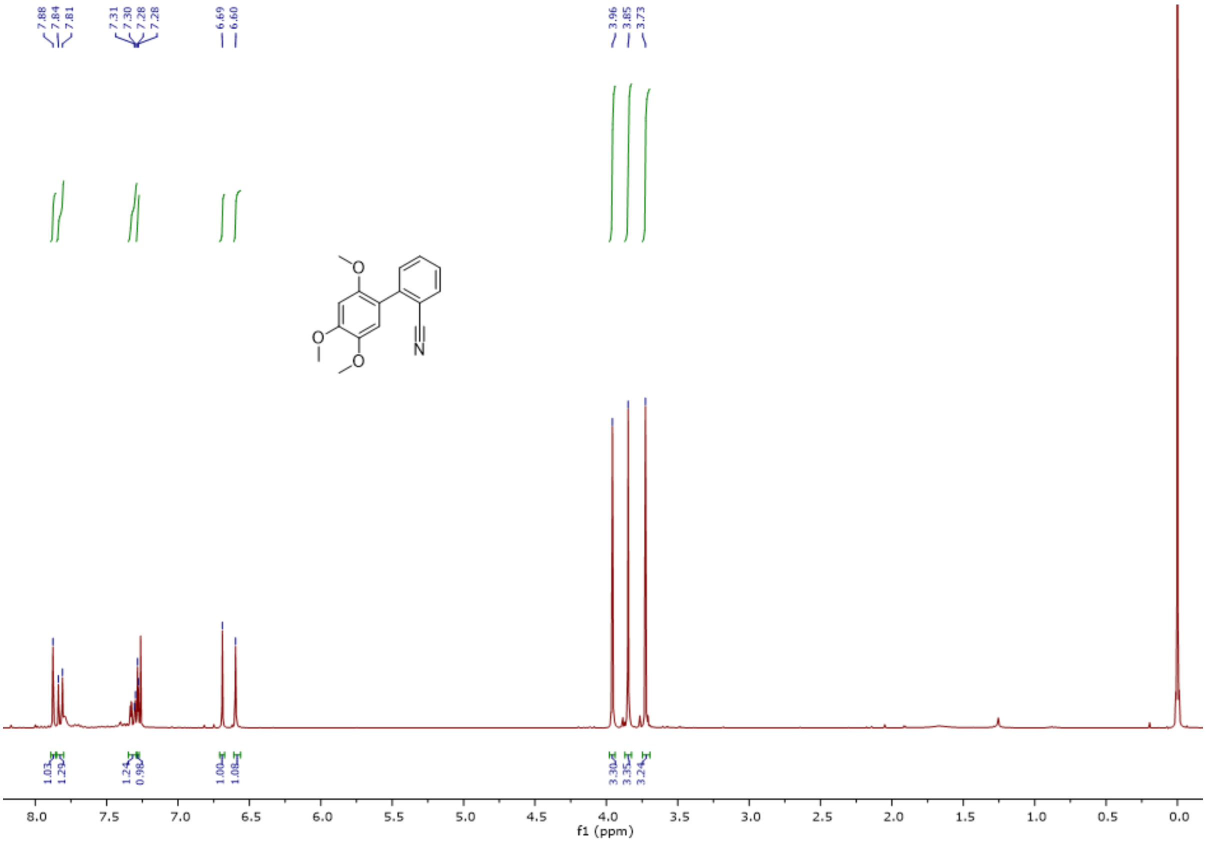

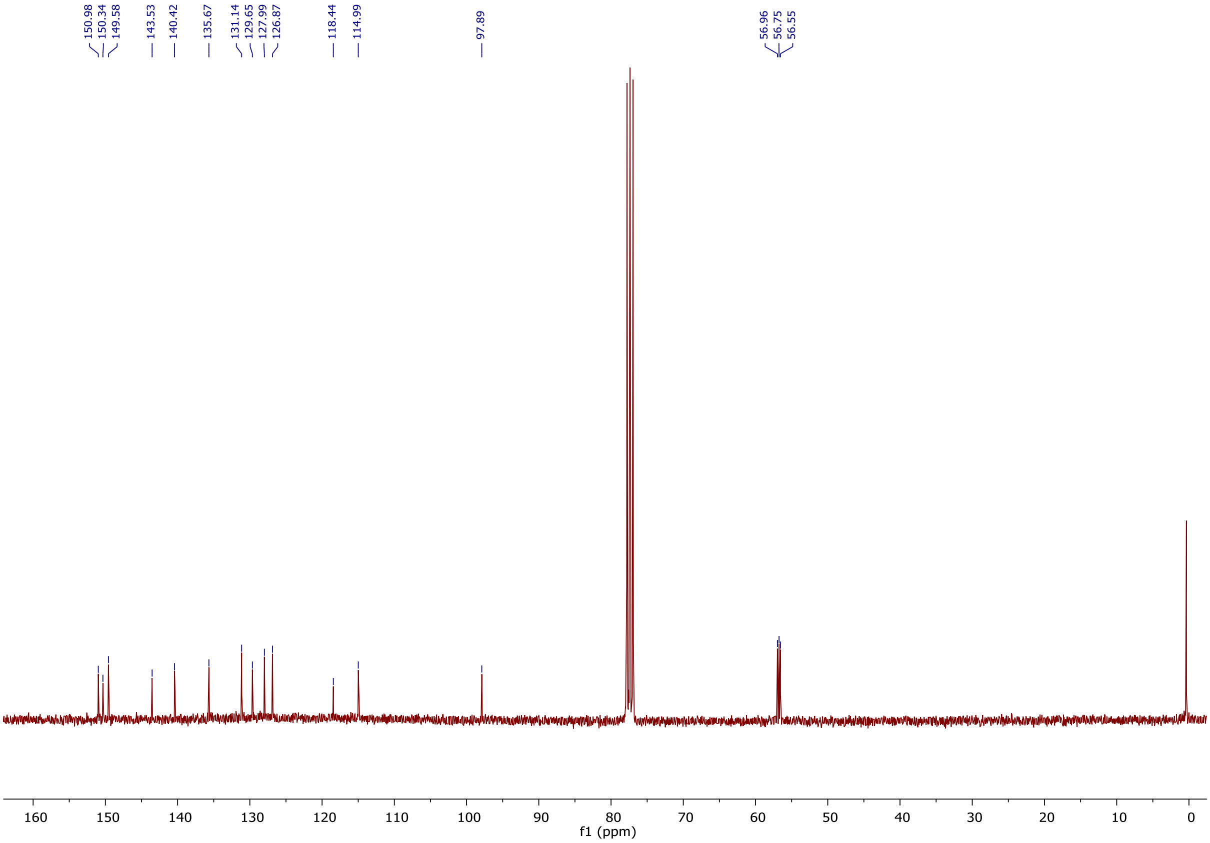

2',4',6'-Trimethoxy-[1,1'-biphenyl]-2-carbonitrile (16b)

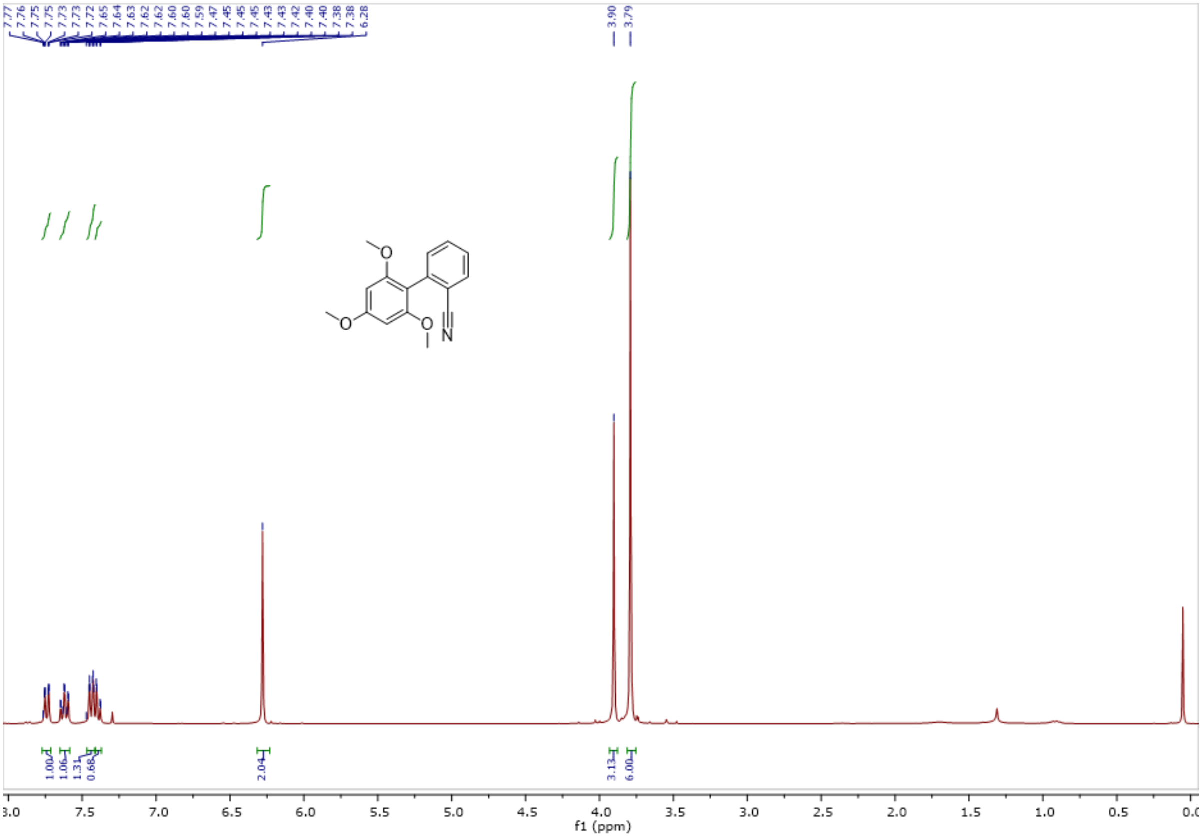

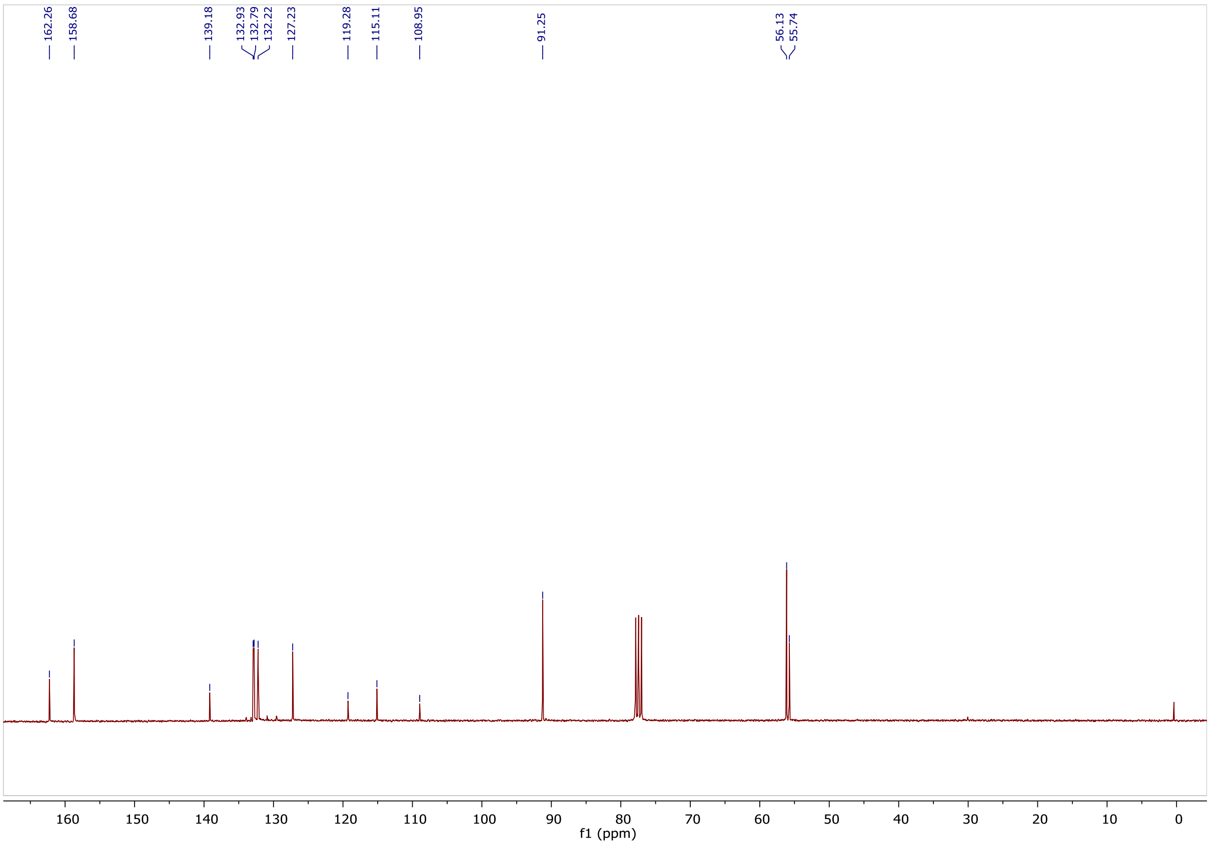

2-Methoxyphenanthridine (15c)

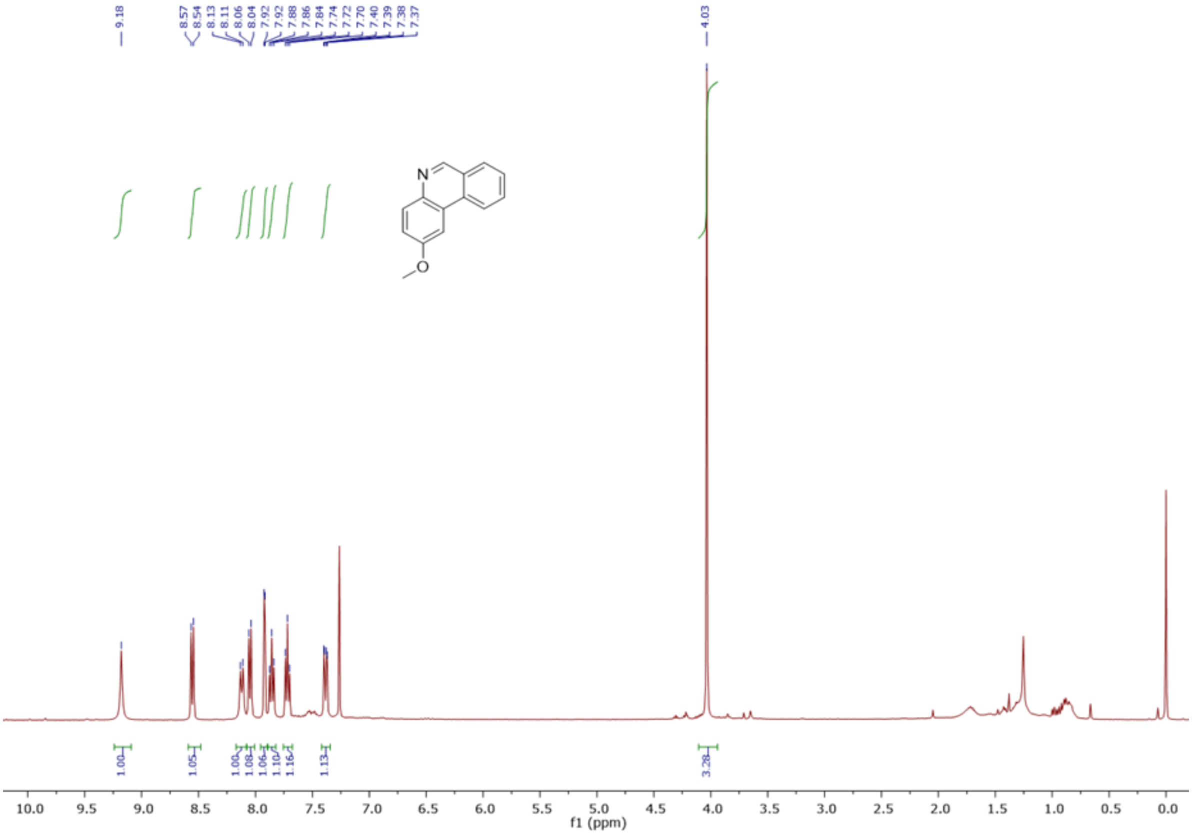

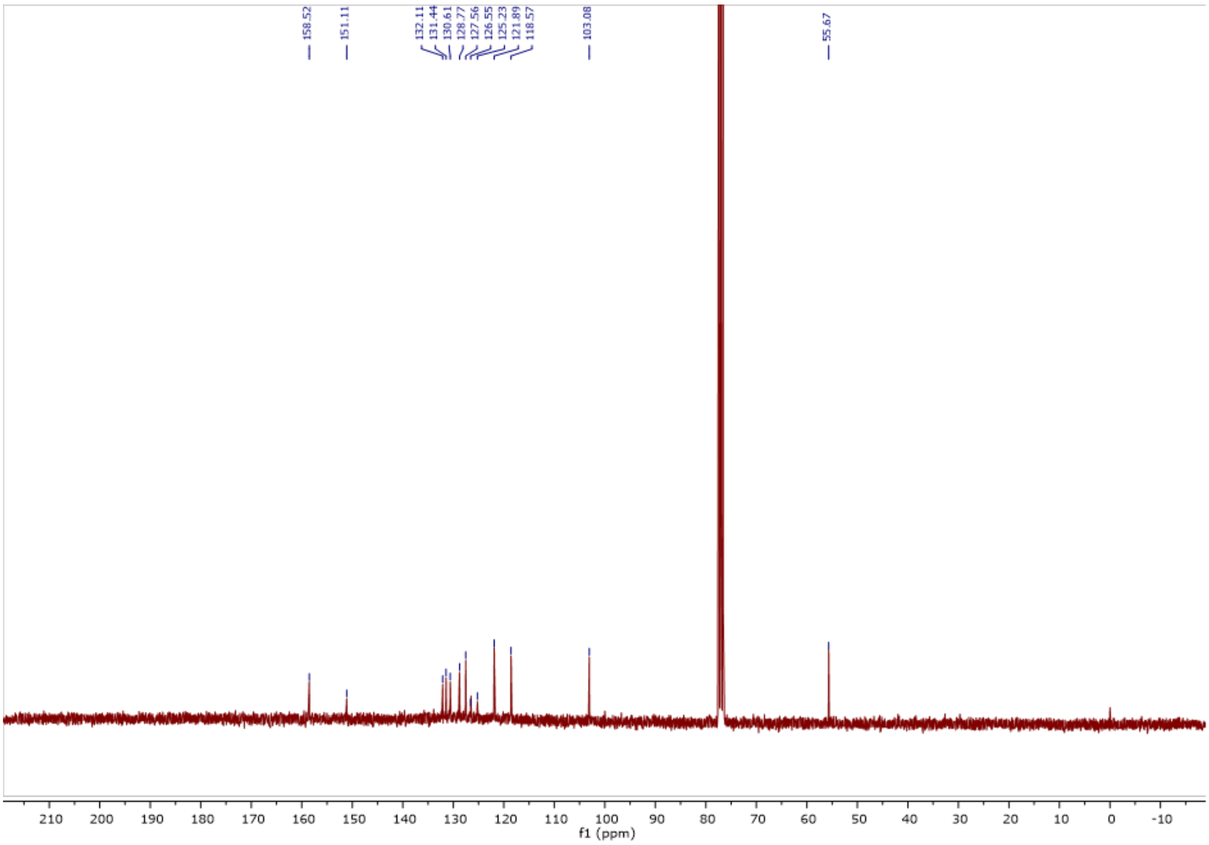

2',5'-Dimethoxy-[1,1'-biphenyl]-2-carbonitrile (16c)

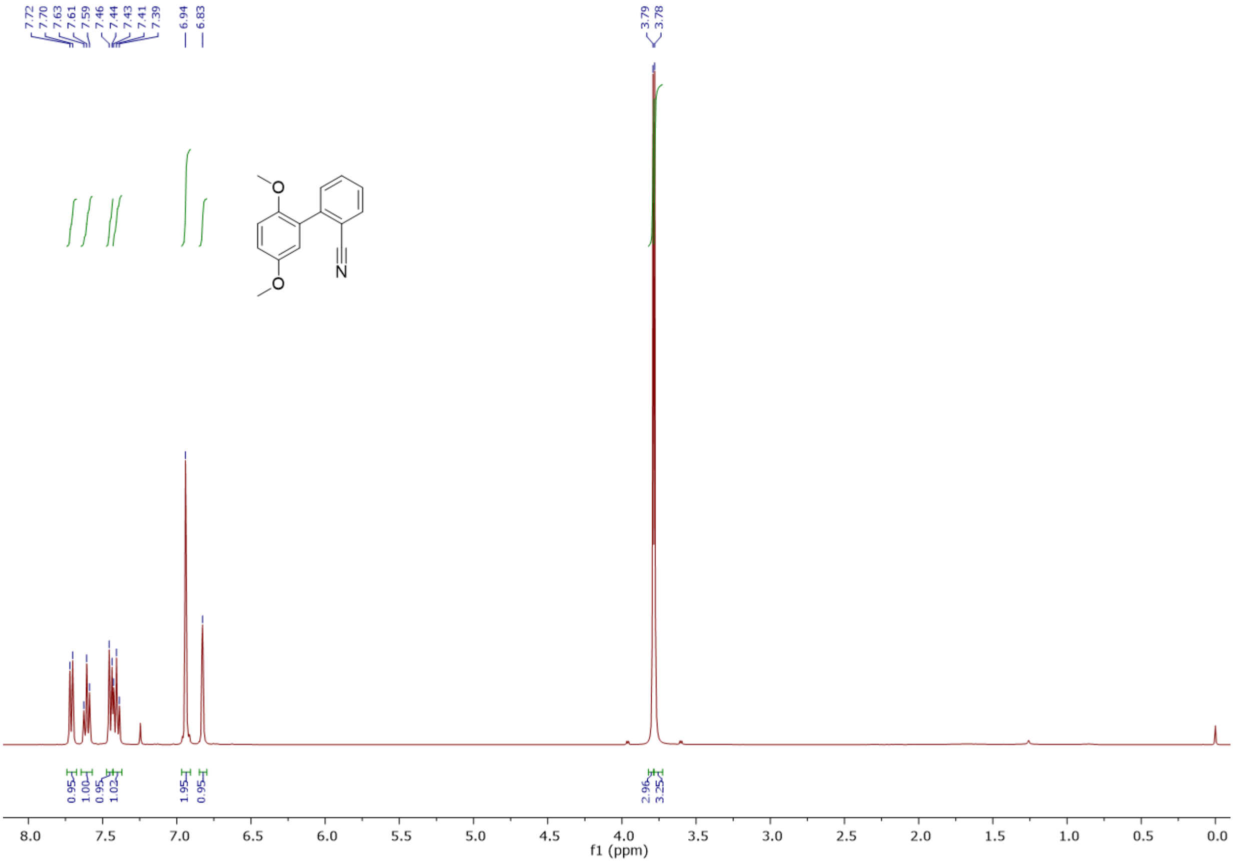

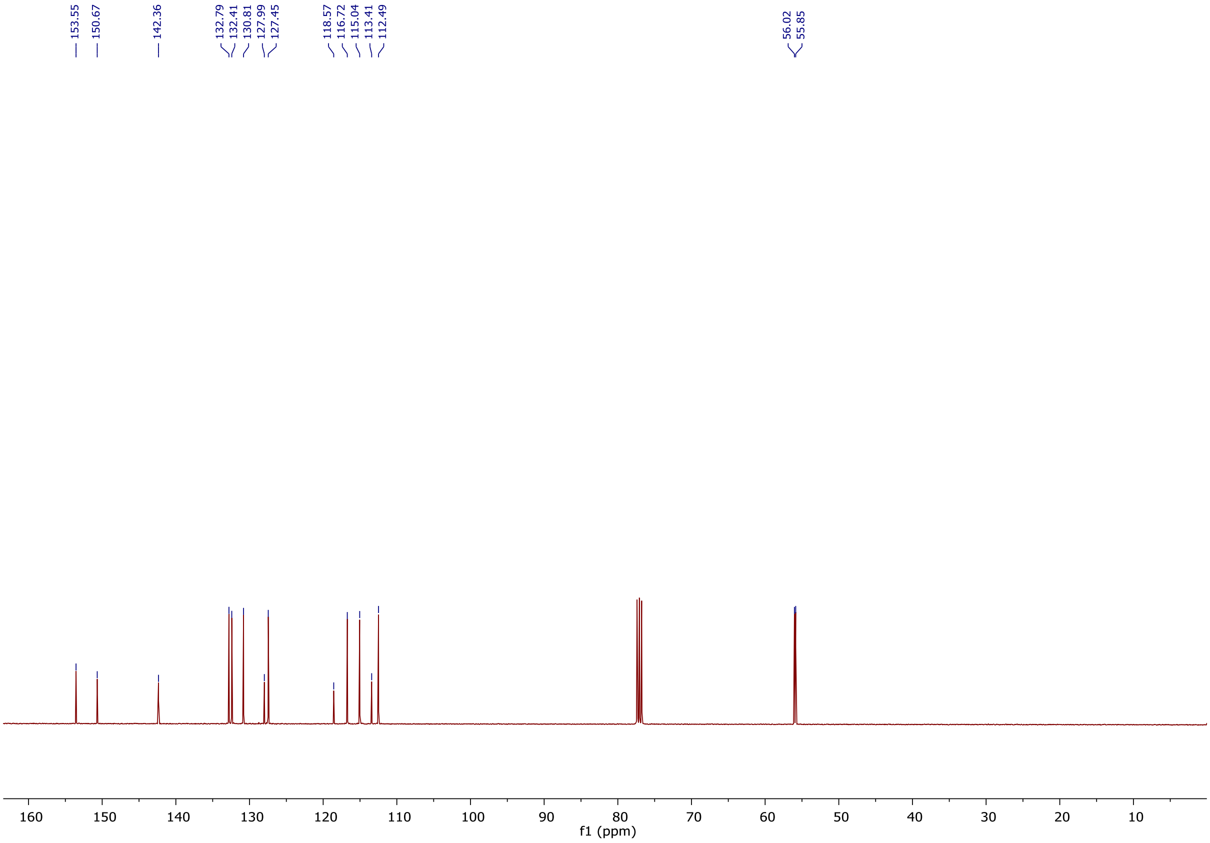

4-Methoxyphenanthridine (15d)

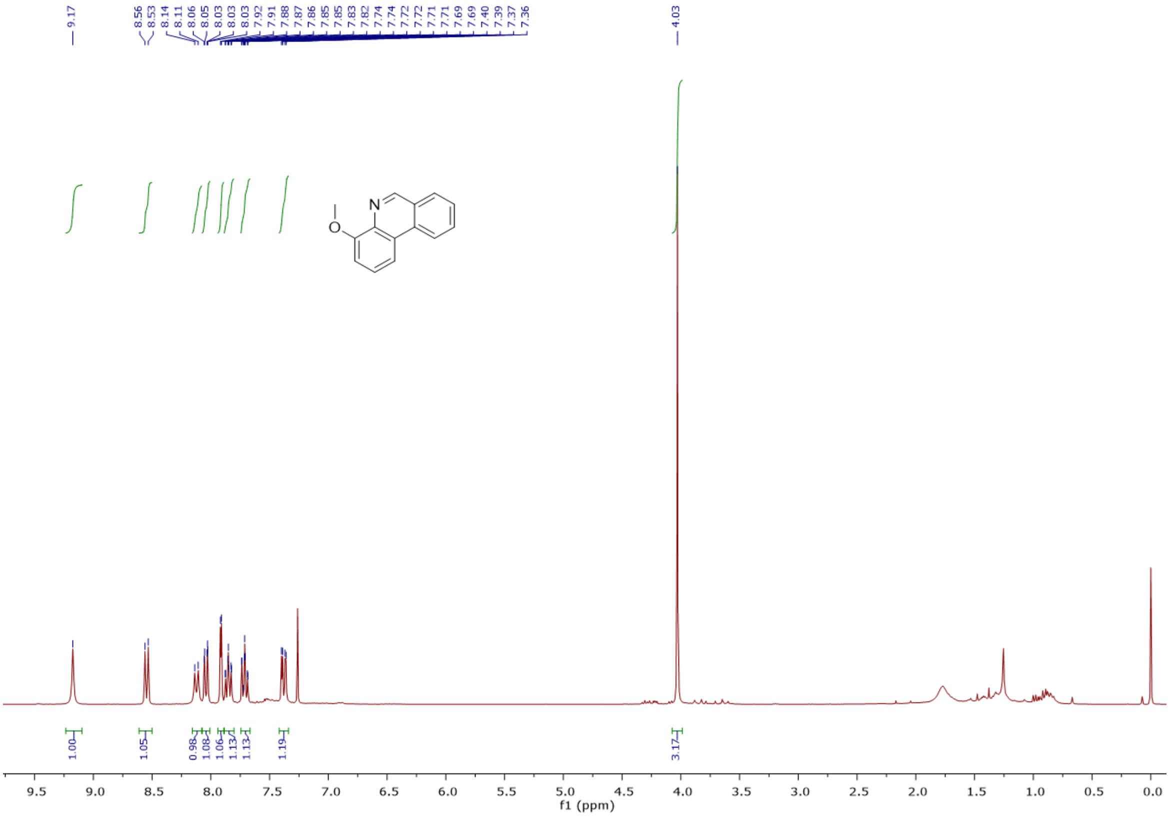

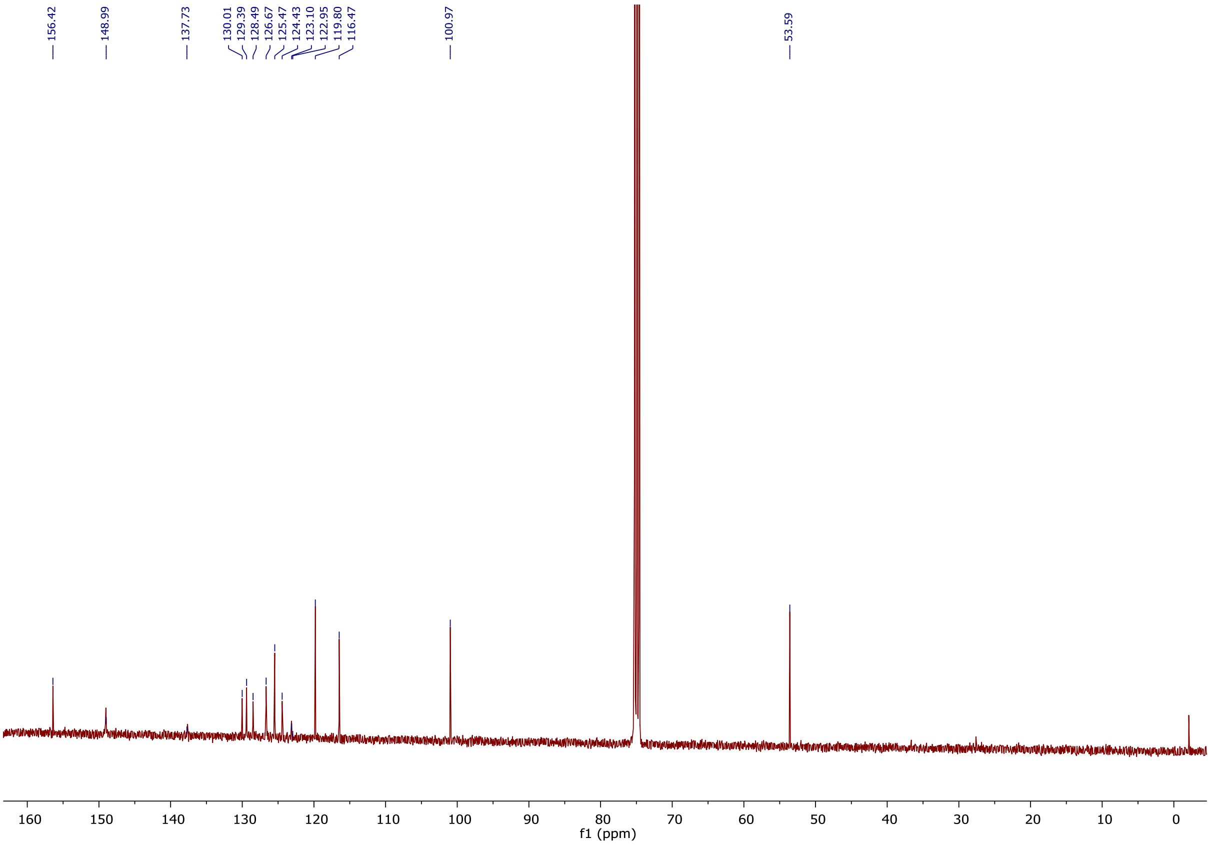

2',3'-Dimethoxy-[1,1'-biphenyl]-2-carbonitrile (16d)

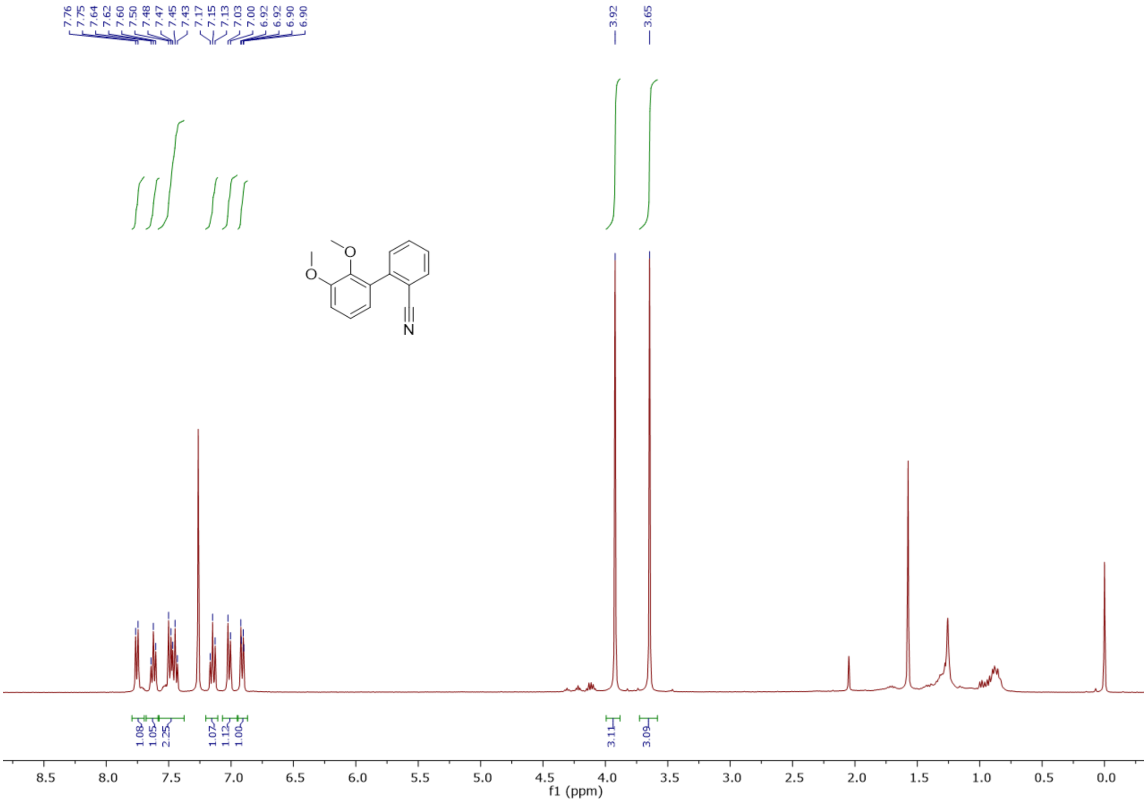

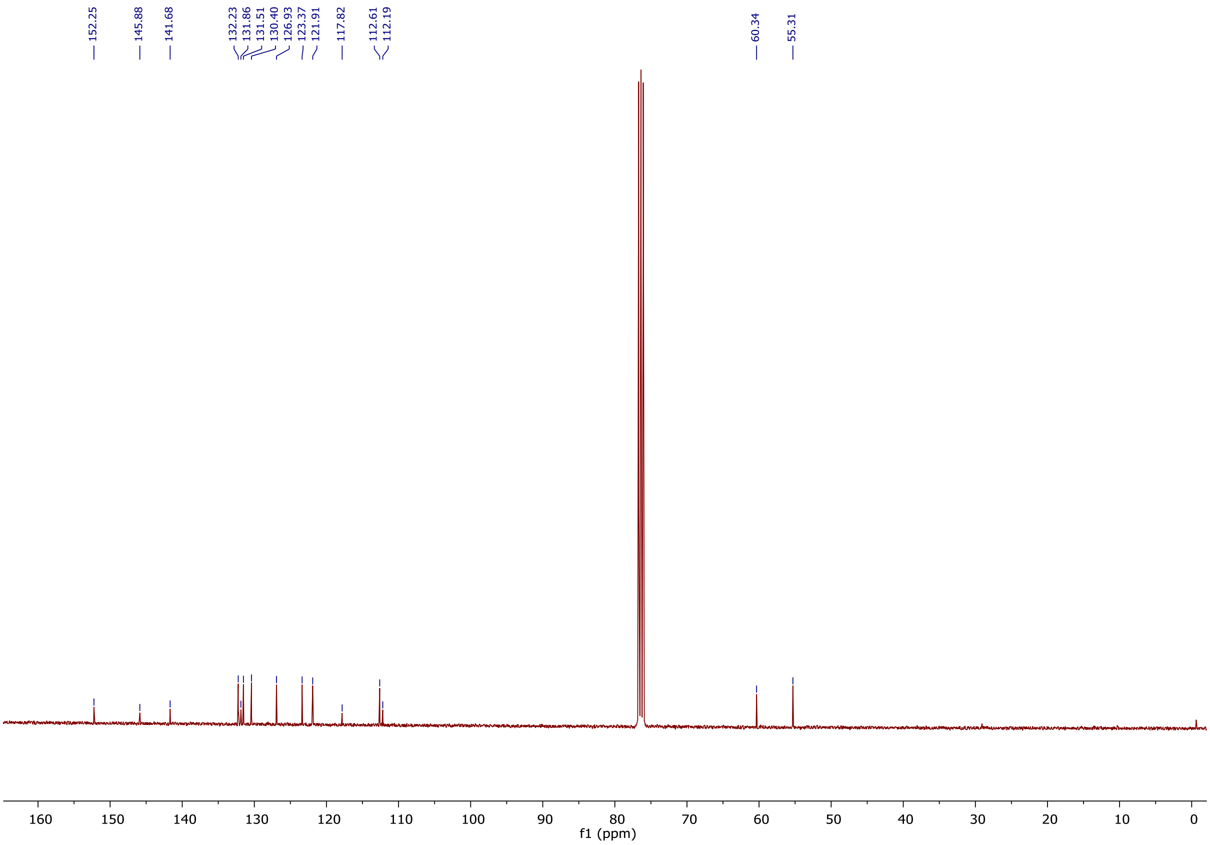

2',4'-Dimethoxy-[1,1'-biphenyl]-2-carbonitrile (16e)

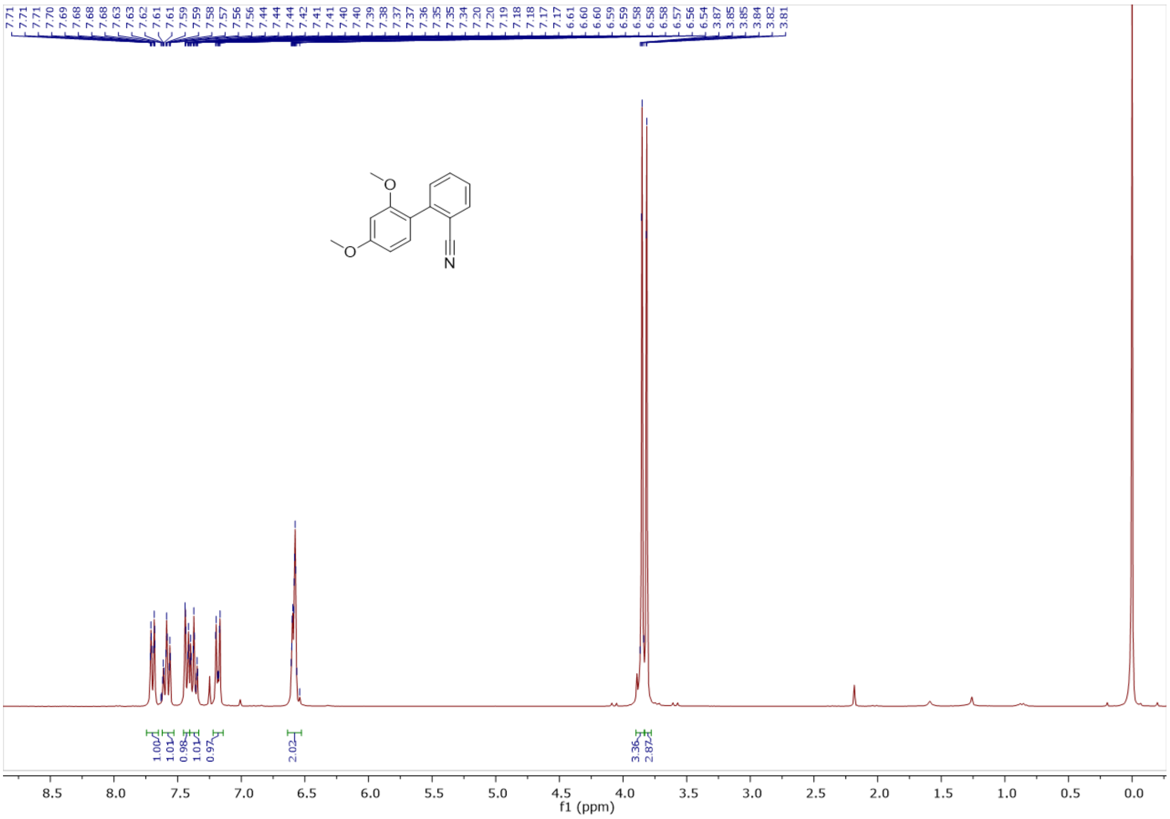

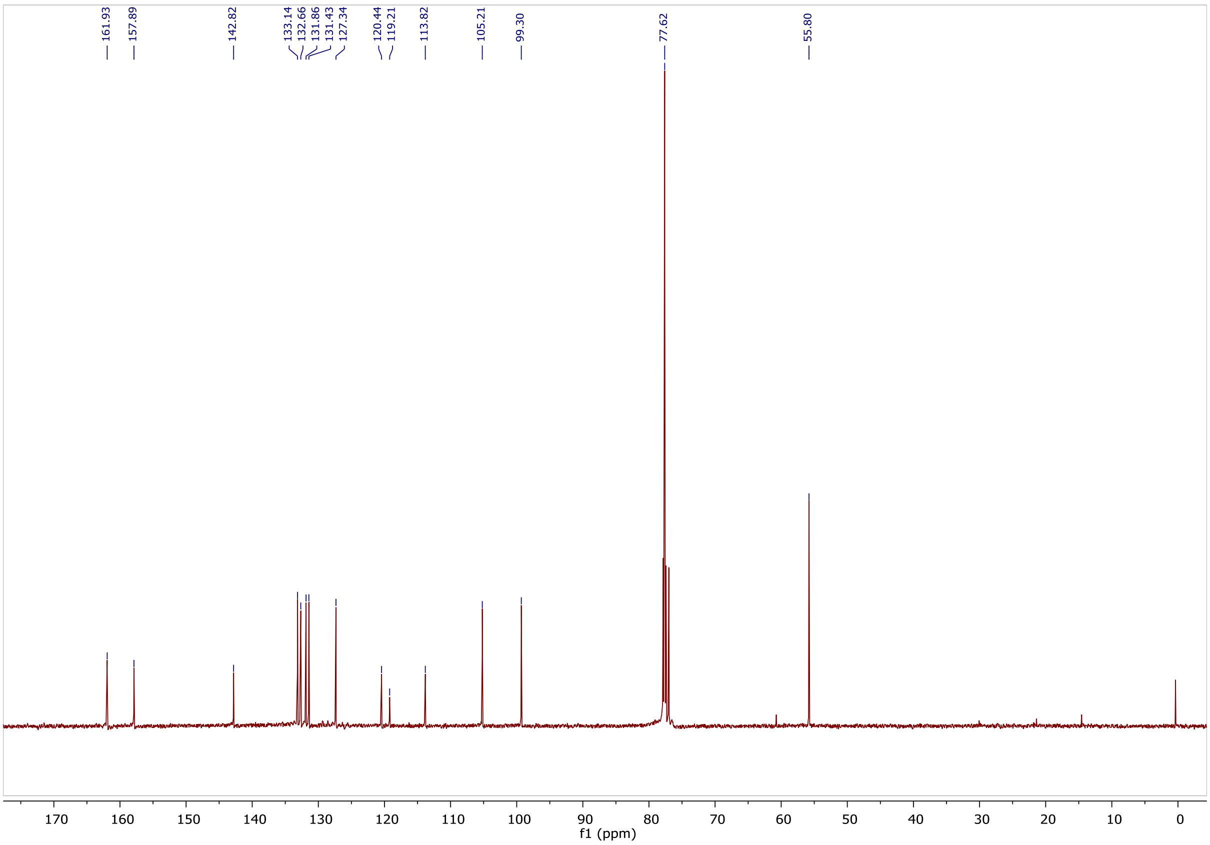

2'-Methoxy-[1,1'-biphenyl]-2-carbonitrile (16f)

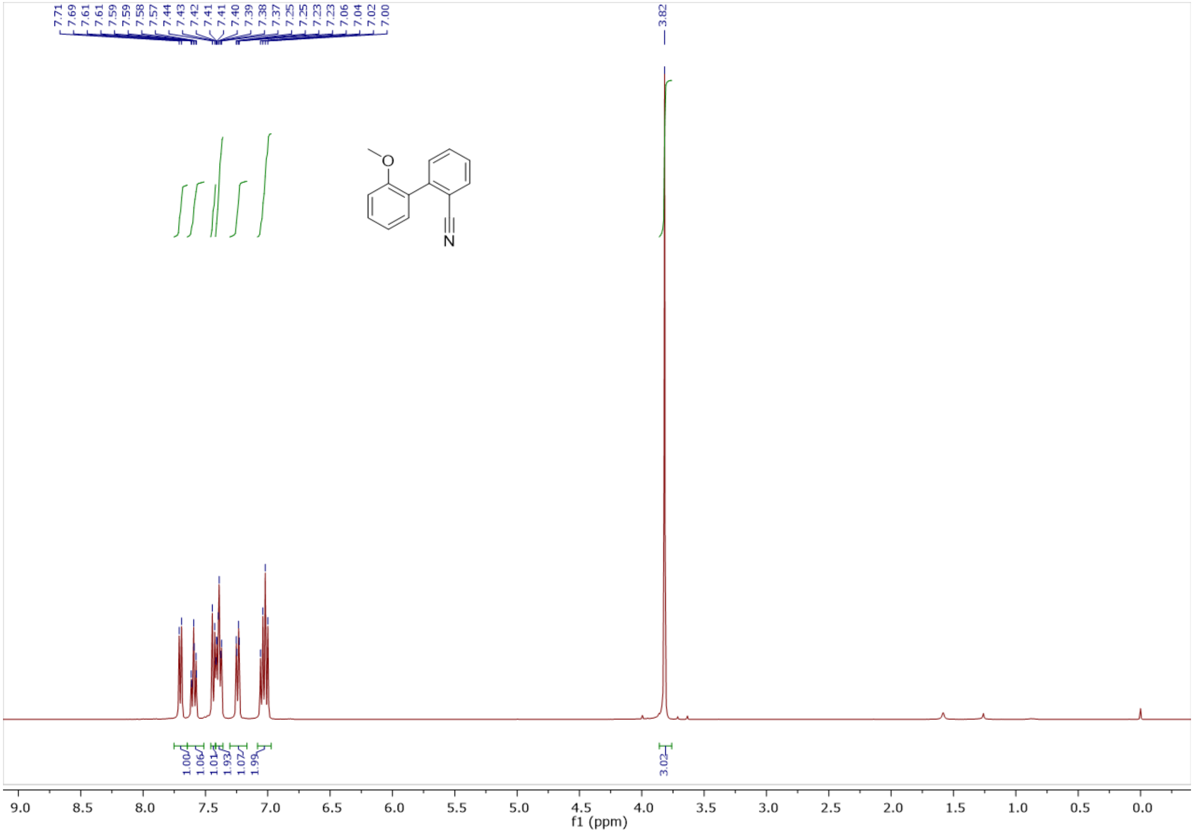

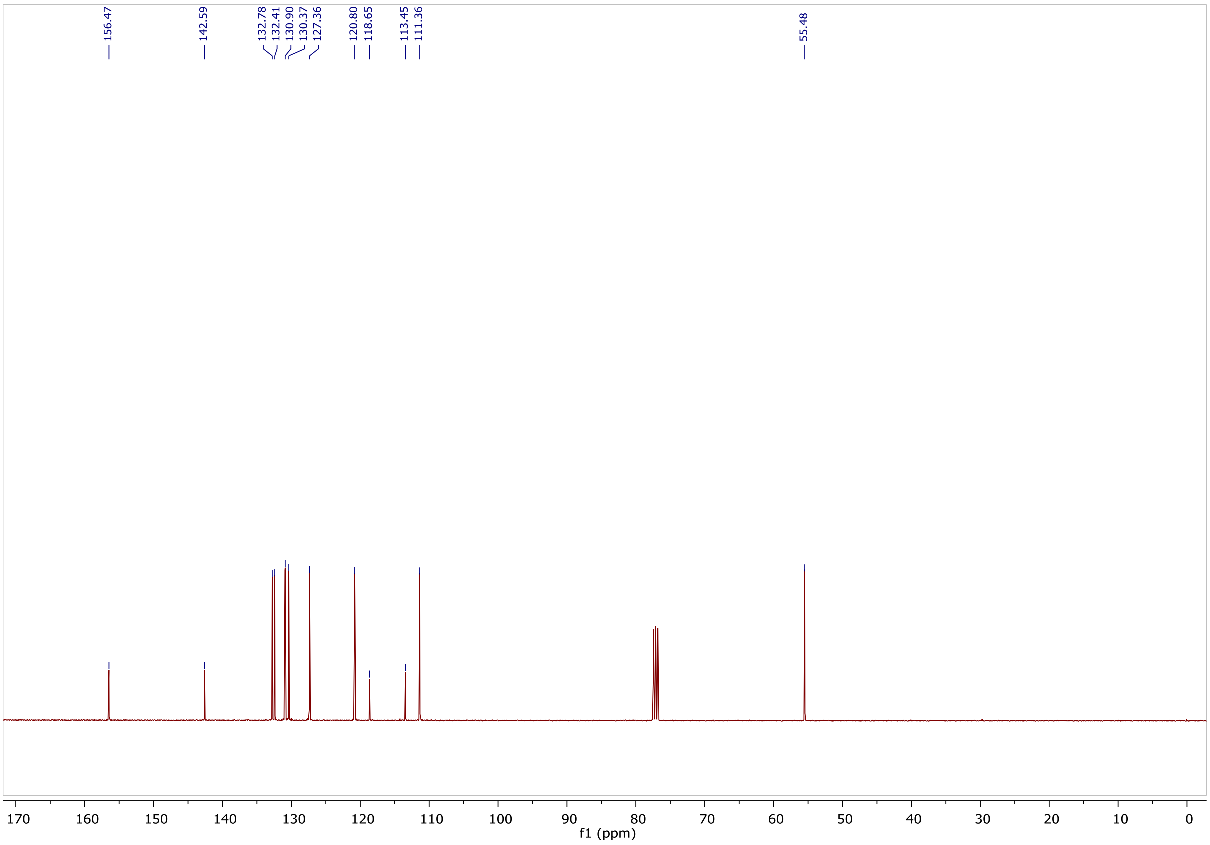

2,3-Dimethoxy-[1,3]dioxolo[4,5-*j*]phenanthridine (23)

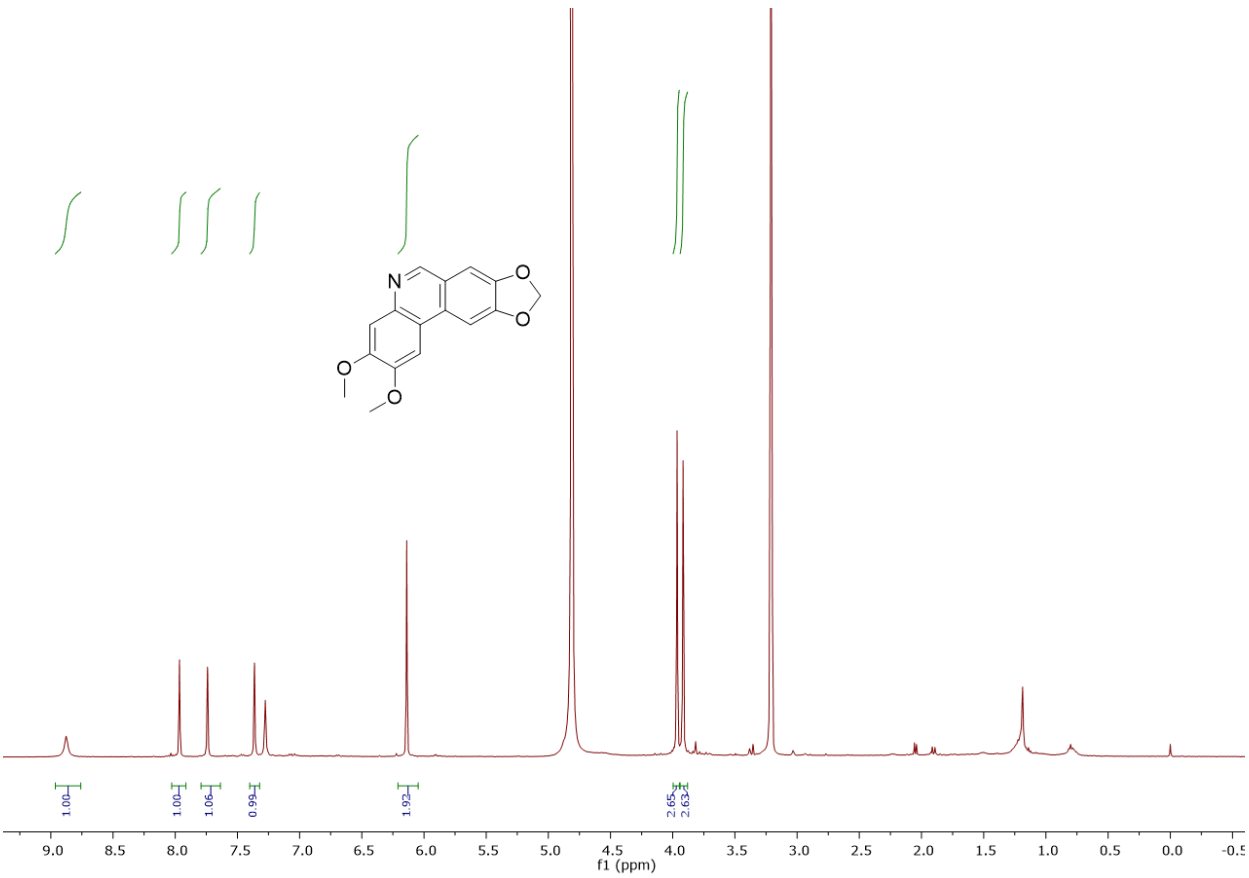

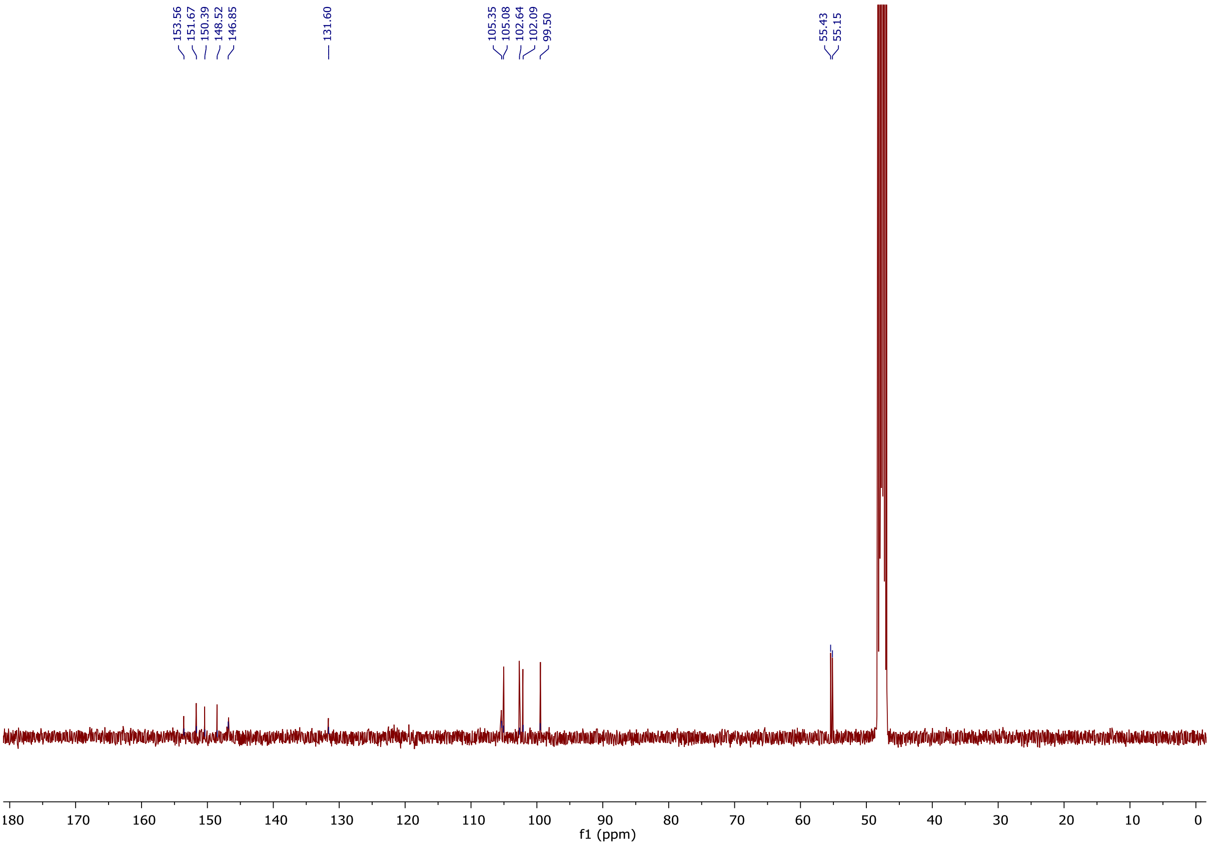

6-(2,4,5-trimethoxyphenyl)benzo[*d*][1,3]dioxole-5-carbonitrile (24)

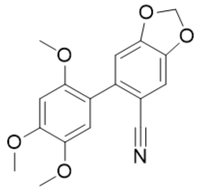

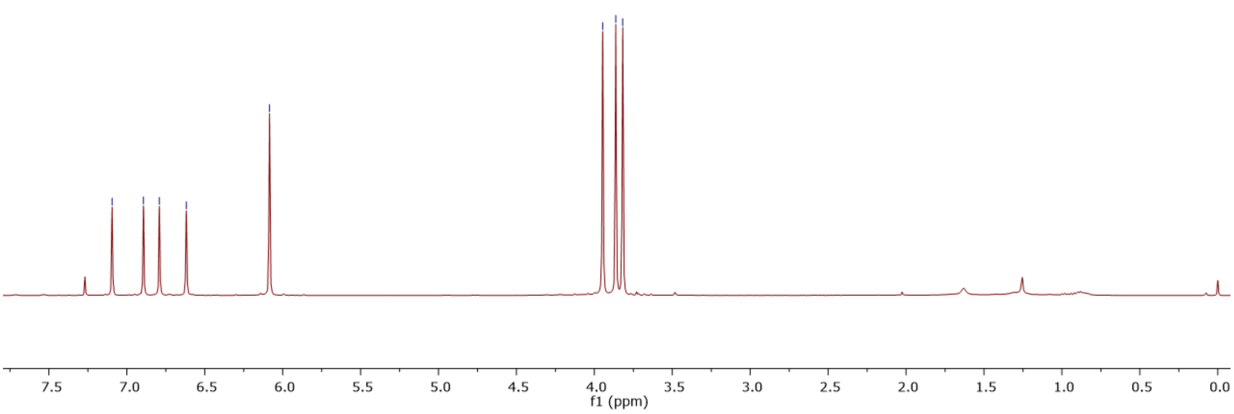

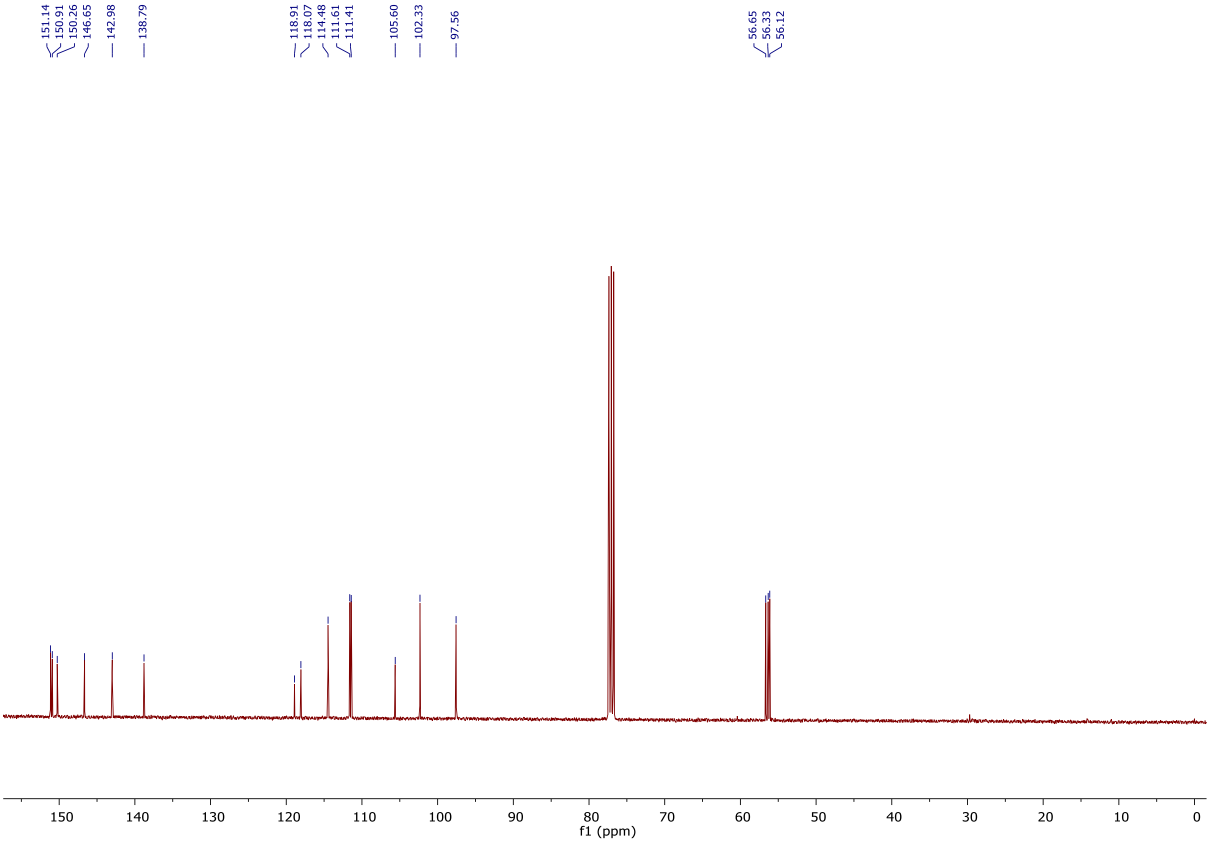

Trisphaeridine (3)

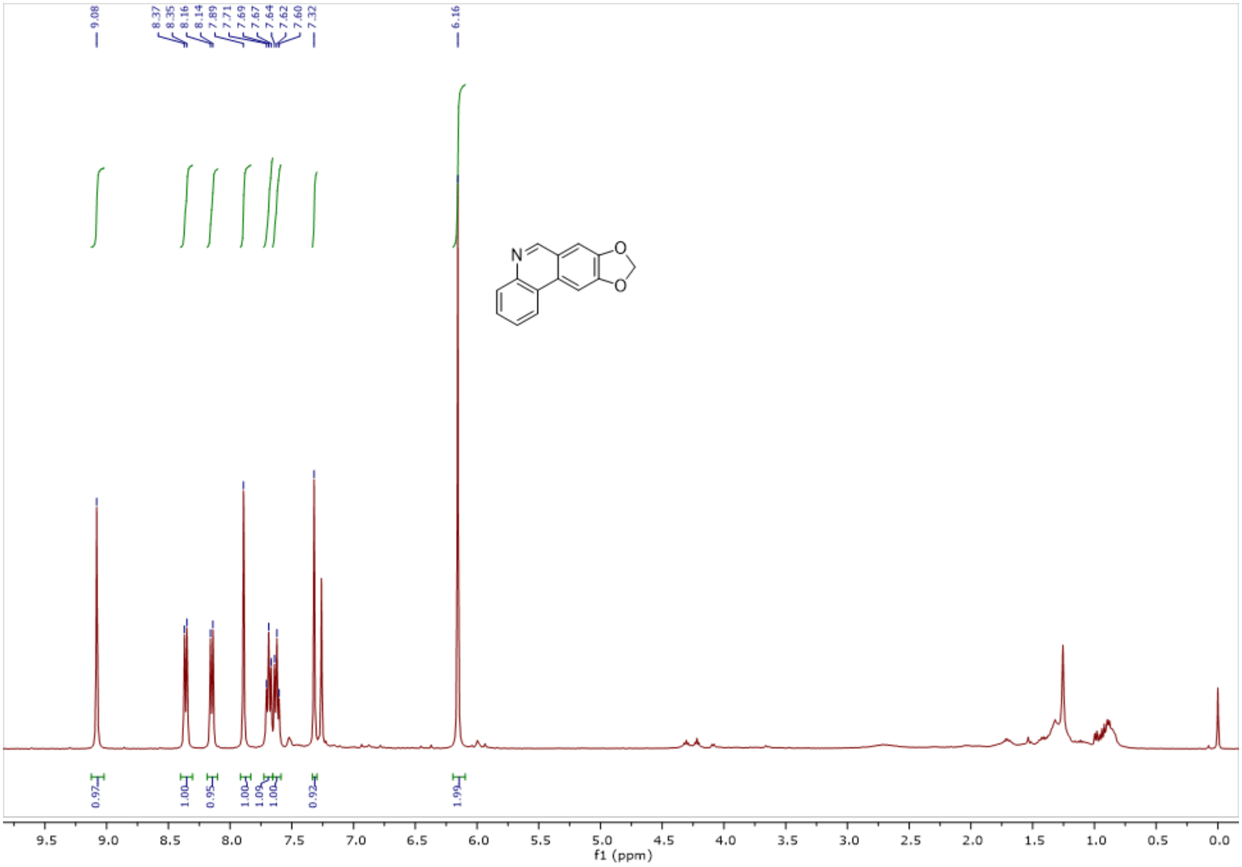

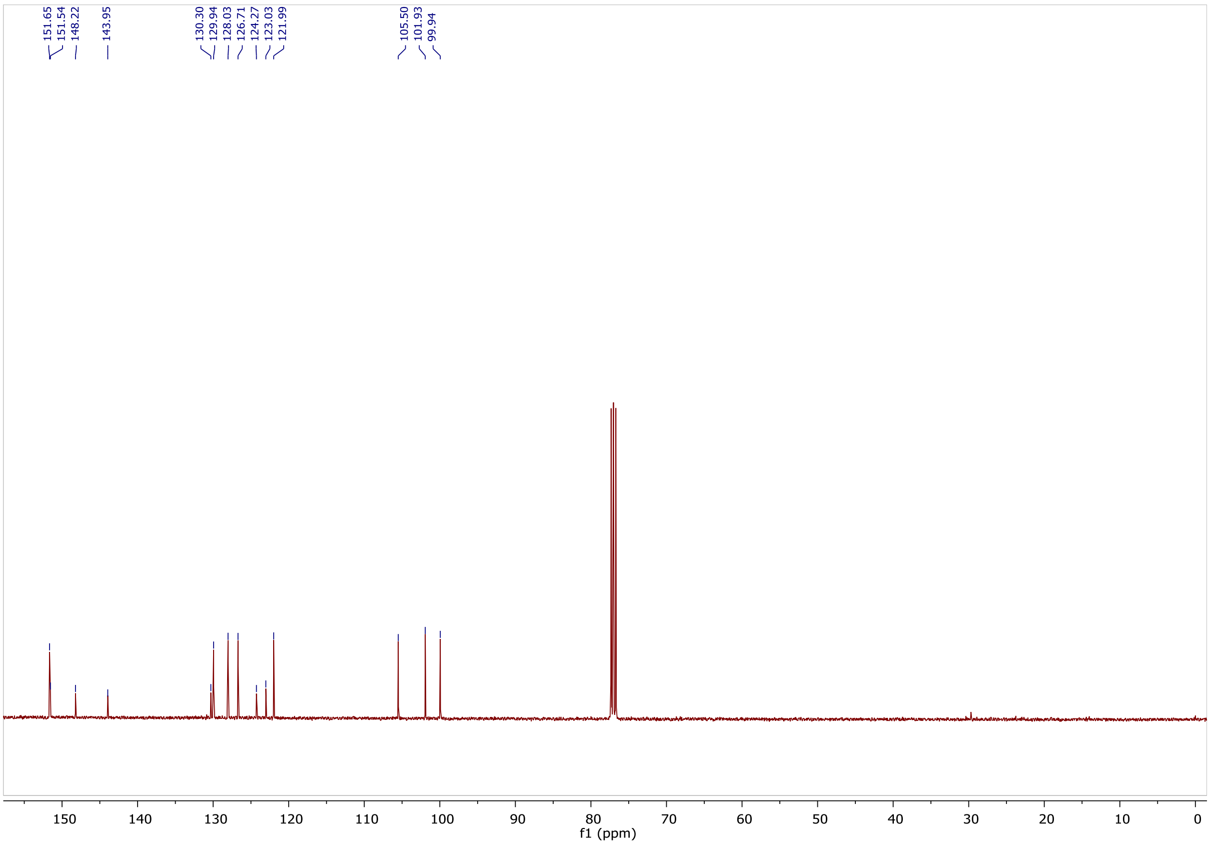

Supplement: File 1 — Experimental and analytical data. [file Beilstein_J_Org_Chem-17-2340-s001.pdf]
